# Direct observation of a spin-polarized surface Dirac gap in the antiferromagnetic topological insulator NdBi

**DOI:** 10.1038/s41467-026-77571-4

**Published:** 2026-09-15

**Authors:** Yuto Fukushima, Hiroyuki S. Suzuki, Masayuki Ochi, Takuma Nishida, Toshiya Ikenobe, Hiraku Saito, Yuto Kinoshita, Kaishu Kawaguchi, Hiroaki Tanaka, Ayumi Harasawa, Takushi Iimori, Koichiro Yaji, Masashi Tokunaga, Taro Nakajima, Kiyohisa Tanaka, Takahiro Morimoto, Ryo Mori, Takeshi Kondo

**Affiliations:** 1 Institute for Solid State Physics, The University of Tokyo, Kashiwa, Chiba Japan; 2 Department of Physics, The University of Osaka, Toyonaka, Osaka Japan; 3 Forefront Research Center, The University of Osaka, Toyonaka, Osaka Japan; 4 Maryland Quantum Materials Center, Department of Physics, University of Maryland, College Park, MD USA; 5 Center for Basic Research on Materials, National Institute for Materials Science (NIMS), 3-13 Sakura, Tsukuba, Ibaraki Japan; 6 Unprecedented-Scale Data Analytics Center, Tohoku University, 468-1 Aoba, Aramaki-Aza, Aoba-Ku, Sendai, Japan; 7 Trans-scale Quantum Science Institute, The University of Tokyo, Tokyo, Japan; 8 Institute of Materials Structure Science, High Energy Accelerator Research Organization, Tsukuba, Ibaraki Japan; 9 RIKEN Center for Emergent Matter Science, Wako, Japan; 10 SOKENDAI (The Graduate University for Advanced Studies), Okazaki, Japan; 11 UVSOR Synchrotron Facility, Institute for Molecular Science, Okazaki, Japan; 12 Department of Applied Physics, The University of Tokyo, Tokyo, Japan; 13 Department of Physics, Kyoto University, Kyoto, Japan

**Keywords:** Topological insulators, Condensed-matter physics

## Abstract

A gapped Dirac cone in antiferromagnetic topological insulators (AFM-TIs) is essential for realizing the quantized anomalous Hall effect, yet its intrinsic magnitude remains controversial. In the prototypical AFM-TI MnBi_2_Te_4_, however, the observed Dirac gap is significantly smaller than theoretical predictions or nearly absent, highlighting a long-standing discrepancy. Here we demonstrate that NdBi hosts a spin-polarized topological surface Dirac cone with a sizable gap of 17 ± 2 meV at the Dirac point, in excellent agreement with density functional theory predictions (12 meV). This direct observation is enabled by high-resolution 7 eV laser-based spin- and angle-resolved photoemission spectroscopy, which unambiguously disentangles the spin-polarized surface Dirac cone from bulk-derived states. Furthermore, through temperature dependence and controlled surface contamination, we establish a direct linkage between surface magnetism and the Dirac gap, demonstrating that the gap opens only in the presence of surface magnetic order. These results provide definitive spectroscopic evidence for an intrinsic Dirac gap in AFM-TIs and establish NdBi as a model platform to resolve the long-standing discrepancy between experiment and theory in AFM-TIs.

## Abstract (web summary)

In antiferromagnetic topological insulators, magnetic order opens a gap at the Dirac point, however, experimental confirmation of theoretical calculations of this magnetically induced gap has been challenging. Here, using angular resolved photoemission spectroscopy, Fukushima et al find a gapped surface state in NdBi, in close agreement with theoretical results.

## Introduction

Topological insulators (TIs) realize a striking quantum state in which the bulk remains insulating while the surface hosts gapless Dirac fermions protected by time-reversal symmetry. Most TIs discovered to date have been realized in spin-orbit-coupled semiconductors with weak electronic correlations. More recently, strongly correlated TIs have attracted significant attention as a fertile ground for realizing exotic topological phases, including topological superconductors, topological Kondo insulators, and topological Mott insulators^1–8^.

Antiferromagnetic topological insulators (AFM-TIs) provide a compelling platform for exploring correlated topological phases. In AFM-TIs, it is predicted that long-range magnetic order opens an energy gap at the Dirac point of the topological surface state (Dirac gap), leading to the emergence of one-dimensional chiral edge states along the crystal hinges^9^. The MnBi_2*n*_Te_3*n*+1_ family, the first identified AFM-TI^10^, has exhibited a variety of exotic responses, such as the quantum anomalous Hall effect and axion electrodynamics under magnetic fields^11–17^.

Despite these remarkable discoveries, a central challenge persists: the magnitude of the Dirac gap observed by angle-resolved photoemission spectroscopy (ARPES) is frequently inconsistent with theoretical predictions, and in many cases, absent^10,18–28^. Although early studies of MnBi_2_Te_4_ reported a sizable gap exceeding 50 meV^10,20^, later investigations clarified that it primarily originates from bulk states, while the surface Dirac cone remains nearly gapless even under magnetic order^21–25^. Furthermore, atomic-scale disorder and surface reconstruction have been indicated to strongly suppress the surface Dirac gap, rendering it highly sensitive to sample quality^26–29^. As a result, conclusive ARPES evidence of an intrinsic surface Dirac gap in AFM-TIs has remained elusive, especially in MnBi_2_Te_4_. Such a discrepancy limits the high-temperature applicability of these materials and obscures the understanding of the intricate interplay between bulk magnetism and topological surface state (TSS), which is central to correlated topological physics.

Recently, the type-A antiferromagnet NdBi with the Néel temperature (*T*_N_) of 24 K has been proposed as a second AFM-TI, and its topological properties have been intensively investigated^30,31^. NdBi belongs to a class of low-carrier-density semimetals with a band structure featuring multiple Dirac cones. The valence bands are mainly derived from Bi 6*p* orbitals, forming two distinct hole pockets. In contrast, the conduction bands originate from Nd 5*d* states and form electron pockets near the X points^32^. Due to strong spin-orbit coupling, the band gap narrows systematically from NdP to NdSb^30,31,33–37^, culminating in a band inversion in NdBi. This inversion underlies the topological phase transition in these materials.

The presence of an odd number of Dirac cones along the $\bar{\Gamma }$–$\bar{{{{{\rm{M}}}}}}$ direction—one at $\bar{\Gamma }$ and two at $\bar{{{{{\rm{M}}}}}}$^30^—underscores the topological nontriviality of NdBi and motivates detailed spectroscopic investigations. Beyond topology, rare-earth monopnictides have drawn considerable attention due to their strongly correlated effects in the low-carrier-density regime. The small Fermi energy (*E*_F_) in these systems weakens Thomas-Fermi screening, enabling long-range Coulomb interactions. As a result, the coupling between itinerant and localized *f* electrons becomes a defining factor in their physical properties^38^; a notable example is CeSb, which exhibits complex magnetic transitions known as the “devil’s staircase”^39–42^. The sister compound NdBi, therefore, provides an ideal platform for studying exotic AFM-TI phases driven cooperatively by magnetism and strong electron correlations in a low-carrier-density system. However, the effect of electron correlation on the topological properties, including the TSS, remains unclear.

A recent ARPES study on NdBi reported a sizable energy gap at the topological Dirac point as large as 125 meV below the Néel temperature^30^. This feature was attributed to time-reversal symmetry breaking induced by out-of-plane ferromagnetic order in the top surface layer (corresponding to the antiferromagnetic phase, Domain-C). In that work^30^, however, a large Dirac gap (~80 meV) is also reported even for the side surface (corresponding to the antiferromagnetic phase, Domain-A), where the Dirac point is expected to remain gapless due to symmetry protection. This ambiguity likely arises from the complex ARPES signals involving both surface and bulk states, reflecting a broader unresolved issue in AFM-TIs: the persistent discrepancy between experiment and theory, particularly regarding the nature and origin of Dirac gaps. Spin-resolved experiments that isolate the topological surface state from bulk states are essential to resolve this issue and fully understand the antiferromagnetic topological properties of this system.

In this work, we overcome these experimental limitations by employing high-resolution laser-based ARPES combined with spin-resolved measurements, enabling clear separation of the TSS from bulk states and its direct comparison with ab initio calculations. This approach provides evidence of the intrinsic surface Dirac gap of 17 ± 2 meV in NdBi that originates from the topological surface state, in agreement with theoretical predictions (12 meV). Our results resolve the persistent discrepancy between experiment and theory in AFM-TIs and reveal pronounced band reconstruction in the antiferromagnetic phase, underscoring the essential role of inherent magnetism in stabilizing gapped surface states. Although much smaller than previously reported^30^, the intrinsic Dirac gap we revealed in NdBi is still larger than the negligible gap in MnBi_2*n*_Te_3*n*+1_^21–25^. These results not only resolve a central puzzle in AFM-TI research but also establish NdBi as a promising platform for topological device engineering.

## Results

**Fig. 1:**
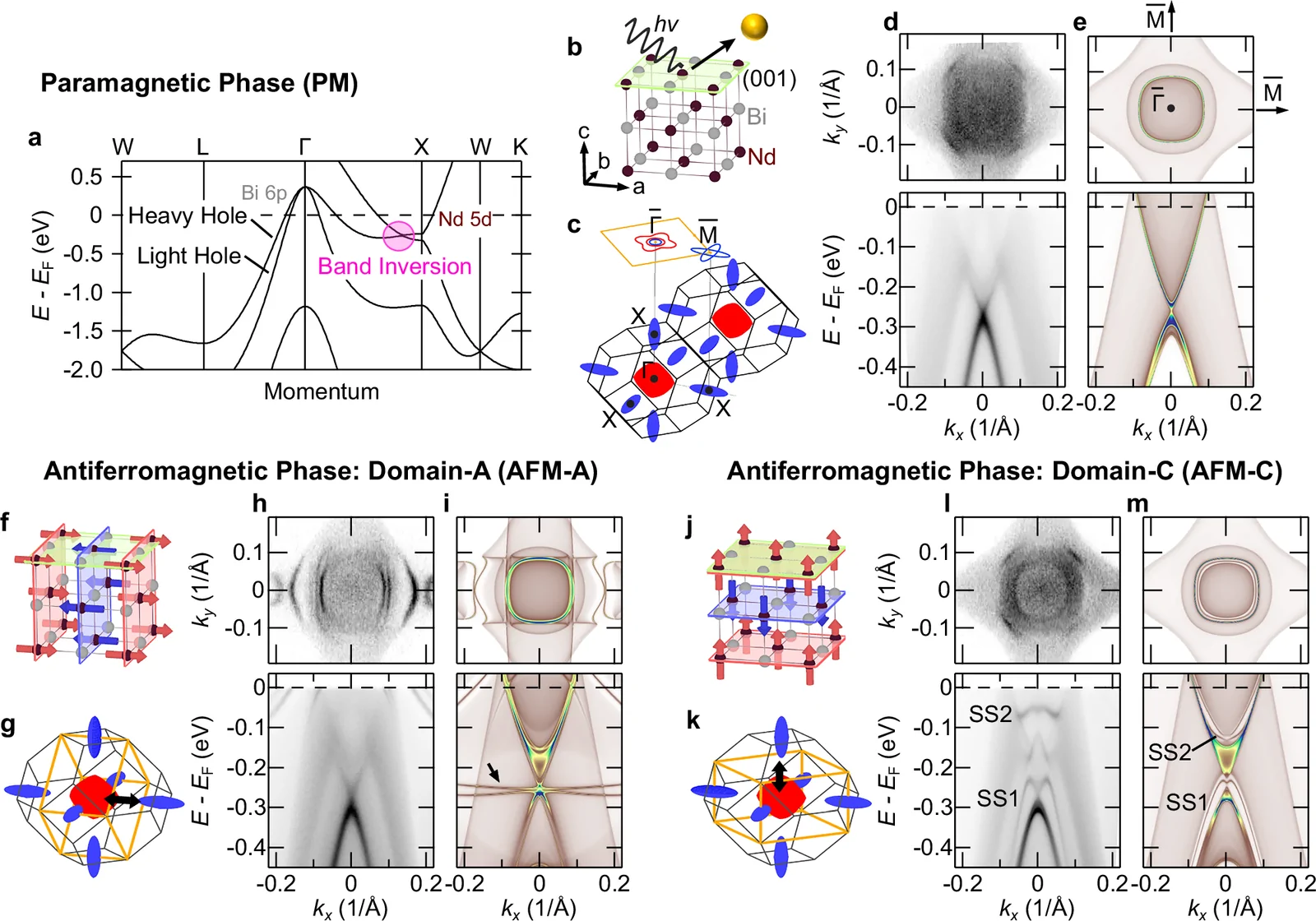
Electronic structure of NdBi across magnetic phases and domains. **a** Bulk band structure of NdBi in the paramagnetic (PM) phase, highlighting the band inversion between Bi 6*p* and Nd 5*d* orbitals. **b** Crystal structure of NdBi, with the cleavage plane shown in green. **c** Schematic of the bulk and surface Brillouin zones (BZs). **d** Fermi surface map and ARPES intensity plot of the band dispersion along $\bar{\Gamma }$--$\bar{{{{{\rm{M}}}}}}$ of NdBi measured by ARPES in the paramagnetic phase. **e** Fermi surface and band dispersion along $\bar{\Gamma }$--$\bar{{{{{\rm{M}}}}}}$ calculated using density functional theory (DFT). **f** Magnetic structure of the AFM-A domain. **g** Folded BZ due to magnetic ordering along [100]. **h** Fermi surface map and band dispersion along $\bar{\Gamma }$--$\bar{{{{{\rm{M}}}}}}$ measured by ARPES. **i** Corresponding DFT results. **j–m** Same as (**f**–**i**), but for the AFM-C domain. Yellow lines in the (**g**, **k**) represent the folded BZ. Two surface states (SS1 and SS2) are resolved in the AFM-C phase (**l**, **m**).

We begin by presenting, in Fig. 1, the electronic structures around $\bar{\Gamma }$ above (Fig. 1b–e) and below (Fig. 1f–i, 1j–m) *T*_N_ obtained by ARPES and density functional theory (DFT) calculations. Below *T*_N_, the magnetic moments of Nd 4*f* electrons are ordered ferromagnetically within the (001) plane and stacked antiferromagnetically along the [001] direction [Fig. 1f, j]. Therefore, three magnetic domains are created with antiferromagnetic (AFM) moments aligned along the *a*, *b*, and *c* axes, for which we label AFM-A, AFM-B, and AFM-C, respectively. These magnetic domains can be distinguished from the symmetry of the Fermi surface near the $\bar{\Gamma }$ point. To reveal the electronic structure of individual magnetic domains, we employed ARPES with a focused 7 eV laser beam (~50 μm in spot size), which enables not only high-resolution measurements, but also magnetic-domain-selective observations by spatially scanning the small beam spot across the (001) cleavage surface.

The paramagnetic (PM) phase has three sheets of Fermi surfaces around $\bar{\Gamma }$ in the bulk state (Fig. 1c): one square extending in the $\bar{\Gamma }$–$\bar{{{{{\rm{M}}}}}}$ direction, another smaller square, and the smallest circular one at the center. Our DFT calculations (Fig. 1a, e) identify that these Fermi surfaces correspond to the heavy hole, light hole, and electron pocket, respectively. In Fig. 1d, e, ARPES intensity maps for energy dispersion and Fermi surface are compared with surface calculations, which contain both bulk states projected onto the crystal surface and intrinsic surface states. In ARPES spectra, bulk bands with strong *k*_*z*_ dispersion appear as a broad continuum. This is due to *k*_*z*_ broadening arising from the surface sensitivity of photoemission. The data and calculations consistently show highly dispersive Dirac-like dispersion characterized by sharp-peak spectra. Although this Dirac-like band has been interpreted as a topological surface Dirac cone in prior studies, its nature remains ambiguous and will be revisited in a later section.

In AFM-A (Fig. 1f), the magnetic moments are ordered in the [100] direction, and the Brillouin zone (BZ) is folded back along *k*_*x*_, resulting in the appearance of two-fold Fermi surfaces in the bulk states [Fig. 1h, i]. In addition to the band folding effect, additional surface states with sharp spectra emerge and cross the Fermi level, consistent with previous reports. In the DFT calculation, two extra branches are seen around −0.3 eV [black arrow in Fig. 1i], which, however, were not observed by ARPES likely due to the matrix-element effect. It is worth noting that AFM-B shares the same electronic structure as AFM-A, but rotated by 90 degrees (see Supplementary Note 2).

In AFM-C [Fig. 1j], the magnetic moment is aligned perpendicular to the cleavage plane, causing the BZ to fold back along *k*_*z*_. Unlike AFM-A and AFM-B, AFM-C retains *C*_4_ symmetry over the cleavage plane even under the magnetic order. While the Fermi surface [Fig. 1l, m] hardly changes from the PM phase, two surface bands (SS1, SS2) emerge, as also reported in previous studies^43^. Our DFT calculations reproduce these surface states, supporting that the observed domain is indeed the AFM-C domain.

**Fig. 2:**
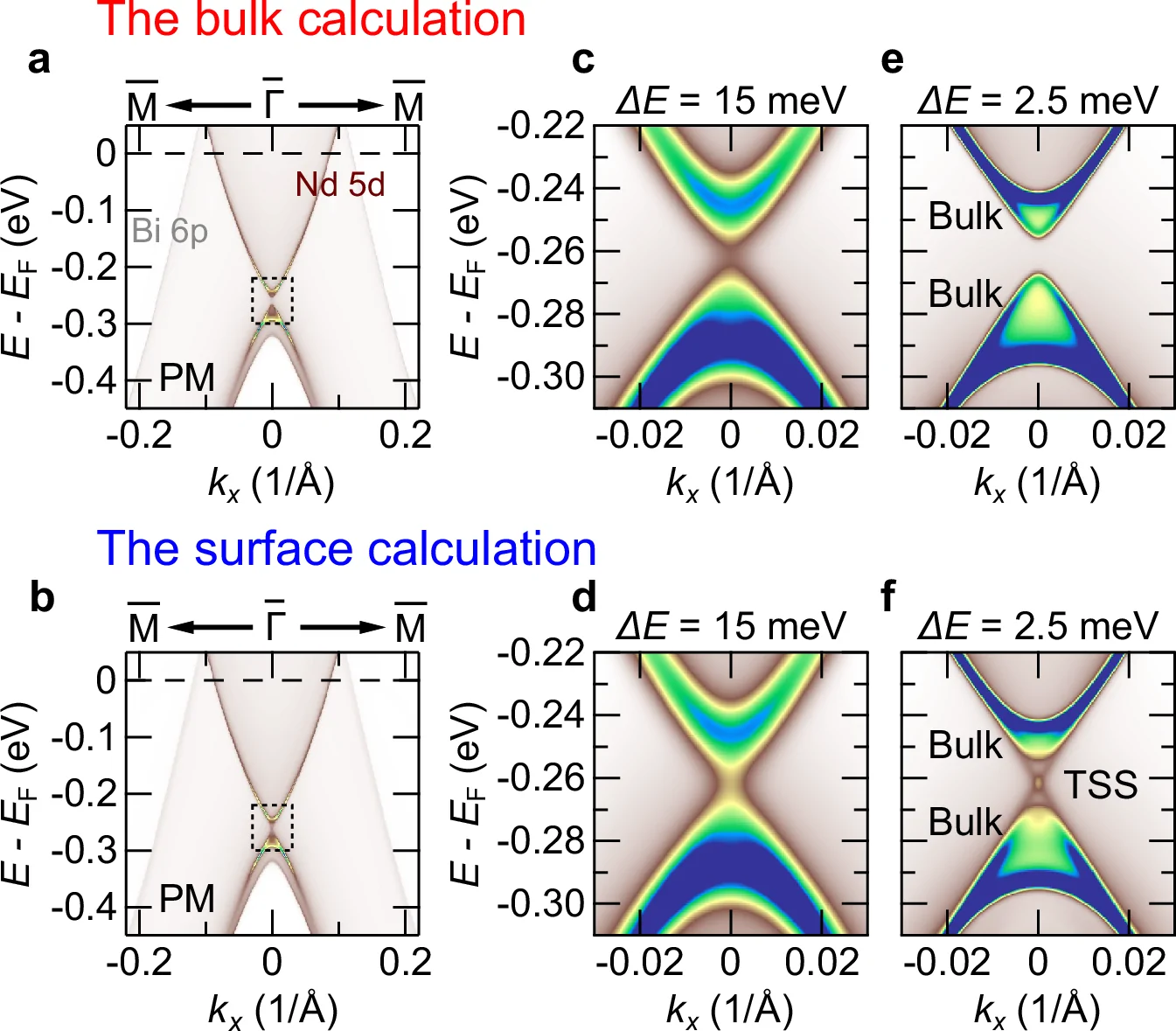
Energy resolution effect on the bulk band and the surface Dirac cone in NdBi. **a**, **b** DFT-calculated band dispersion of paramagnetic phase along the $\bar{\Gamma }$--$\bar{{{{{\rm{M}}}}}}$ direction, showing the spectral weight of bulk (**a**) and surface (**b**) states. **c**, **d** Simulated ARPES images showing the impact of energy resolution on the visibility of the bulk band gap marked by a dotted rectangle. The calculations in (**a**, **b**) are convoluted with energy resolutions of 15 meV in (**c**, **d**), and 2.5 meV in (**e**, **f**), respectively. The topological surface state (TSS) is only clearly resolved in the surface calculation when using high energy resolution (2.5 meV), as shown in (**f**).

**Fig. 3:**
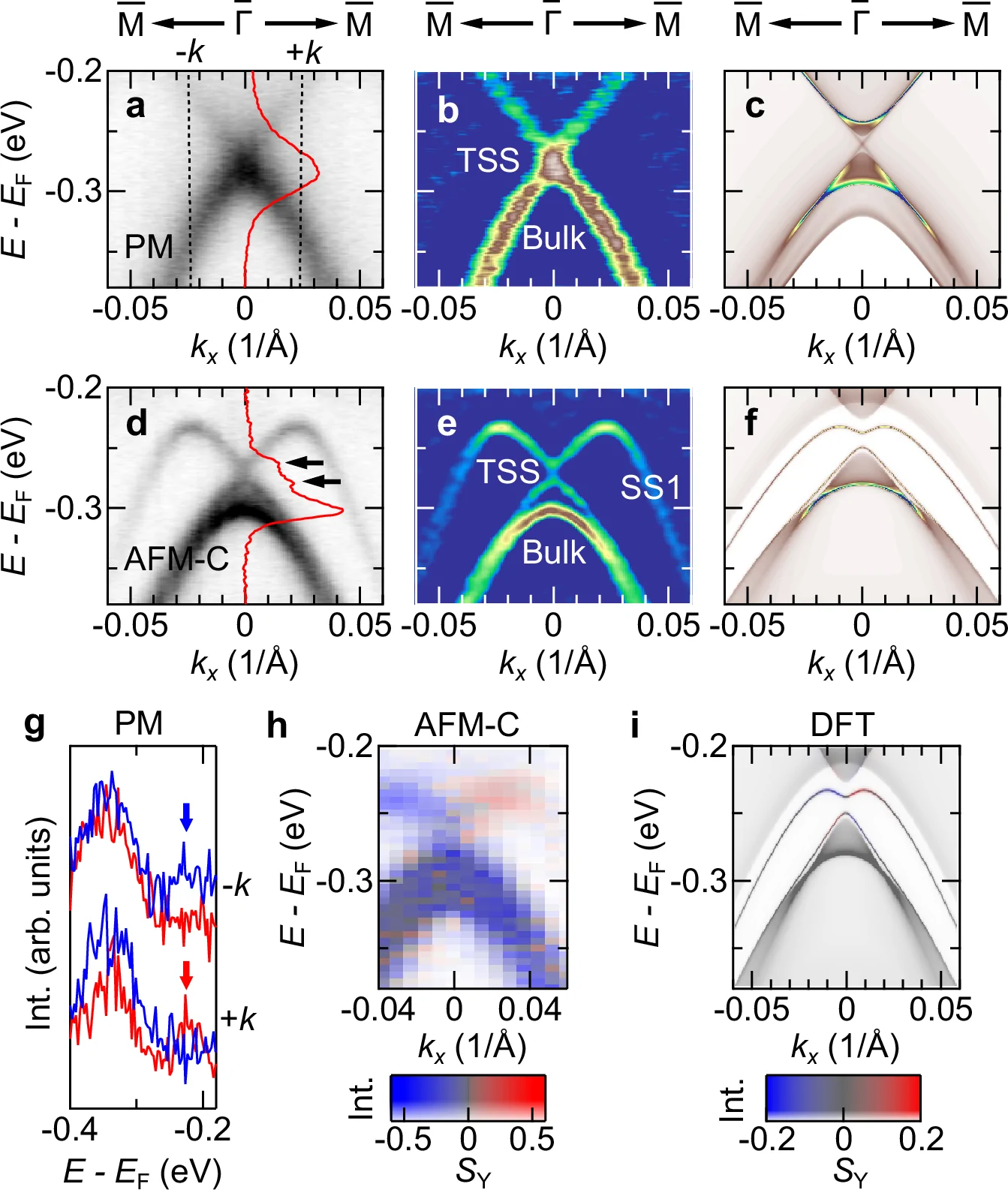
Observation of a gapped surface Dirac cone entangled from the bulk band. **a** High-resolution ARPES image along the *k*_*x*_ direction (*k*_*y*_ = 0 Å^−1^) measured in the paramagnetic phase (*T* = 44 K). The energy distribution curve (EDC) at $\bar{\Gamma }$ is overlaid in red. **b** Curvature plot of the ARPES data shown in (**a**). **c** DFT-calculated band structure in the paramagnetic phase. **d–f** Same as **a–c** but for the AFM-C domain (*T* = 13 K), showing the emergence of an M-shaped surface band and the opening of a Dirac gap. The EDC in **d** shows two peaks at $\bar{\Gamma }$ (black arrows), indicating the gap. **g** Spin-resolved EDC spectra at +*k* and −*k* positions (as indicated in **a**), showing opposite spin polarizations. Red and blue lines represent up and down spin components, respectively. **h** Spin-resolved band dispersion measured in the AFM-C domain. **i** Corresponding spin-polarized DFT calculation, confirming the spin texture of the gapped surface Dirac cone.

**Fig. 4:**
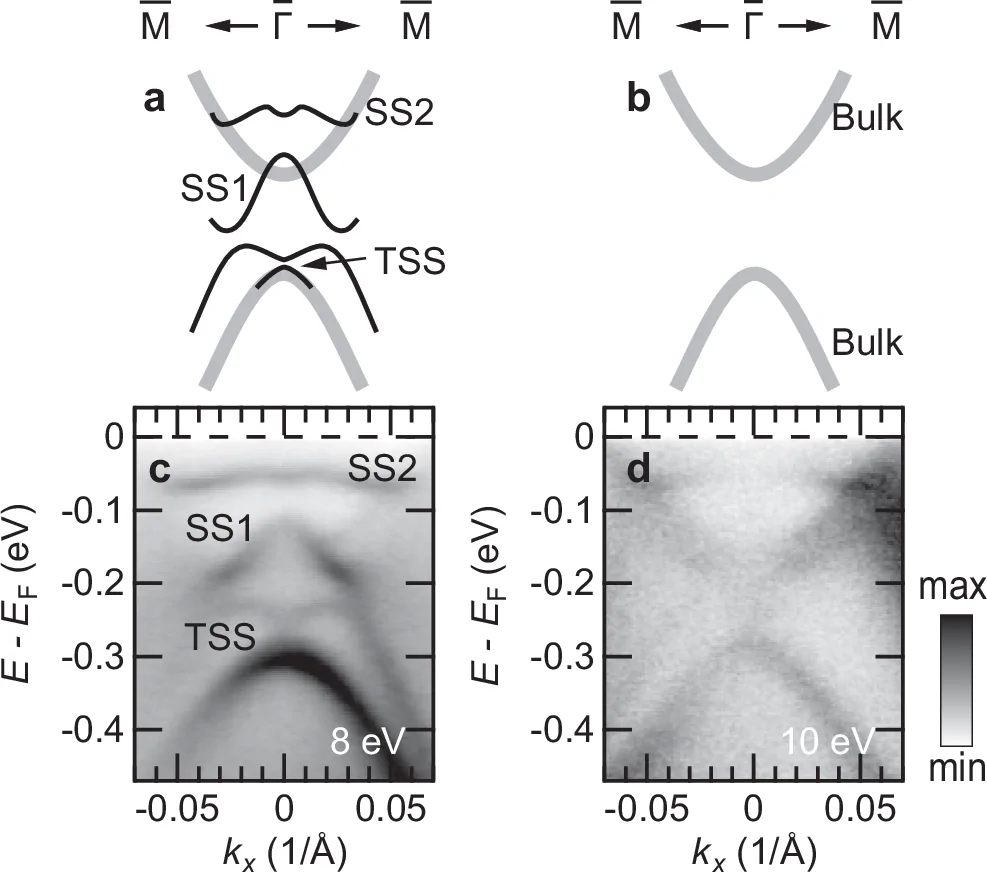
Suppression of surface state ARPES signals at higher photon energies. **a**, **b** Schematic band dispersions representing both surface states (TSS, SS1, and SS2) and bulk states (**a**), and only bulk states (**b**), corresponding to **c** and **d**, respectively. **c**, **d** ARPES intensity maps along the $\bar{\Gamma }$--$\bar{{{{{\rm{M}}}}}}$ direction measured at photon energies of 8 eV (**c**) and 10 eV (**d**). While both surface and bulk bands are observed at the lower photon energy (8 eV), matrix-element effects strongly suppress the surface-state intensity at the higher photon energy (10 eV), dominantly leaving only the signals from the bulk states, as illustrated in (**a**, **b**), respectively.

The basic electronic structures presented above have been reported before^30,43–46^. The main results of this work are the observation of the Dirac gap and the intrinsic interplay between bulk and surface states. Before delving into the details presented in Figs. 3 and 4, we highlight a key challenge in Fig. 2: the difficulty in distinguishing surface and bulk states. Overcoming this difficulty is crucial for identifying a genuine TSS and, thus, the intrinsic Dirac gap in NdBi.

In ARPES research, surface states are typically assigned to states that show no signs of *k*_*z*_ dispersion. We found that in NdBi, some of the bulk bands exhibit negligible *k*_*z*_ dispersion, and significantly, the 2D-like bulk band displays an energy dispersion resembling a Dirac cone. To clarify this, in Fig. 2a, we calculated the bulk bands of the PM phase and integrated them over the entire *k*_*z*_. Although this calculation does not include surface states, the obtained results show Dirac-like bands consisting of sharp peak spectra. For comparison, we performed the surface calculations in Fig. 2b, which not only exhibit the bulk band projected onto the surface but also surface states. Our results indicate that the topological surface Dirac cone emerges only in a narrow energy window within the band gaps of the Dirac-like bulk bands. Since the bulk band gap is only ~13 meV, standard ARPES with a typical energy resolution of ~15 meV can hardly distinguish the genuine surface Dirac cone from the bulk bands. As simulated in Fig. 2f, the surface Dirac cone can be identified when the resolution reaches a level of 2.5 meV (as employed in this work).

The above consideration suggests that unveiling the topological surface Dirac cone requires high-resolution measurements and the capability to distinguish between surface and bulk states, advancing beyond previous studies^30,31,36^. Our laser-based ARPES technique provides sufficiently high-resolution (Δ*E* = 2.5 meV) to distinguish them (Fig. 2f). A further decisive approach is to use the spin-resolved ARPES. Because the TSS is spin-polarized, whereas the bulk states are not, spin-resolved ARPES could disentangle the complex interplay between surface and bulk states in NdBi.

In Fig. 3a, we show the high-resolution ARPES image obtained in the PM phase. A crossing point around −0.27 eV indicates the band as the Dirac cone. Nevertheless, the energy distribution curve (EDC) at $\bar{\Gamma }$ (red curve), overlaid in the image, exhibits a shoulder-like feature rather than a clean single peak. As discussed in Fig. 2, one needs to consider the contribution of Dirac-like bulk states to the data.

To resolve this, we performed spin-ARPES and plotted in Fig. 3g the obtained spin-resolved EDCs at positive and negative momenta +*k* and −*k* (dotted lines in Fig. 3a) equidistant from $\bar{\Gamma }$. We found that the upper part of the Dirac cone exhibits spin flipping across *k*_*x*_ = 0 Å^−1^. In contrast, the bottom part of the band does not exhibit clear spin flipping, as it is dominated by the bulk band of the light hole band, which contributes significantly to the spectral intensity. These observations conclude that a spin-polarized Dirac cone indeed exists. A more detailed discussion of the bulk-state contribution to the measured spin polarization is provided in Supplementary Note 9.

The spectral relationship between the surface Dirac cone and the bulk states in the AFM-C domain (Fig. 3d) is essentially the same as in the PM phase (Fig. 3a); while the upper bands correspond to the surface state, the lower bands consist of both surface and bulk states that can be distinguished only in a narrow energy-momentum window. However, a clear difference from the PM phase is observed in the shape of the surface band. Intriguingly, the upper Dirac cone, which was V-shaped in the PM phase, transformed into an M-shaped dispersion in the AFM-C domain. As revealed later (Fig. 4a), the M-shaped dispersion is the product of hybridization between the original V-shaped upper Dirac cone and a hole-like surface band, which emerges under antiferromagnetism. The features of these bands are further examined by spin-resolved ARPES in Fig. 3h. The upper cone clearly exhibits spin polarization that reverses across $\bar{\Gamma }$, while the lower cone, which almost overlaps with bulk states, is not clearly resolved. The magnitude of spin polarization in the upper cone is approximately 20% and gradually decreases with increasing *k*_*x*_, as reproduced by our spin-dependent DFT calculations [Fig. 3i]. This behavior can be attributed to the non-spin-polarized SS1, which hybridizes with the TSS, giving rise to the M-shaped dispersion.

Most importantly, we found a two-peak structure in the EDC at $\bar{\Gamma }$ (red curve overlaid in Fig. 3d), as marked by the black arrows, in addition to the intense peak of the bulk band. These two peaks indicate the opening of a gap at the Dirac point. The curvature plot in Fig. 3e offers a clearer view of the gap opening in the AFM-C. Compared with the PM phase (Fig. 3b), the lower Dirac cone becomes better discernible (Fig. 3e), as the bulk band shifts downward in the AFM-C domain. In these experiments, we used an ARPES analyzer with a deflector, which allows a precise determination of the $\bar{\Gamma }$ point. In addition, the low-energy photons of the 7 eV laser we use enable us to minimize the angle integration for each EDC detection, thus achieving measurements with high momentum resolution. Under this carefully controlled experimental setup, we consistently obtained the same gap size (17 ± 2 meV) from several different samples.

The gap size (17 ± 2 meV) identified here for the surface Dirac cone at $\bar{\Gamma }$ is an order of magnitude smaller than the previously reported value (~125 meV)^30,31^. This discrepancy likely stems from misidentifying the Dirac-like bulk bands shown in Fig. 2 as surface states. This issue arises not only from limited energy resolution but also from matrix element effects, which are sensitive to the photon energy used in ARPES. To examine this, we measured the photon-energy dependence of the band-dispersion signals in NdBi at a synchrotron facility specialized in low-photon-energy experiments (Fig. 4). At a low photon energy (*hν*) of 8 eV (Fig. 4c), close to *hν* = 7 eV in our laser-ARPES, the surface states (TSS, SS1, and SS2) are clearly observed with high intensity. In contrast, at a higher *hν* of 10 eV (Fig. 4d), these surface states almost entirely vanish, and the spectral weight is dominated by bulk bands (Fig. 4a, b). This photon-energy dependence is a consequence of matrix-element effects, by which lower photon energies more effectively excite the TSS in NdBi. This implies that the large gap (~125 meV) reported in previous studies at *hν* = 60 eV^30,31^ likely corresponds to a bulk band gap rather than the topological surface Dirac gap.

Notably, similar matrix-element effects have been reported in MnBi_2_Te_4_, which is well established as an AFM-TI^21,22,47^. The TSS in that system is also clearly resolved only at low photon energies, whereas at higher photon energies (e.g., above ~20 eV), the ARPES spectra are dominated by bulk bands. This can lead to an apparent large gap originating from bulk states being misinterpreted as the topological Dirac gap, even though the intrinsic TSS gap is much smaller. The close analogy between MnBi_2_Te_4_ and NdBi underscores that low-*hν* ARPES is very effective for directly accessing the intrinsic topological surface states in AFM-TIs.

Magnetic transitions in antiferromagnets are typically viewed as simple on-off switches between PM and ordered phases. The richness of their magnetic behavior can also be unveiled by the intermediate-temperature evolution. In particular, tracing how the M-shaped surface band (Fig. 3d) gradually develops as the interaction between the original TSS (the surface Dirac cone) and the emerging surface state below *T*_N_ offers new insight into the temperature-driven dynamic interplay between topology and magnetism. Our high-resolution ARPES measurements uniquely revealed this previously unexplored temperature evolution.

**Fig. 5:**
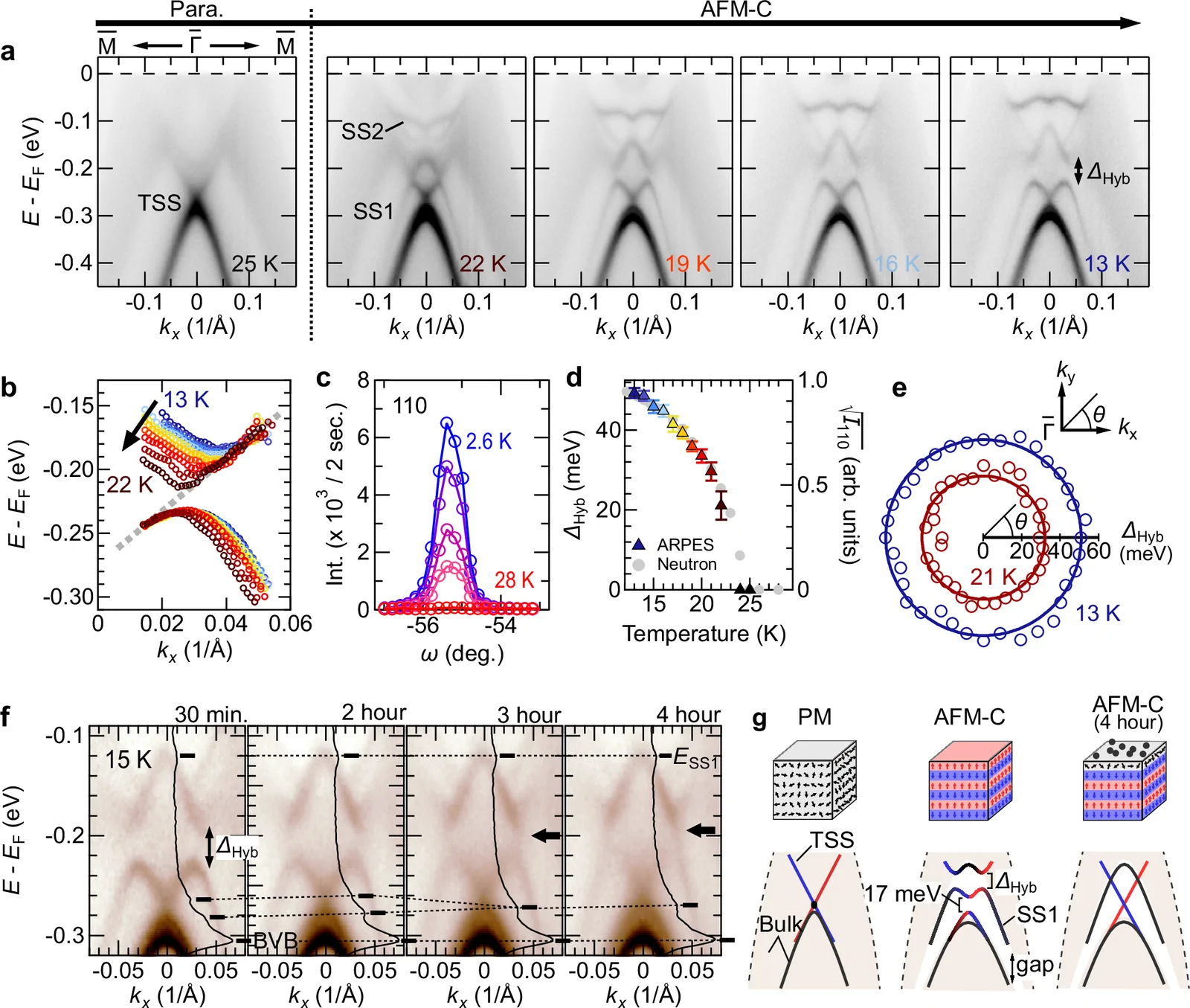
Evolution of the hybridization gap and Dirac cone with temperature and surface contamination. **a** Temperature evolution of ARPES images from 25 K (paramagnetic phase) to 13 K (AFM-C domain), showing the emergence of surface states (SS1 and SS2) and the development of the hybridization gap (*Δ*_Hyb_). **b** Temperature dependence of the Dirac cone and SS1, determined from the EDC peak positions. For the EDC fittings, we employed a Gaussian function combined with a linear background. No constraints were imposed on the fitting parameters. The gray dotted line traces the TSS in the PM phase. **c** Temperature dependence of the 110 magnetic reflection peak measured by neutron scattering. The horizontal axis *ω* denotes the sample rotation angle. **d** Comparison of the temperature dependence of the hybridization gap observed by ARPES (*Δ*_Hyb_, left) and the square root of the integrated intensity of the 110 magnetic Bragg reflection ($\sqrt{{I}_{110}}$, right). *Δ*_Hyb_ is defined as the energy difference between the top and bottom of the parabolic bands in (**b**). **e** Angular dependence of *Δ*_Hyb_ measured at 21 K and 13 K. The angle *θ* is defined from the $\bar{\Gamma }$--$\bar{{{{{\rm{M}}}}}}$ direction in the (*k*_*x*_, *k*_*y*_) plane, as illustrated in the inset. **f** ARPES images measured at 15 K at different time intervals after cleavage, showing the degradation of the Dirac and hybridization gaps due to surface contamination. EDCs at $\bar{\Gamma }$ are overlaid. **g** Schematic illustration of the electronic structure in the paramagnetic phase, in the AFM-C domain immediately after cleavage, and in the AFM-C domain after 4 h, highlighting the evolution of the surface Dirac gap (17 meV), hybridization gap (*Δ*_Hyb_), and surface state SS1.

Figure 5a exhibits the temperature evolution of bands in a wider energy window for Fig. 3d. Two surface states (SS1 and SS2), which are absent in the PM phase, abruptly emerge just below *T*_N_. As the temperature decreases, both the surface states shift significantly upward in energy, while the energy position of the Dirac cone remains unchanged. In particular, SS1 with a hole-like dispersion intersects the upper Dirac cone, and the band hybridization occurs at these intersections, leading to the formation of the M-shaped dispersion. The magnitude of the hybridization gap (*Δ*_Hyb_) increases as the temperature decreases. The gap opens isotropically, as shown in Fig. 5d, which presents the momentum-resolved gap distribution in the two-dimensional (*k*_*x*_, *k*_*y*_) plane at 21 K and 13 K. The complete temperature-dependent dataset from 25 K to 13 K in 1 K steps is provided as Supplementary Movie 1.

The band shift with temperature and the associated evolution of the hybridization gap are shown in Fig. 5b, which plots the band dispersions determined around the intersection between SS1 and the surface Dirac cone. To establish a direct link between the hybridization gap and the magnitude of the magnetic order, we additionally performed neutron scattering measurements. In particular, we measured the temperature dependence of the integrated intensity of the 110 magnetic Bragg reflection *I*_110_, whose square root $\sqrt{{I}_{110}}$ is proportional to the magnetic moment of Nd (Fig. 5c). As demonstrated in Fig. 5d, we find excellent agreement between the temperature evolutions of $\sqrt{{I}_{110}}$ (reflecting the magnetic order parameter) and the experimentally observed hybridization gap *Δ*_Hyb_. This provides compelling evidence that the gap opening originates from band hybridization induced by the exchange interaction associated with the magnetic order. Neutron scattering is a bulk-sensitive probe. Therefore, the observation of a hybridization gap between the surface bands indicates that the surface magnetism evolves in tandem with the bulk magnetic order, at least on a freshly cleaved surface. This result also suggests that the hybridization gap provides a sensitive probe of the surface magnetism.

The temperature evolution of surface states below *T*_N_—specifically, the emergence and energy shift of SS1 and its hybridization with the topological Dirac cone—can be understood as follows. In NdBi, AFM ordering folds the bulk bands, and a bulk gap opens at the magnetic zone boundary where the original and folded bulk bands intersect and mutually hybridize via the exchange interaction. In AFM-C, the bulk band folding occurs along the *k*_*z*_ direction, thereby opening the corresponding bulk gap around $\bar{\Gamma }$, which overlaps the original bulk gap in the PM phase. In general, surface states tend to appear inside bulk band gaps. This gap increases as temperature decreases, reflecting the growth of the magnetic moment and the concomitant enhancement of the exchange interaction. Likewise, SS1 and SS2 of NdBi emerge within this bulk gap that opens below *T*_N_. As the gap widens, SS1 shifts upward in energy. Concurrently, SS1 intersects the surface Dirac cone, thus a hybridization gap *Δ*_Hyb_ of these surface bands opens to form the M-shape dispersion. This hybridization arises from exchange interactions driven by surface magnetism, which increases as the bulk magnetism intensifies at lower temperatures. The emergence of the M-shaped surface band in the AFM phase is reproduced by our DFT calculations (Supplementary Note 6).

Our DFT calculations reveal a pronounced reconstruction of the bulk bands associated with the AFM phase transition (Supplementary Note 6), leading to a substantial enlargement of the bulk band gap. Importantly, this provides a natural explanation for the sizable gap of ~125 meV reported for the AFM-C domain in Ref. ^30^. A similarly large gap is observed in our synchrotron-ARPES measurements, in which the spectra are dominated by bulk contributions due to matrix-element effects, thereby demonstrating that such large gaps originate from bulk bands rather than from topological surface states.

A large Dirac gap (~80 meV) was also reported at $\bar{{{{{\rm{M}}}}}}$ in AFM-A domain^30^, despite the theoretical expectation that the Dirac point remains gapless due to symmetry protection. According to our DFT calculations, this apparent inconsistency can be consistently resolved by recognizing that the reported gap does not reflect an intrinsic surface Dirac gap, but instead arises from bulk-band contributions (Supplementary Note 7).

Since the early days of topological insulator research, surface contamination has been widely employed to probe perturbative effects on TSS^48–54^. This approach is particularly important for AFM-TIs, as studies of the MnBi_2*n*_Te_3*n*+1_ family suggest that surface contamination may be a key factor underlying the conflicting reports on the presence or absence of a surface Dirac gap. In the case of NdBi, this method is expected to provide deeper insights, as the hybridization gap can serve as an indicator of surface magnetism. The relationship between the Dirac gap and surface magnetism can therefore be identified through simultaneous observation of the hybridization gap.

In our study, we exploited natural surface contamination. We found a drastic change in both the hybridization and Dirac gaps even under an ultra-high vacuum of ~3 × 10^−11^ torr in the measurement chamber. Figure 5f displays the time evolution of the ARPES image for NdBi in the energy range capturing both the hybridization gap and the Dirac gap. Over time, the spectral quality, as evaluated by peak width, remained almost constant, indicating that surface degradation is not severe during the four-hour measurement. We found that the energy position of SS1 (*E*_SS1_) remained unchanged over time. However, the *Δ*_Hyb_ vanished four hours after the sample was cleaved (black arrows in Fig. 5f). This is attributed to the loss of surface magnetism. Our results suggest that the surface magnetic order is more fragile than the surface electronic structure and can be suppressed even when the band dispersion remains well-defined, with the spectral sharpness and hence the surface quality, nearly unchanged.

Very intriguingly, we found that the Dirac gap, clearly observed on a freshly cleaved surface, disappeared after three hours. This is evident in the EDC spectra at $\bar{\Gamma }$ (Fig. 5f): the double-peak structure associated with the gapped Dirac cone merges into a single peak after four hours, consistent with the Dirac gap closure. We emphasize again that the spectral peak width remained nearly constant throughout these measurements. This rules out surface-degradation-induced spectral broadening as the cause and indicates that these gap closures are intrinsic. The Dirac gap closure is coincident with the hybridization gap closure, thus validating that the Dirac gap originates from surface magnetism.

The time-dependent evolution of the band structure is schematically illustrated in Fig. 5g. The surface Dirac cone originates from the bulk band topology, as in the PM phase (left panel). The bulk magnetic order (or bulk exchange interactions) generates SS1, which intersects with the surface Dirac cone. On a freshly cleaved surface, the surface magnetic order aligns with the bulk magnetic order. The associated surface exchange interactions give rise to two types of gaps (middle panel): (i) a hybridization gap between the surface Dirac cone and SS1, resulting in an M-shaped surface band, and (ii) the Dirac gap, which opens due to time-reversal symmetry breaking induced by surface magnetism. Over time, residual gas gradually accumulates on the crystal surface. The surface Dirac cone and SS1 remain present since the surface contamination (right panel) does not affect either the bulk band topology or the bulk magnetism. Nonetheless, the magnetic order is lost at the surface, as supported by the disappearance of the hybridization gap *Δ*_Hyb_, and the Dirac gap closes.

Our DFT calculations reveal that surface states in the AFM-C domain are more localized on the crystal surface than those in the PM phase (Supplementary Note 8). While the decay length of the TSS into the bulk is approximately 17 nm in the PM state, it is reduced to 2–4 nm in the AFM-C domain. This reduction occurs because a large bulk gap, opened by bulk magnetism, significantly decouples the TSS from the bulk states in the AFM phase. When the TSS becomes more localized at the surface, it experiences the surface magnetism more strongly. Therefore, forming the gapped Dirac cone in NdBi requires both bulk magnetism, which decouples the TSS from the bulk, and surface magnetism, which couples directly to the localized TSS. Our experimental data in Fig. 4 indeed demonstrate that the Dirac gap is highly sensitive to the presence or absence of surface magnetic order, opening or closing accordingly.

## Discussion

The observed exchange gap in the Dirac cone of NdBi (17 ± 2 meV) is in excellent agreement with our DFT calculations (12 meV). Importantly, examination of the hybridization gap *Δ*_Hyb_ confirmed that this close match between theory and experiments occurs only when the surface magnetism is ideally aligned in the out-of-plane direction. Notably, the intrinsic gap we identified in NdBi is significantly larger than the vanishingly small gap observed in MnBi_2*n*_Te_3*n*+1_^21–25^. These results will provide an important clue toward the ultimate resolution of a central puzzle in AFM-TI research: the theory-reality mismatch.

In MnBi2Te4, a relatively small gap (<3 meV) limits the observation of the quantum anomalous Hall effect (QAHE) to ultra-low temperatures (~1.4 K)^13^. In contrast, the substantially larger gap observed in NdBi (17 ± 2 meV) suggests the possibility of realizing QAHE at elevated temperatures on the order of 10 K. Although further materials optimization will be required, this result points to a promising route toward higher-temperature QAHE.

We further note that thin-film fabrication offers a promising approach toward device realization. Quantum confinement in thin films is expected to drive a semimetal-to-semiconductor transition while preserving both magnetism and band topology^55,56^. In addition, electrostatic gating could enable tunability of the chemical potential and enhance the effective gap, offering a viable strategy to optimize QAHE conditions.

Finally, we point out that previous studies on magnetic topological insulators in the MnBi_2*n*_Te_3*n*+1_ family treat magnetism as a perturbation that opens a Dirac gap to an effective Hamiltonian that describes nonmagnetic states^18,24,57^. In contrast, our study reveals that magnetism in NdBi intricately intertwines with both bulk and surface states. Such magnetism is beyond the scope of a simple perturbative framework for understanding the magnetic topological states. The need to go beyond the perturbative regime arises from the strong interplay between localized 4*f* electrons and itinerant carriers—a qualitatively different situation from that in MnBi_2*n*_Te_3*n*+1_, where magnetic properties are perturbatively induced by magnetic layers composed of Mn atoms with 3*d* orbitals that are located in proximity to topological Bi_2_Te_3_ layers. These findings for the second-generation AFM-TI (NdBi) mark a significant step beyond the first-generation AFM-TI (MnBi_2*n*_Te_3*n*+1_) and provide valuable guidance for the future design and discovery of antiferromagnetic topological insulators among a wide variety of magnets.

## Methods

### Calculations

First-principles calculations were performed using the WIEN2k software^58,59^. We used the modified Becke–Johnson (BJ) potential^60^. The maximum modulus for the reciprocal lattice vectors ${K}_{\max }$ was chosen so that $R{K}_{\max }=8$ where the muffin-tin radius *R* for Nd and Bi atoms was 2.5 Bohr. We used a 19 × 19 × 19 ***k***-mesh with the inclusion of the spin-orbit coupling. The experimental lattice constant *a* = 6.41 Å was taken from Ref. ^61^.

For the paramagnetic phase, we adopt the open-core treatment for the Nd-*f* orbitals. For the spin-polarized phases, we adopt the DFT + *U* method^62,63^ with *U* = 6.3 eV and *J* = 0.7 eV for the Nd-*f* orbitals as adopted in the previous study^45^. We constructed the maximally localized Wannier functions^64,65^ for the Nd-*d* and Bi-*p* orbitals from the calculated band structure using the Wannier90 software^66^. The semi-infinite-slab tight-binding models constructed from these Wannier functions were used for calculating the surface spectra in the way described in Ref. ^67^.

### Single crystal growth

NdBi single crystals were grown by the Bridgman method with a sealed tungsten crucible and a high-frequency induction furnace. High-purity Nd (5N) and Bi (6N) metals with the respective composition ratios were used as starting materials. The obtained samples were characterized by X-ray diffraction patterns.

### ARPES measurements

All the high-resolution laser-ARPES measurements were performed at the Institute for Solid State Physics, the University of Tokyo, using two different setups with 6.994 eV lasers^68,69^. The domain-selective measurements (Fig. 1) and the observation of the Dirac cone (Fig. 3) were performed using an ARPES system equipped with a ScientaOmicron DA30L spectrometer. Spin-resolved ARPES (SARPES) measurements were conducted with VLEED-type spin-detectors installed on the ScientaOmicron DA30L spectrometer. We used an effective Sherman function of *S*_*eff*_ = 0.27. The VLEED-type spin detector operates by periodically switching the target magnetization during data acquisition, which effectively removes instrumental asymmetry. The energy (angular) resolution was set to 2.5 meV (0.1^∘^) and 21 meV (1.4^∘^) for ARPES and SARPES, respectively.

To investigate the temperature dependence of spectra in detail, we employed another ARPES system equipped with a Scienta R4000 analyzer. The excellent temperature tunability in the sample manipulator enabled temperature scans in fine steps of 1 K while maintaining the deviation within ±0.1 K^42^. The scans were first performed from lower to higher temperatures, followed by another temperature scan from higher to lower temperatures to ensure data reliability.

For temperature-dependent measurements, the samples were cleaved at the lowest temperature (13 K), whereas for other measurements, cleavage was performed at the measurement temperature. The beam spot size was approximately 50 μm, which is significantly smaller than the typical magnetic domain size (>200 μm, Supplementary Note 2), ensuring that the measurements predominantly probe a single magnetic domain. In addition, a total of 38 samples were cleaved and measured for ARPES. Although the data quality varies from sample to sample, the main results presented in the manuscript were found to be highly reproducible. We note that NdBi has a rock-salt crystal structure, for which a single crystallographic termination is expected upon cleavage.

The synchrotron-ARPES experiments with tunable low photon energies were performed at the BL7U beamline of UVSOR, equipped with an MBS A-1 analyzer. The energy resolution was better than 15 meV, and the angular resolution was 0.3^∘^. The synchrotron data (Figs. 4 and Supplementary S5) exhibit a slight distortion in the band shape due to a minor imperfection in the analyzer lens setting. While this affects the precise determination of the band dispersion and prevents a fair comparison across different photon energies, it does not affect the key observation that the surface-state intensity is strongly suppressed at 10 eV due to matrix element effects.

In all these ARPES experiments, we used the linear polarization with the electric field aligned in the incident plane. The crystals were cleaved in situ at the measurement temperatures. The base pressure during the ARPES measurements was better than 1 × 10^−8^ Pa.

### Neutron scattering experiment

Neutron scattering experiments were conducted using the POlarized Neutron Triple-Axis triple-axis spectrometer PONTA installed at the 5G beamhole in the Japan Research Reactor 3 (JRR-3) of Japan Atomic Energy Agency^70^. The spectrometer was operated in a two-axis diffraction mode. An incident neutron beam with the energy of 34.05 meV was obtained using a pyrolytic graphite monochromator. A single crystal sample with a volume of 22.52 mm^3^ was oriented in the (H, K, 0) horizontal scattering plane and loaded in a clamp-type uniaxial-stress cell^71,72^. A uniaxial stress of 25 MPa was applied onto the (001) surfaces of the sample, so that the magnetic domains with the magnetic moments parallel to the [001] direction were selected at low temperatures. The cell was attached to the cold head of a closed-cycle ^4^He refrigerator and cooled to the lowest temperature of 2.3 K. Further experimental details are provided in Supplementary Note 4.

## Supplementary information

Supplementary Information

Peer Review File

Description of Additional Supplementary Files

Supplementary Movie 1

## Data Availability

The data that support the findings of this study are available in figshare, “https://doi.org/10.6084/m9.figshare.32920193”.
