# Supplementary material for: Direct observation of a spin-polarized surface Dirac gap in the antiferromagnetic topological insulator NdBi
Source: Nat Commun. 2026 Sep 15;17:10841. doi: 10.1038/s41467-026-77571-4 (PMC13672286; doi:10.1038/s41467-026-77571-4)
Supplement: Supplementary file 1 [file 41467_2026_77571_MOESM1_ESM.pdf]

Supplementary Information for "Direct observation of a spin-polarized surface Dirac gap in the antiferromagnetic topological insulator NdBi"

Yuto Fukushima,[1] Hiroyuki S. Suzuki,[1] Masayuki Ochi,[2,3] Takuma Nishida,[1] Toshiya Ikenobe,[1] Hiraku Saito,[1] Yuto Kinoshita,[1] Kaishu Kawaguchi,[1,4] Hiroaki Tanaka,[1] Ayumi Harasawa,[1] Takushi Iimori,[1] Koichiro Yaji,[5,6] Masashi Tokunaga,[1] Taro Nakajima,[1,7,8] Kiyohisa Tanaka,[9,10] Takahiro Morimoto,[11,12] Ryo Mori,[1,4] and Takeshi Kondo[1,13,*]

[1] *Institute for Solid State Physics, The University of Tokyo, Kashiwa, Chiba 277-8581, Japan*

[2] *Department of Physics, The University of Osaka, Toyonaka, Osaka 560-0043, Japan.*

[3] *Forefront Research Center, The University of Osaka, Toyonaka, Osaka 560-0043, Japan.*

[4] *Maryland Quantum Materials Center, Department of Physics, University of Maryland, College Park, MD 20742, USA*

[5] *Center for Basic Research on Materials, National Institute for Materials Science (NIMS), 3-13 Sakura, Tsukuba, 305-0003, Ibaraki, Japan*

[6] *Unprecedented-Scale Data Analytics Center, Tohoku University, 468-1 Aoba, Aramaki-Aza, Aoba-Ku, Sendai, 980-8578, Japan*

[7] *Institute of Materials Structure Science, High Energy Accelerator Research Organization, Tsukuba, Ibaraki 305-0801, Japan*

[8] *RIKEN Center for Emergent Matter Science, Wako 351-0198, Japan*

[9] *SOKENDAI (The Graduate University for Advanced Studies), Okazaki 444-8585, Japan*

[10] *UVSOR Synchrotron Facility, Institute for Molecular Science, Okazaki 444-8585, Japan*

[11] *Department of Applied Physics, The University of Tokyo, Hongo, Tokyo, 113-8656, Japan*

[12] *Department of Physics, Kyoto University, Kyoto, 606-8502, Japan*

[13] *Trans-scale Quantum Science Institute, The University of Tokyo, Tokyo 113-0033, Japan*

(Dated: July 5, 2026)

Supplementary Note 1: Sample characteristics

The sample quality was confirmed by measurements of magnetic susceptibility (Fig. S1a) and electrical resistivity (Fig. S1b). Magnetization was measured in a field of 1 kOe. Both measurements exhibit an anomaly at $T_{\mathrm{N}} = 24$ K. The residual resistivity ratio (RRR), estimated as $\rho(300\,\mathrm{K})/\rho(4.2\,\mathrm{K}) = 292$, indicates excellent crystal quality.

Supplementary Note 2: Optical microscopy image

The type-A antiferromagnetic order in a cubic lattice makes three types of magnetic domains with the ordered moments along [100], [010], and [001]. Following the notation in the main text, we refer to these magnetic domains (A, B, and C), respectively. To visualize the multi-domain state in our high-quality sample, we employed polarized optical microscopy, a technique previously applied to this [1, 2] and its sister compound, CeSb [3, 4]. The measurement was performed at ISSP, the University of Tokyo. The details of the experimental setup are described in Ref. [5]. The sample was cleaved in the atmosphere and installed immediately into the vacuum chamber.

The microscope image observed in the Antiferromagnetic (AFM) phase (Fig. S2a, 9 K) shows additional contrast compared to the image observed in the paramagnetic (PM) phase (Fig. S2b, 30 K). The temperature dependence is well visualized in the differential intensity map $I_{\mathrm{diff}} = (I_{9\mathrm{K}} - I_{30\mathrm{K}})\,/\,2(I_{9\mathrm{K}} + I_{30\mathrm{K}}) \times 100$ (Fig. S2c). The change in intensities between before and after the magnetic transition is primarily due to the optical birefringence under the tetragonal magnetic symmetry in the AFM phase. The magnified differential intensity map indicated by the rectangle in Fig. S2c is shown in Fig. S2d. It clearly shows three domain regions represented by red, blue, and white areas. Domain sizes are basically larger than our laser spot ($\sim 50\ \mu$m) installed in our high-resolution laser-ARPES, enabling the domain-selective ARPES measurements.

For better visualization, the color scale has an offset of 0.12%, determined from a distribution of the differential intensities in Fig. S2d (Fig. S2e). It shows three peaks attributed to the three magnetic domains. We chose the middle peak as the offset for the color scales used in Fig. S2c and d.

Supplementary Note 3: ARPES results for all three magnetic Domains

While the main text presents ARPES results only for Domains A and C, here we also include data for Domain B for comparison. Figure S3a shows the Fermi surface and band dispersion maps measured in the paramagnetic phase (at $T = 44$ K, Fig. S3a). Upon cooling

* kondo1215@issp.u-tokyo.ac.jp

the sample to $T = 13$ K (below T_{N}), these features drastically change due to the magnetic phase transition (Fig. S3b).

Although a direct real-space domain map is not available in the present ARPES measurements, and the precise domain population has not been determined, we performed spatially resolved ARPES measurements by scanning the beam across the cleaved sample surface. This approach allows us to probe different regions of the sample and distinguish magnetic domains based on their Fermi surface characteristics.

We obtained ARPES data from all three domains on the same cleavage plane, as displayed in Fig. S3b. The magnetic domains were identified based on distinct symmetry fingerprints of the Fermi surface: Domains A and B exhibit twofold symmetry, whereas Domain C displays fourfold symmetry. The domain assignment was performed by directly matching the measured Fermi surface patterns to these symmetry characteristics, providing an objective and unambiguous classification of the domains.

Supplementary Note 4: Characterization of magnetic domains under uniaxial stress via neutron scattering

For the neutron scattering measurements, the single crystal sample was mounted in the uniaxial-stress cell with the (H,K,0) horizontal scattering plane (Figs. S4a). The (001) surface of the sample was polished to minimize possible inhomogeneity of the applied stress. The cell was clamped under a pressure of 25 MPa at room temperature before being installed into the vacuum chamber of the cryostat.

Fundamental neutron scattering results are presented in Figs. S4b–e. The 400 nuclear Bragg peak shown in Fig. S4b serves as a reference; its intensity remains constant across the antiferromagnetic (AFM) transition. As shown in Fig. S4c, we observed a strong magnetic Bragg peak at the reciprocal lattice point of (1,1,0), where the nuclear Bragg reflections is forbidden, at low temperatures. This reflection corresponds to the AFM-C domain, and disappeared in the paramagnetic phase at 30 K, demonstrating its magnetic origin.

To evaluate domain distribution under strain, we measured the 210 and 120 reflections corresponding to the AFM-A and AFM-B domains, respectively (Figs. S4d, e). Besides the magnetic peaks (black arrows), these spectra include temperature-independent signals from the Al cell. We measured integrated intensities of the 110, 210 and 120 reflections by $\theta - 2\theta$ scans at low temperatures, and also repeated the same scans at high temperatures to subtract background signals. Taking into account the magnetic form factor of Nd^{3+} and Lorentz factor, we estimated the volume fractions of the three magnetic domains from the integrated intensities, revealing that the AFM-C population exceeds 98 %. This demonstrates that uniaxial strain selectively stabilizes a single magnetic domain.

Supplementary Note 5: Suppression of surface state spectra at higher photon energies.

In the main text, we used a 7 eV laser as the photon source for our ARPES measurements and revealed the surface state that emerges within the bulk band gap. Here, we demonstrate that the surface-state signals weaken at higher photon energies, such as 10 eV, and the ARPES spectra eventually become dominated by bulk band signals. For this purpose, we used synchrotron radiation tunable from 7 eV to higher photon energies at the BL07 of the UVSOR Synchrotron Facility.

Figure S5a shows ARPES band dispersions along the $\bar{\Gamma}$-$\bar{\mathrm{M}}$ direction, obtained from the antiferromagnetic domain C (AFM-C). The left-to-right panel corresponds to data measured at photon energies from 7 eV to 10 eV. Below 9 eV, the two surface states (SS1 and SS2) are clearly observed. However, they almost vanish at 10 eV, most likely due to matrix element effects in photoemission. This is more clearly demonstrated in Fig. S5b by extracting the EDCs at $k_x = 0$ Å^{-1}. The spectral shape varies with increasing photon energy, and at 10 eV, the in-gap surface state is not distinguishable from the bulk spectra. In Fig. S5c, the EDC at 10 eV was fitted with two Gaussian curves, yielding a gap magnitude of 83 meV. This is much larger than the Dirac gap (17 meV) determined from the data measured with a 7 eV laser ARPES in the main text. The large gap (83 meV) does not originate from the Dirac surface state, but rather from the bulk band. This discrepancy, coming from the photon energies used, may be a primary reason that the energy gap claimed as the one for the Dirac point varies across different research groups. Note that a similar situation has been reported for the $MnBi_2Te_4$ system [6–8], in which photon energies lower than $\sim$18 eV are required to detect the surface Dirac state which emerges within the bulk band gap.

Supplementary Note 6: Substantial enhancement of the bulk band gap under antiferromagnetism

Here, we use band calculations around $\bar{\Gamma}$ to support the argument made in Supplementary Note 5 that the large gap (83 meV) observed in the antiferromagnetic phase of NdBi originates from the bulk bands. In particular, we demonstrate that the calculated bulk bands with a Dirac-like shape significantly enhance the bulk gap in the antiferromagnetic phase, and the spectral gap observed by ARPES with a finite energy resolution could be as large as $\sim$ 100 meV. This is nearly one order of magnitude larger than the gap (12 meV) that opens at the Dirac point of the topological surface state (TSS) in the surface calculations.

As a first step, we reproduce in Fig. S6a,b the band calculations for the paramagnetic phase presented in the main Fig. 2. Figure S6a shows the "bulk calculations" that include

only the bulk bands, whereas Figure S6b shows the “surface calculations” that include both the bulk and surface bands. The bulk bands (integrated over the entire k_z) resemble a Dirac dispersion, characterized by sharp spectral features. A band gap opens between the bulk bands of the heavy hole pocket and the electron pocket, as indicated by an arrow in Fig. S6a. Within this bulk band gap, the TSS emerges, as revealed by the surface-inclusive calculation in Fig. S6b.

We next performed calculations for the antiferromagnetic (AFM) phase. Here, we selected the Domain-C (AFM-C) configuration and obtained results for both the bulk bands only (Fig. S6c) and the bulk plus surface states (Fig. S6d). This comparison clearly distinguishes bulk from surface bands. In AFM-C, the AFM order causes the heavy-hole and electron pockets to hold along the k_z direction, leading to additional band hybridizations and the opening of two band gaps (arrows in Fig. S6c). Within these newly formed gaps, two surface states (SS1 and SS2) appear, as shown in Fig. S6d. The lower-energy gap overlaps with the original bulk gap in the PM phase (Fig. S6a), within which a surface Dirac cone resides. This gap is enhanced by the exchange interaction of antiferromagnetism. Inside the enlarged bulk gap, the surface band (SS1) forms an M-shaped dispersion (Fig. S6d). As discussed in the main text, time-reversal symmetry breaking on the crystal surface opens an energy gap at the Dirac point of this M-shaped band. Although the gap is relatively small (12 meV), it agrees well with our ARPES results (17 meV).

As discussed in Supplementary Note 5, the intensity of surface state signals can become weak at high photon energies due to matrix element effects. Such data could be highly misleading, since the bulk band retains its Dirac-like shape, and the bulk band gap becomes enlarged in the antiferromagnetic phase. To simulate this situation, we extracted energy distribution curves (EDCs) at $\bar{\Gamma}$ from the bulk calculations without surface states (Fig. S6e) for both the paramagnetic phase (black curve) and the antiferromagnetic phase (red curve). To make the simulation more realistic, we introduced Gaussian broadening with an assumed energy resolution of 20 meV to the EDC in Fig. S6(f). The resulting EDCs indicate that the bulk band gap enlarged under antiferromagnetism reaches as $\sim$100 meV (red arrow).

These arguments indicate that the estimation of the intrinsic topological Dirac gap requires carefully optimized experimental conditions: proper low photon energies and high resolution. Our experimental setting matches that condition. Moreover, our spin-resolved ARPES measurements are essential for distinguishing the bulk and surface states, which become the key to achieving our research goals. We emphasize that our experimental data are the first that decisively identifies the intrinsic topological Dirac gap of NdBi experimentally.

Supplementary Note 7: Electronic band evolution at the $\bar{\mathrm{M}}$ point across the

antiferromagnetic phase transition investigated by DFT calculations

In the main text, we focus on the band structure around the $\bar{\Gamma}$ point. Here, we investigate the band structures around the $\bar{\mathrm{M}}$, in particular, their evolution across the antiferromagnetic phase transition, using our DFT calculations (Fig. S7). The reliability of these calculations is supported by their excellent agreement with our ARPES results around the $\bar{\Gamma}$ point.

Before discussing this momentum region in detail, we note that the electronic bands around the $\bar{\mathrm{M}}$ do not directly reflect the underlying nontrivial Z_2 topology.

In NdBi, a single Dirac cone is located at the $\bar{\Gamma}$ point, where it represents an odd number of topological surface states (TSSs) required by the nontrivial Z_2 invariant. While TSSs are also observed at the $\bar{\mathrm{M}}$ point, these arise from the projection of multiple bulk band inversions and give rise to an even number of Dirac cones. Such states appearing at the $\bar{\mathrm{M}}$ point may not be topologically protected; therefore, Dirac cones at the $\bar{\mathrm{M}}$ are not suitable for investigating the interaction between magnetism and the nontrivial Z_2 topology. This possibility is also mentioned in [1].

Measurements at the $\bar{\Gamma}$ point, where the relevant TSS resides, are experimentally challenging due to k_z broadening, which leads to overlap with projected bulk bands. Nevertheless, direct observation of a Dirac gap for the topological Dirac cone at the $\bar{\Gamma}$ point remains indispensable for identifying the antiferromagnetic topological phase in this system.

Because of this intrinsic difficulty, the identification cannot rely solely on energy-momentum intensity maps and requires going beyond conventional ARPES approaches. This difficulty arises because bulk-derived Dirac-like bands and k_z broadening fundamentally obscure the surface contribution in conventional ARPES measurements.

In this work, we combine high-resolution 7 eV laser ARPES with spin-resolved measurements. Spin-resolved ARPES is essential to distinguish the spin-polarized topological surface state from bulk-derived bands. This allows a direct identification of the TSS. Importantly, the gap opening at the Dirac point of the spin-polarized surface band is in quantitative agreement with our DFT surface calculations, further supporting the intrinsic origin of the observed Dirac gap. Taken together, the consistency between spin-resolved ARPES and surface-state calculations provides a compelling and self-consistent identification of the topological Dirac gap.

Still, momentum space around the $\bar{\mathrm{M}}$ point, where surface and bulk states are more clearly separated, can be advantageous for evaluating the impact of magnetism on the opening of a Dirac gap in NdBi associated with time-reversal symmetry breaking. The 7 eV laser ARPES employed in this work does not access this momentum region due to its low photon energy. Nevertheless, our DFT calculations allow us to investigate the evolution of the band structure across the antiferromagnetic transition. Importantly, they identify the relation-

ship between bulk and surface bands and provide a consistent interpretation of previously reported large gaps [1], attributing them to bulk-band features rather than to the intrinsic TSS. In particular, we find that sharp dispersive features can arise from both surface and projected bulk bands, and their distinction cannot be made based solely on spectral sharpness. Furthermore, we note that a large gap has been reported even in symmetry-protected situations where the Dirac point is expected to remain gapless [1]. Within our framework, this apparent discrepancy can be naturally explained by bulk-band contributions rather than by the intrinsic gap opening of the TSS. Projected bulk bands can exhibit sharp dispersions and overlap with surface states, leading to an overestimation of the surface-state gap if not properly distinguished.

The above-mentioned mechanism is summarized in Fig. S7, where we present DFT calculations around the $\bar{\mathrm{M}}$ point above and below T_{N} (top and bottom panels, respectively) for both Domain-A and Domain-B (left and right panels, respectively).

Domain C:

Figures S7a1,a2 show the spectral intensity maps of bulk and surface calculations in the paramagnetic phase above T_{N}, while the corresponding results in the antiferromagnetic phase below T_{N} are shown in Figs. S7c1,c2. Upon entering the AF phase, the bulk gap becomes significantly enlarged.

In contrast, the intrinsic gap at the Dirac point of the TSS at the $\bar{\mathrm{M}}$ point remains much smaller, about 41 meV (Fig. S7b5). Notably, the energy separation between the nearly flat TSS branch and the bulk bands is approximately 160 meV (Fig. S7c4), which is comparable to the gap size reported as the TSS gap in Ref. [1].

This comparison indicates that the previously reported large gap can be attributed to the energetic separation between surface and bulk states, rather than to the intrinsic magnetic gap of the TSS. This result highlights the importance of reliably distinguishing surface and bulk contributions, as achieved here using spin-resolved ARPES.

Domain A:

Ref. [1] also reported a similarly large gap at the Dirac point of the TSS for Domain A, where the Dirac point is expected to remain symmetry-protected and gapless. In Ref. [1], the origin of this large gap was not identified. Within our framework, however, this observation can be consistently explained by bulk-band contributions rather than by an intrinsic gap opening of the TSS Dirac point. Supporting DFT calculations are presented in Fig. S7b.

The large gap reported in Ref. [1] is likely influenced by the photon energy used in the ARPES measurements ($h\nu = 60$ eV). In our experiments, we find that the spectral weight of the TSS decreases with increasing photon energy and becomes nearly undetectable above

∼10 eV, where bulk-band contributions dominate. Our results clearly demonstrate that the surface states are distinctly resolved only at low photon energies around 7 eV.

A similar photon-energy dependence has been reported in another antiferromagnetic topological insulator, $MnBi_2Te_4$ [6–8], where higher photon energies predominantly probe bulk states with a large gap in the antiferromagnetic phase, while lower photon energies are required to clearly resolve the surface bands.

Supplementary Note 8: Evolution of the penetration depth of the topological surface state into the bulk across the magnetic transition

The Dirac gap is formed by the breaking of the symmetry composed of translational and time-reversal symmetries (S-symmetry), namely, by ferromagnetic order at the outermost surface. Therefore, the localization of the TSS wavefunction is crucial for the Dirac gap opening. To evaluate the surface localization, here, we estimate the penetration depth of the surface states into the bulk.

For this purpose, we calculate the layer-dependent spectral weight of TSS in the paramagnetic phase (PM) and the antiferromagnetic phase, Domain-C (AFM-C). We particularly focused on the energy-momentum states labeled as α (for PM) and β, γ (for AFM-C) on the surface calculation maps in Fig. S8a and c, respectively. The calculated results are shown in Fig. S8b, d and e. The spectral weight significantly decreases inside the bulk in all states. To highlight the difference, we present the same data on a logarithmic scale. It is now clear that the α state in the PM phase has a relatively long decay length, whereas the β and γ states in the AFM-C phase show more rapid decay inside the bulk. In Fig. S8b, d and e, we perform an exponential fit to each dataset and estimate the penetration depth τ. The estimated values of τ are explicitly indicated in each panel. Interestingly, the penetration depth in the PM phase is relatively long (∼17 nm), whereas it gets much shorter (2 to 4 nm), reduced by a factor of ∼5-7, in the AFM-C phase. The significant reduction of τ across the magnetic transition originates from a qualitative change in the coupling between the surface and bulk states. In the PM phase, the TSS overlaps with the bulk bands projected onto the surface, indicated as spectral continuum in Fig. S8a. The consequent wavefunction mixing between TSS and bulk states allows the TSS to penetrate deep inside the bulk. In contrast, TSS in the AFM-C resides within the bulk band gap largely opened under antiferromagnetism, as demonstrated in Fig. S8c. This strongly suppresses wavefunction mixing between the surface and bulk states. This reduced coupling results in the surface states being more localized at the surface without penetrating into the bulk in the AFM phase.

To test the dependence on the U parameter, we performed another calculation for the antiferromagnetic phase, Domain-C using $U = 5.3$ eV in Fig. S8f (We used $U = 6.3$ eV in

the main manuscript). The calculated decay lengths of the upper and lower parts of TSS with this parameter are $4 \sim 6$ nm. This value confirms that the TSS in the AFM phase is more localized to the surface compared to the paramagnetic phase (~ 17 nm), regardless of the variation in U. Regarding the surface termination, there is structurally only one surface termination along the (001) cleavage plane due to the NaCl structure. While the magnetic orientation of the topmost layer can be reversed, this simply inverts the sign of the S_z spin texture and does not affect the decay length. Therefore, the surface termination effect is not present in this system.

Supplementary Note 9: Spin-resolved DFT calculations in the paramagnetic and antiferromagnetic phases

Here, we present spin-resolved DFT calculations, which are an effective means of distinguishing surface and bulk states, for both the paramagnetic and antiferromagnetic phases.

Figure S10a shows the surface spectral function (spin-integrated) for the PM phase. The TSS and bulk bands overlap in energy-momentum space, making them indistinguishable in spin-integrated spectra. This ambiguity, however, is resolved by considering spin polarization. Figure S10b presents the corresponding spin-resolved spectral function. The TSS appears at the edge of the bulk continuum and exhibits a clear spin polarization. Although the bulk states exhibit weak spin polarization due to surface-induced inversion symmetry breaking, their spin polarization is significantly weaker and, importantly, of opposite sign to that of the TSS.

We further performed spin-resolved calculations for the antiferromagnetic (AFM) phase (Figs. S10c and S10d). The results show that the sign of the spin polarization of the TSS remains unchanged across the AFM transition. In the AFM phase, the TSS is well separated from the bulk bands and can be clearly identified (see Figs. 3g and 3h in the main manuscript), providing a reliable benchmark for interpreting the experimental data. We find that the same spin polarization is observed in the PM phase, indicating that the feature assigned as the TSS in the PM phase originates from the same surface state. If the peaks were instead derived from bulk states, their spin polarization would exhibit the opposite sign according to our calculations; however, this is not observed. This directly rules out a bulk origin of the observed features.

The consistency of the spin polarization between the PM and AFM phases provides strong evidence that our 7 eV laser ARPES selectively probes the TSS even in the PM phase. In our measurements, the bulk contribution is strongly suppressed, likely reflecting a combination of intrinsically weaker bulk spectral weight and photoemission matrix element effects, which enhance the relative visibility of the surface state.

Supplementary Note 10: Quantitative analysis of the spin polarization of the TSSs observed by spin-resolved laser ARPES

We present a quantitative analysis of the spin polarization as a function of energy at $\pm k$ in Fig. S11. The error bars reflect the statistical uncertainty derived from counting statistics. The overall energy and momentum resolutions are described in the Methods section.

Supplementary Note 11: Determination of the topological Dirac gap size from laser-ARPES

In the main text, we report that the topological Dirac gap is 17 ± 2 meV. Here, we present a fitting analysis of the relevant spectrum to determine the gap size and its associated uncertainty.

Figure S9 shows the EDC at the $\bar{\Gamma}$ point of the AFM-C domain (Fig. 3d in the main manuscript), together with the fitting curve. We fit the EDC using a combination of three Gaussian functions with a linear background. This estimates the gap size to be 17 ± 2 meV. To assess reproducibility, we repeated the measurements on three independent sample cleaves and consistently obtained similar spectral features and gap magnitudes, which ensures the reliability of our results. The uncertainty was determined from the fitting procedure.

Supplementary Note 12: Critical scaling of the hybridization gap with temperature

Below the T_{N}, the TSS and the SS1 hybridize to form a gap, denoted as Δ_{Hyb} (Fig. S12a). The evolution of the hybridization gap is shown in Fig. S12b. We found that the extracted temperature dependence of Δ_{Hyb} is well described by a power-law fit $\Delta_{\mathrm{Hyb}} \propto (1 - T/T_{\mathrm{N}})^{\beta}$, with $\beta = 0.32(6)$ and $T_{\mathrm{N}} = 22.8(7)$ K. The obtained critical exponent β closely matches the theoretical magnetic order parameter for a 3D Ising model [9], whereas it deviates significantly from that of a 2D Ising model (indicated by the dotted line in Fig. S12b).

These results demonstrate that the surface magnetism evolves in tandem with the bulk magnetic order, at least on a freshly cleaved surface. For the fitting procedure, the surface T_{N} was treated as a free parameter. The slight deviation (~ 1.2 K) from the bulk T_{N} (24 K) is likely attributable to minor experimental factors such as a thermal gradient between the sample holder and the sample surface, calibration offset of the thermometer, or potentially a small intrinsic reduction of T_{N} at the surface. In any case, this small difference does not affect our main conclusions.

Supplementary Note 13: Quantitative analysis of the time evolution of the hy-

bridization gap

To quantify the time evolution of the hybridization gap, we analyzed the ratio of the spectral intensity within the gap to that of the surface state, as shown in Fig. S13e, where the horizontal axis represents the elapsed time after cleavage. The data show a monotonic increase in the in-gap intensity over time, quantitatively confirming progressive spectral filling that leads to gap closure after ∼4 hours. The uncertainties in the intensity ratio were estimated from the statistical variation of the integrated spectral weight and are included in Fig. S13e.

Supplementary Note 14: Temperature and pressure stability of the experimental setup

To confirm the temperature and pressure stability of our experiments, we conducted independent tests over a period of one hour. Fig. S14a demonstrates that the base pressure remains highly stable at $< 5 \times 10^{-11}$ Torr over more than one hour at low temperatures, representing the typical pressure history during our measurements. Regarding thermal stability, the temperature was controlled using a PID controller. As shown in Fig. S14b, the standard deviation over a 30-minute period was 6.8 mK, confirming negligible thermal drift within the experimental time window. Furthermore, Fig. S14c confirms that the temperature sweep rate of 1 K/min used in our experiments does not induce any pressure fluctuations.

[1] Honma, A. *et al.* Antiferromagnetic topological insulator with selectively gapped Dirac cones. *Nat. Commun.* **14**, 7396 (2023). URL `https://www.nature.com/articles/s41467-023-42782-6`.

[2] Yamamoto, R. *et al.* Spin splitting in the surface electronic structure of antiferromagnet NdBi. *Phys. Rev. Res.* **7** (2025). URL `http://dx.doi.org/10.1103/physrevresearch.7.l022005`.

[3] Kuroda, K. *et al.* Devil's staircase transition of the electronic structures in CeSb. *Nat. Commun.* **11**, 2888 (2020). URL `https://www.nature.com/articles/s41467-020-16707-6`.

[4] Arai, Y. *et al.* Multipole polaron in the devil's staircase of CeSb. *Nat. Mater.* **21**, 410–415 (2021). URL `http://dx.doi.org/10.1038/s41563-021-01188-9`.

[5] Katakura, I. *et al.* Development of high-speed polarizing imaging system for operation in high pulsed magnetic field. *Rev. Sci. Instrum.* **81**, 043701 (2010). URL `http://dx.doi.org/10.1063/1.3359954`.

[6] Chen, Y. J. *et al.* Topological electronic structure and its temperature evolution in antiferromagnetic topological insulator $MnBi_2Te_4$. *Phys. Rev. X.* **9** (2019). URL `https:`

//journals.aps.org/prx/abstract/10.1103/PhysRevX.9.041040.

[7] Chen, B. *et al.* Intrinsic magnetic topological insulator phases in the Sb doped $MnBi_2Te_4$ bulks and thin flakes. *Nature Communications* **10**, 4469 (2019). URL https://doi.org/10.1038/s41467-019-12485-y.

[8] Hao, Y.-J. *et al.* Gapless surface Dirac cone in antiferromagnetic topological insulator $MnBi_2Te_4$. *Phys. Rev. X.* **9** (2019). URL https://journals.aps.org/prx/abstract/10.1103/PhysRevX.9.041038.

[9] Guida, R. & Zinn-Justin, J. Critical exponents of the N-vector model. *Journal of Physics A* **31**, 8103–8121 (1998). URL https://iopscience.iop.org/article/10.1088/0305-4470/31/40/006/meta.

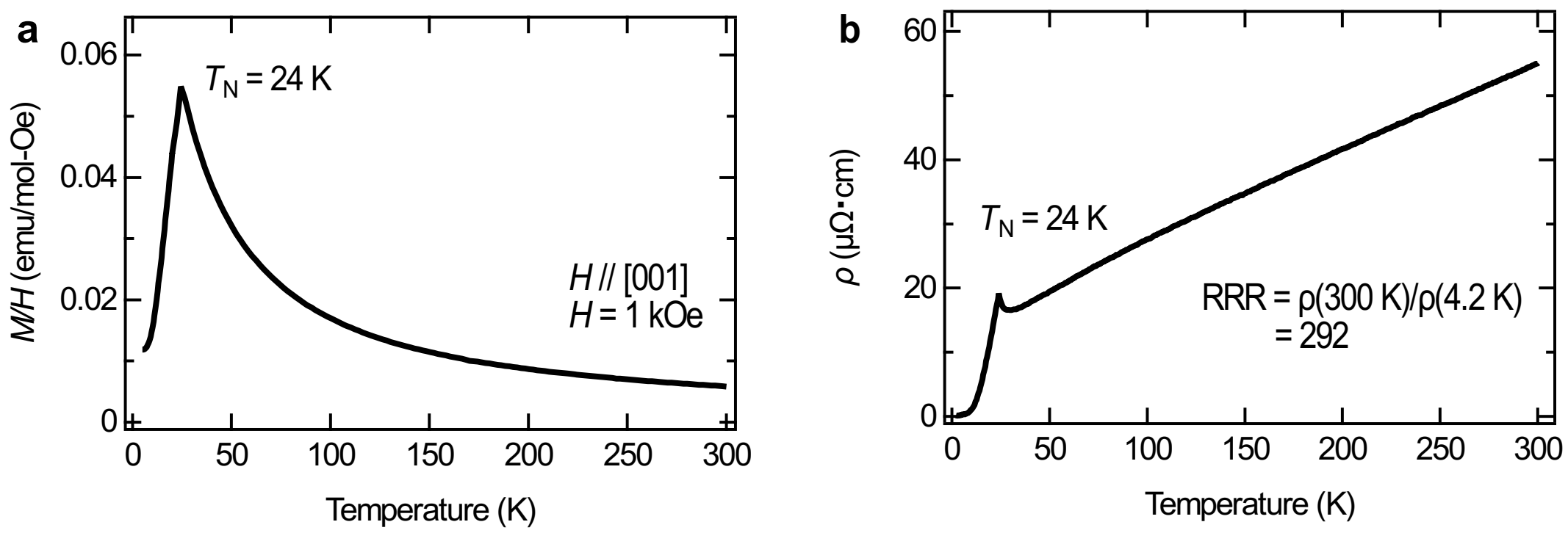

FIG. S1. **a**, Temperature dependence of the magnetization M/H of NdBi, measured with an applied field of 1 kOe along the [001] direction. **b**, Temperature dependence of the electrical resistivity ρ measured in zero field. The Néel temperature $T_{\mathrm{N}} = 24$ K is clearly observed in both panels. The residual resistivity ratio is estimated to be RRR $= \rho(300\text{ K})/\rho(4.2\text{ K}) = 292$, indicating high crystal quality.

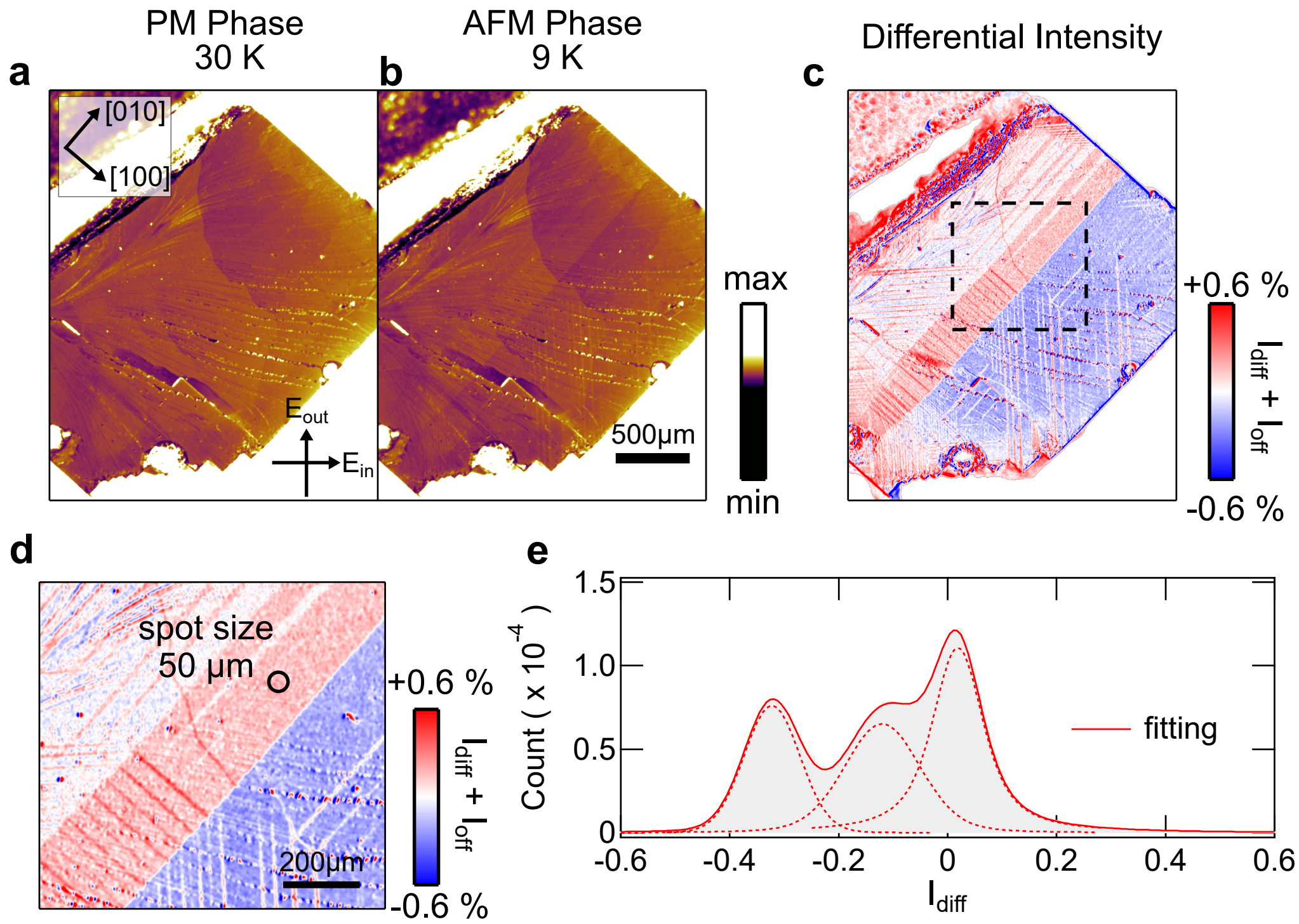

FIG. S2. Polarized optical microscope images taken at $T = 30$ K (paramagnetic phase: PM, **a**) and at $T = 9$ K (antiferromagnetic phase: AFM, **b**). We used the crossed Nicol configuration with the incident light polarized along the [110] direction to visualize the ordered magnetic domain. The differential intensity map $I_{\rm diff}$ obtained by subtracting **b** from **a** is presented in **c** using a color scale with a constant offset $I_{\rm off} = 0.12$. **d**,The magnified differential intensity map indicated by a rectangle in **c**. The spot size of our laser system, used for domain-selective ARPES measurements, is represented by an open circle. **e**, Distribution of differential intensities of **d**. The offset $I_{\rm off}$ for the color scale is determined from the middle peak of the fitting result.

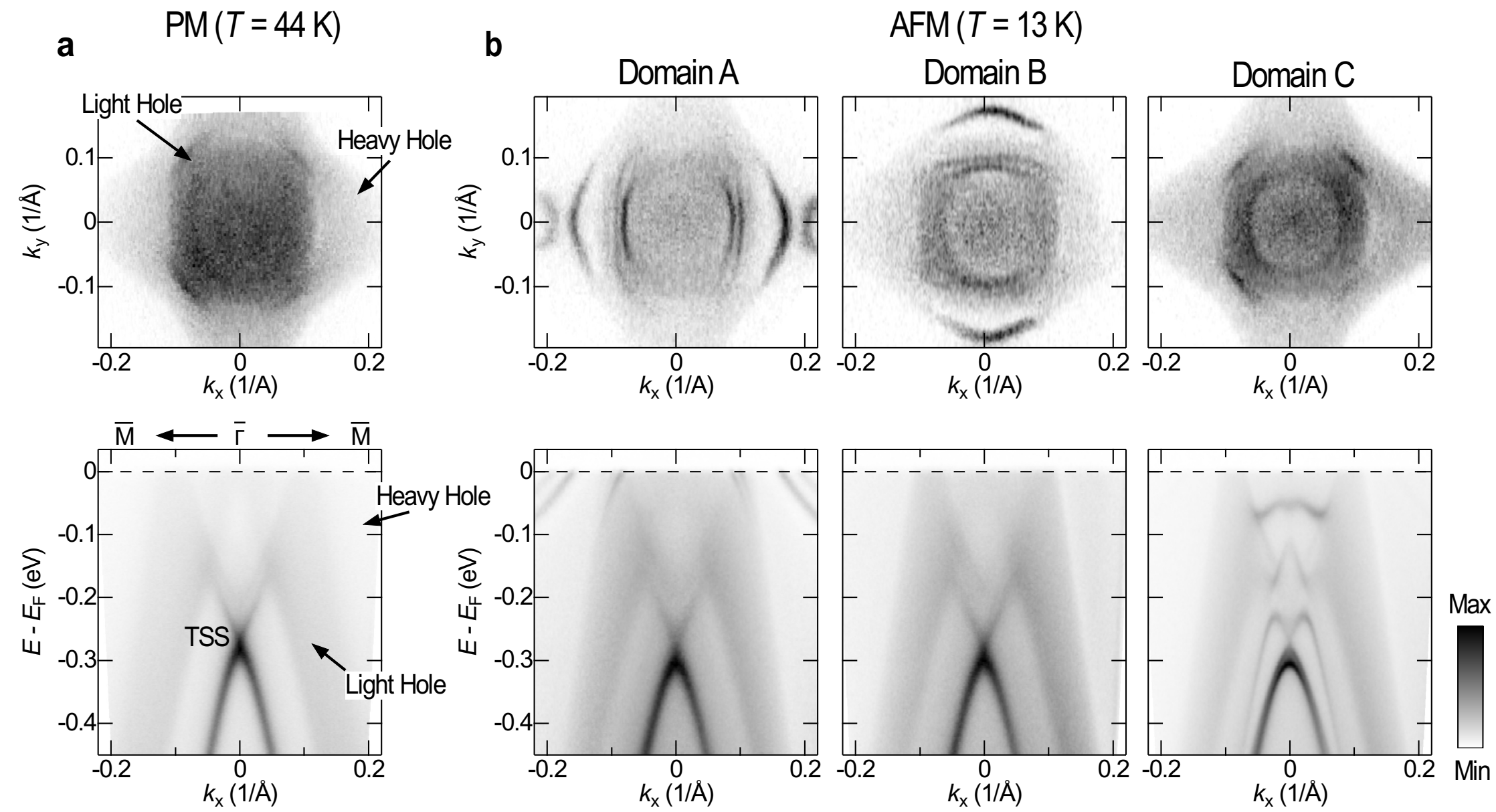

FIG. S3. Fermi surface mappings (top panels) and ARPES band dispersion images along the $\overline{\Gamma}$-$\overline{\mathrm{M}}$ direction (bottom panels) measured at $T = 44$ K (paramagnetic phase: PM, **a**) and at $T = 13$ K (antiferromagnetic phase: AFM, **b**) for the three magnetic domains (A-C). In the PM phase, distinct light-hole and heavy-hole bands are resolved, and the topological surface state (TSS) appears within the bulk band gap. Upon cooling into the AFM phase, the Fermi surface and band structure exhibit strong domain dependence, reflecting the different magnetic arrangements.

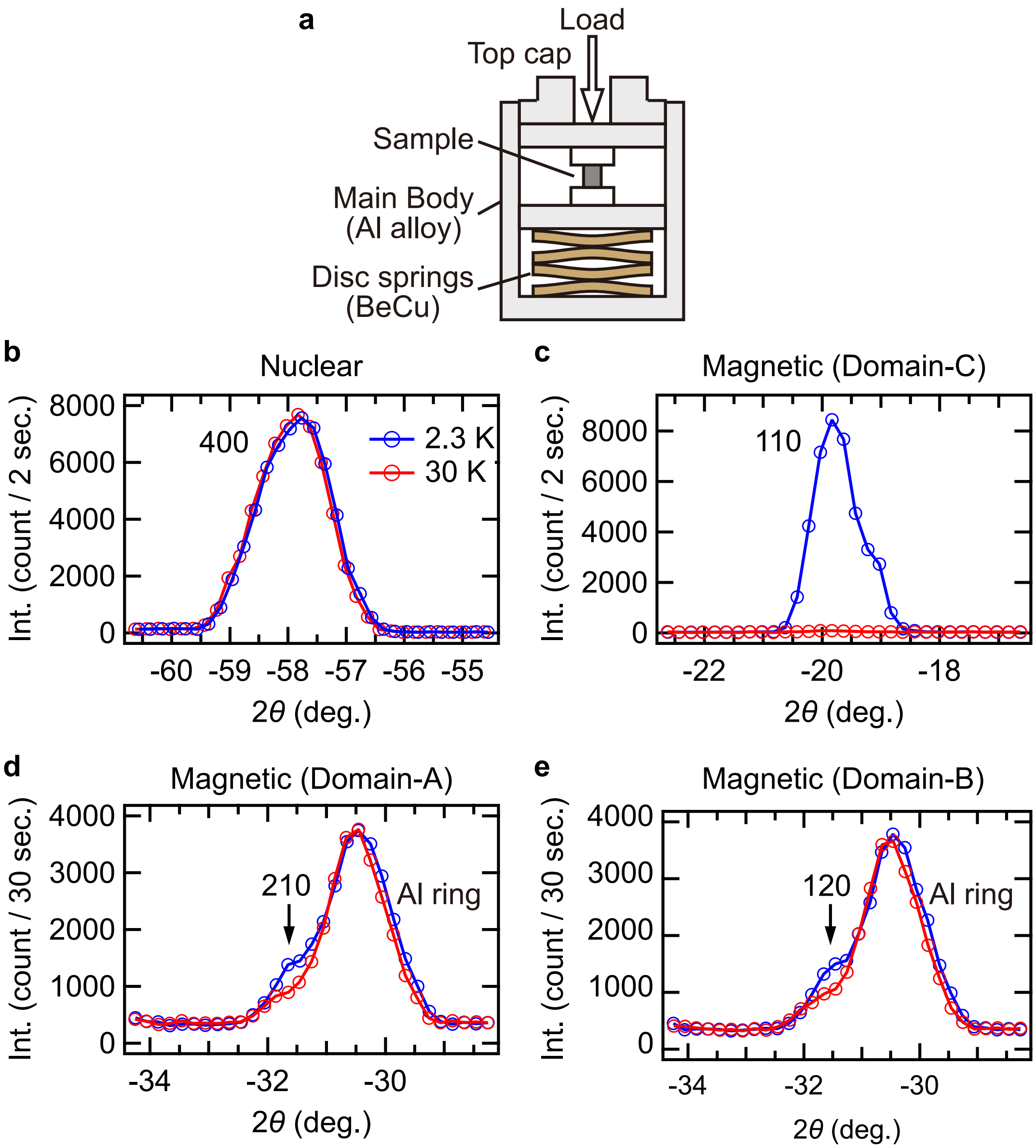

FIG. S4. **Temperature dependence of nuclear and magnetic Bragg reflections under uniaxial stress a**, Schematic of the uniaxial stress cell. The main body is constructed from an Al alloy, and the internal disc springs are made of BeCu. Uniaxial stress of 25 MPa is maintained by clamping the top cap. **b**, 400 nuclear Bragg reflection. **c**, 110 magnetic reflection from the AFM-C domain. **d,e**, 210 and 120 reflections corresponding to AFM-A and AFM-B, respectively. In panels **b**–**e**, data are shown for temperatures above T_N (30 K, red) and below T_N (2.3 K, blue). Black arrows denote magnetic peaks, while the temperature-independent background signals originate from the Al-alloy cell.

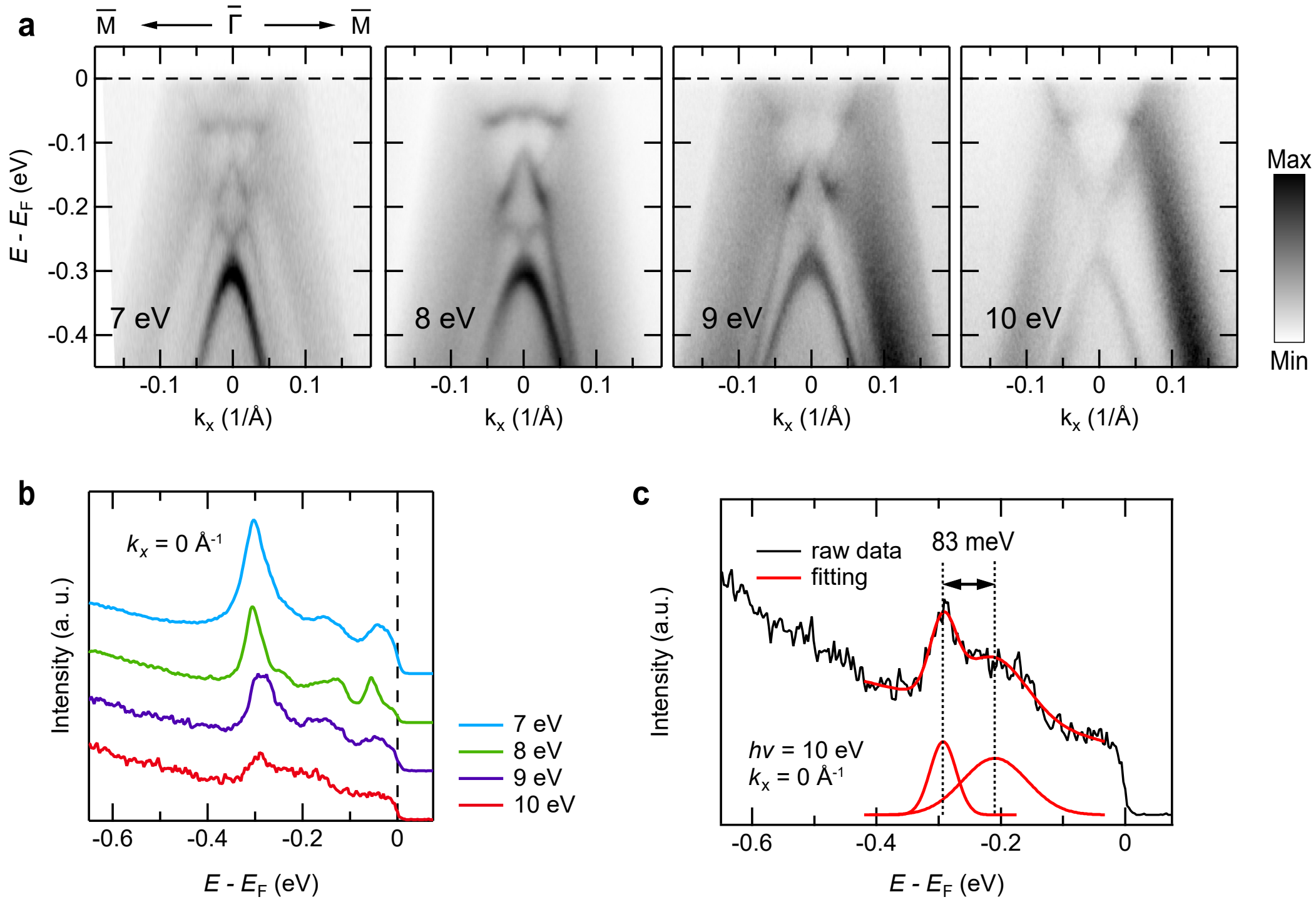

FIG. S5. **Photon-energy-dependent ARPES spectra measured along the $\overline{\Gamma}$-$\overline{\mathrm{M}}$ direction in the antiferromagnetic Domain C (AFM-C). a**, ARPES band dispersions recorded with photon energies of 7, 8, 9, and 10 eV. The TSS are clearly visible at 7 eV but become increasingly suppressed with higher photon energy. **b**, Corresponding energy distribution curves (EDCs) at $k_x = 0$ Å^{-1} extracted from the data in (a), highlighting the spectral evolution with photon energy. **c**, EDC at $k_x = 0$ Å^{-1} measured at $h\nu = 10$ eV (black), fitted with two Gaussian peaks (red), yielding an energy separation of 83 meV. This large gap originates from the bulk band and not the Dirac surface state, as discussed in the text.

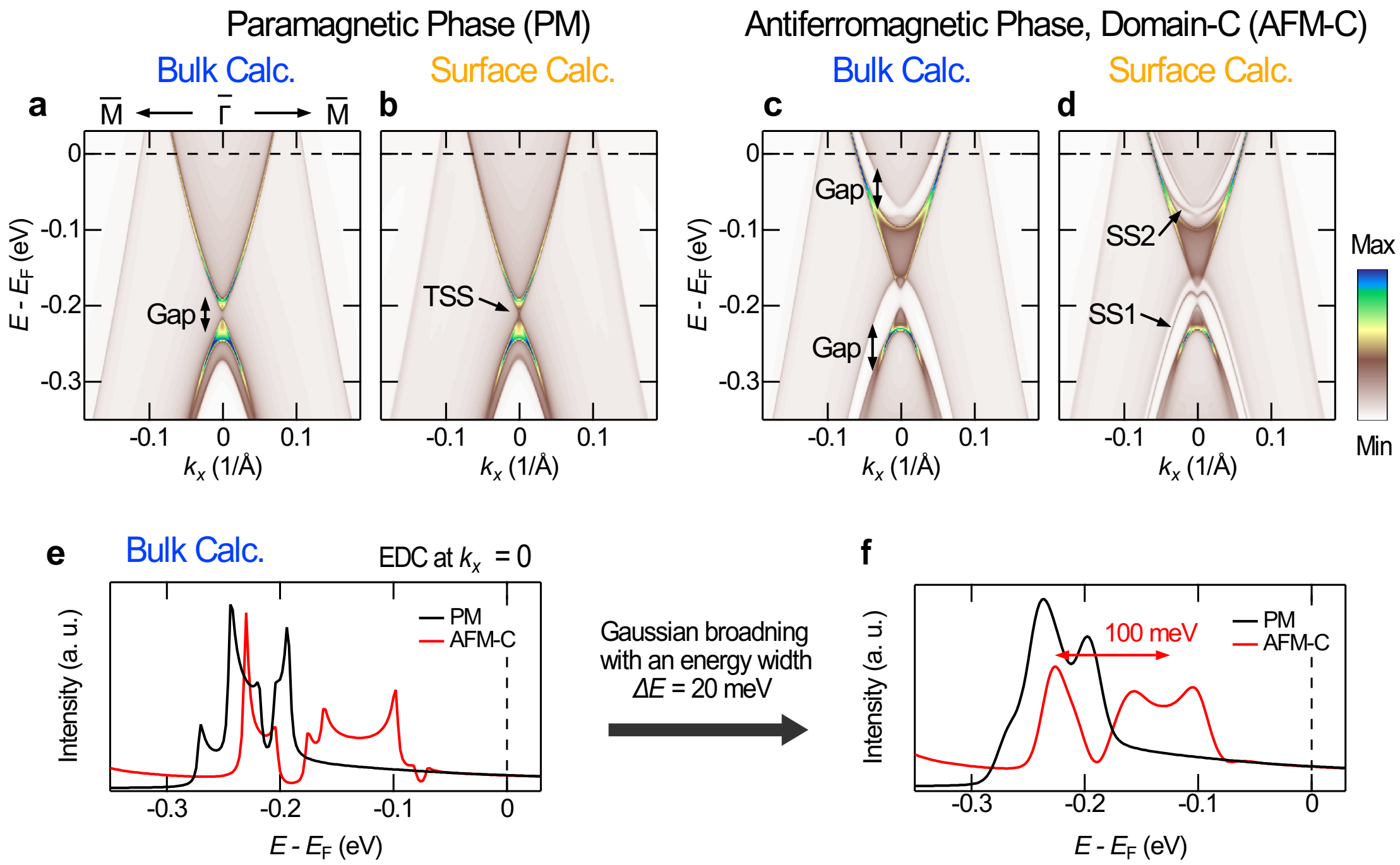

FIG. S6. **DFT band structure calculations along the $\bar{\Gamma}$-$\bar{M}$ direction for NdBi in the paramagnetic phase (PM) and antiferromagnetic phase, Domain-C (AFM-C). a**, The bulk band calculations that include only the bulk spectral weight in the PM phase. A small bulk gap is observed near the Dirac point. **b**, The surface band calculations that include both bulk and surface spectral weight in the PM phase, showing the emergence of a TSS within the bulk gap. **c**, The bulk band calculations, same as **a**, but for the AFM-C phase. Two distinct bulk gaps open due to antiferromagnetism. **d**, The surface band calculations, same as **b**, but for the AFM-C phase. Two surface states (SS1 and SS2) appear within the bulk gaps. **e**, Energy distribution curves (EDCs) at $k_x = 0$ Åextracted from the bulk spectra in **a** and **c**. Black and red lines correspond to the PM and AFM-C phases, respectively. **f**, Simulated EDCs obtained by convolving the spectra in **e** with a Gaussian of full width at half maximum (FWHM) of 20 meV. The AFM-C phase exhibits an enhanced gap of ∼100 meV, consistent with the bulk gap observed in high-photon-energy ARPES in Fig. S5.

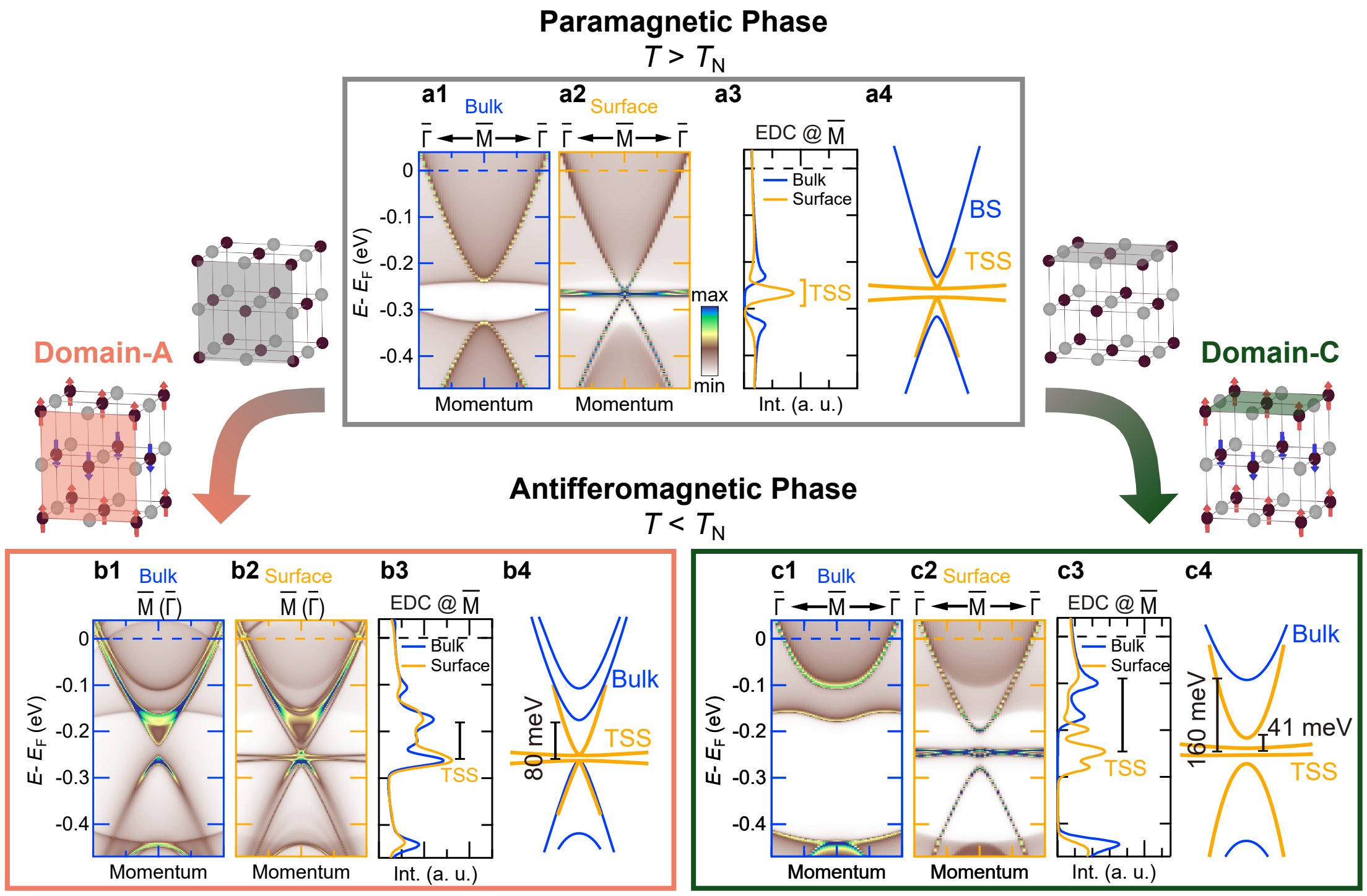

FIG. S7. **Calculated electronic structures around the $\bar{\mathrm{M}}$ point on the (001) surface. a1-a4**, Calculations for the paramagnetic phase. **b1-b4**, Calculations for the antiferromagnetic phase, Domain-A (AFM-A). **c1-c4**, Calculations for the antiferromagnetic phase, Domain-C (AFM-C). **a1,b1,c1**, Calculated bulk spectral weights. **a2,b2,c2**, Calculated surface spectral weights. **a3,b3,c3**, Energy distribution curves (EDCs) at the $\bar{\mathrm{M}}$ point extracted from the surface (orange) and bulk (blue) calculations. Energy resolution of 20 meV is applied to simulate a realistic spectrum. **a4,b4,c4**, Schematics of the band structures. Blue lines represent bulk states and orange lines represent topological surface states (TSS). In c4, the large energy separation involving bulk states (∼160 meV) and the intrinsic magnetic gap of the TSS (41 meV) are indicated.

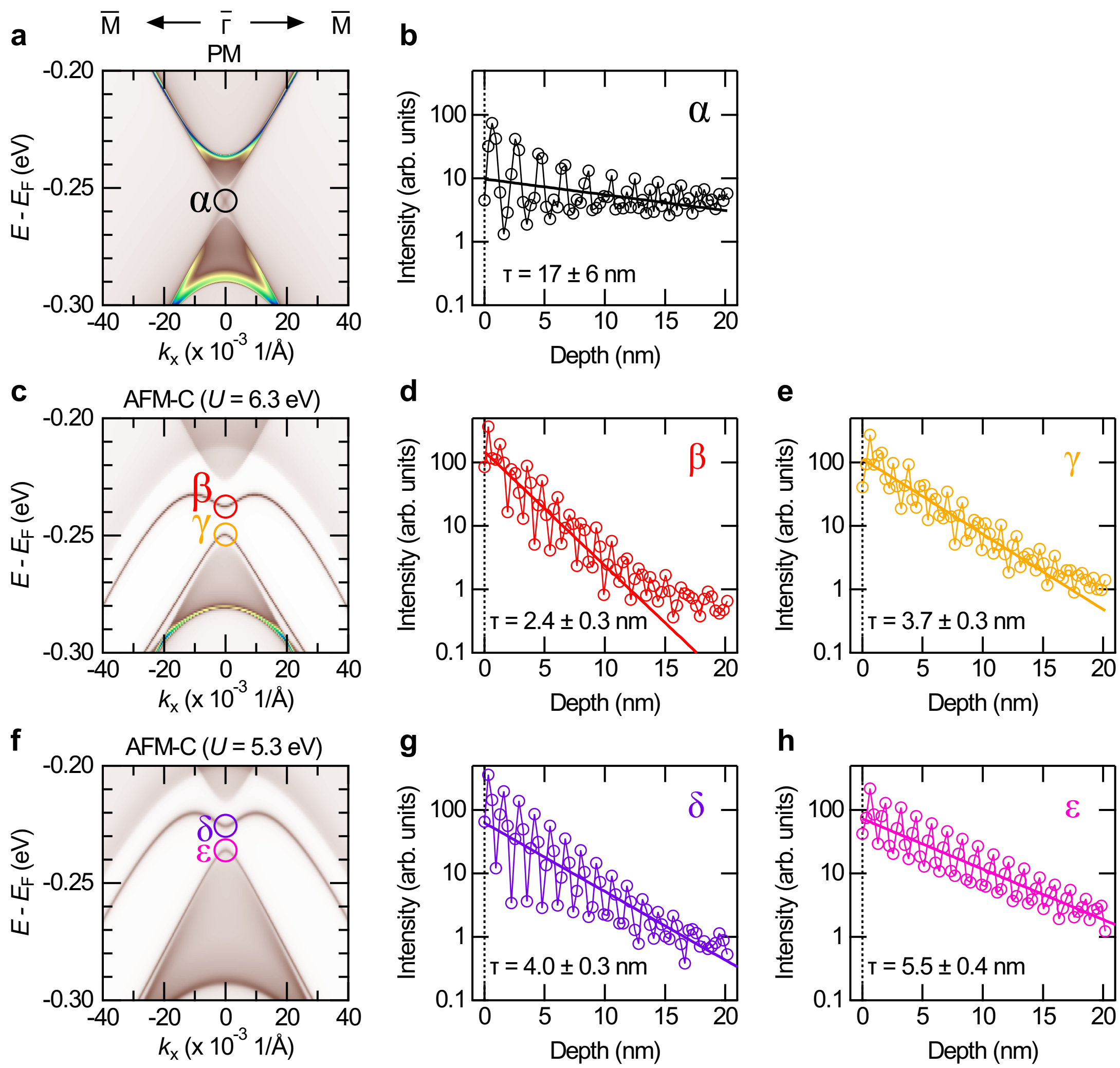

FIG. S8. **Penetration depth analysis of the TSS across the magnetic transition in NdBi, based on DFT+U calculations ($U = 6.3$ eV).** **a**, Surface band calculations of the paramagnetic (PM) phase showing the TSS at the marked energy-momentum point α. **b**, Layer-resolved spectral weight of the TSS calculated from the surface (Depth=0 nm) to the bulk for α. The decay length (τ) was determined by fitting the data with an exponential decay function (solid line), yielding $\tau = 17 \pm 6$ nm. **c**, Surface band calculations of the antiferromagnetic phase, Domain-C (AFM-C) using $U = 6.3$ eV, with two representative points (β, γ) selected for comparison. **d,e**, Layer-resolved spectral weight of the TSS calculated for β and γ. The decay length was determined as $\tau = 2.4 \pm 0.3$ nm for β and $\tau = 3.7 \pm 0.3$ nm for γ. **f**, Surface band calculations of the antiferromagnetic phase, Domain-C (AFM-C) using $U = 5.3$ eV, with two representative points (δ, ϵ) selected for comparison. **g,h**, Layer-resolved spectral weight of the TSS calculated for δ and ϵ. The decay length was determined as $\tau = 4.0 \pm 0.3$ nm for δ and $\tau = 5.5 \pm 0.4$ nm for ϵ.

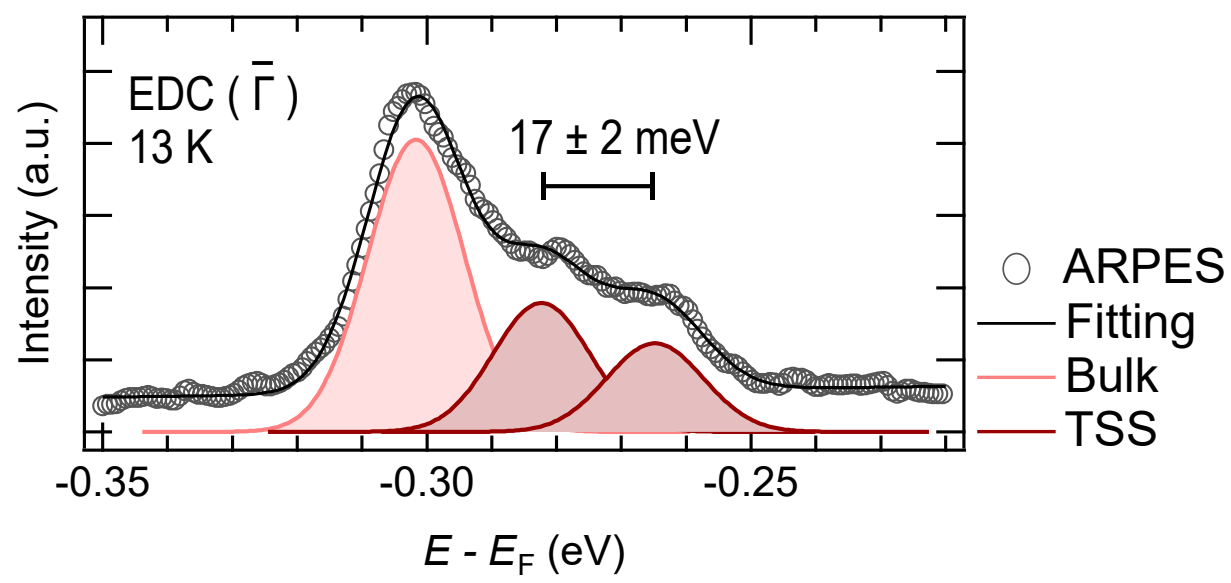

FIG. S9. **Quantitative determination of the topological Dirac gap size at the $\bar{\Gamma}$ point.** Energy distribution curve (EDC) at the $\bar{\Gamma}$ point measured at 13 K. The EDC was fitted using a combination of three Gaussian functions with a linear background. To stabilize the fitting, the widths of the three peaks were constrained to be equal.

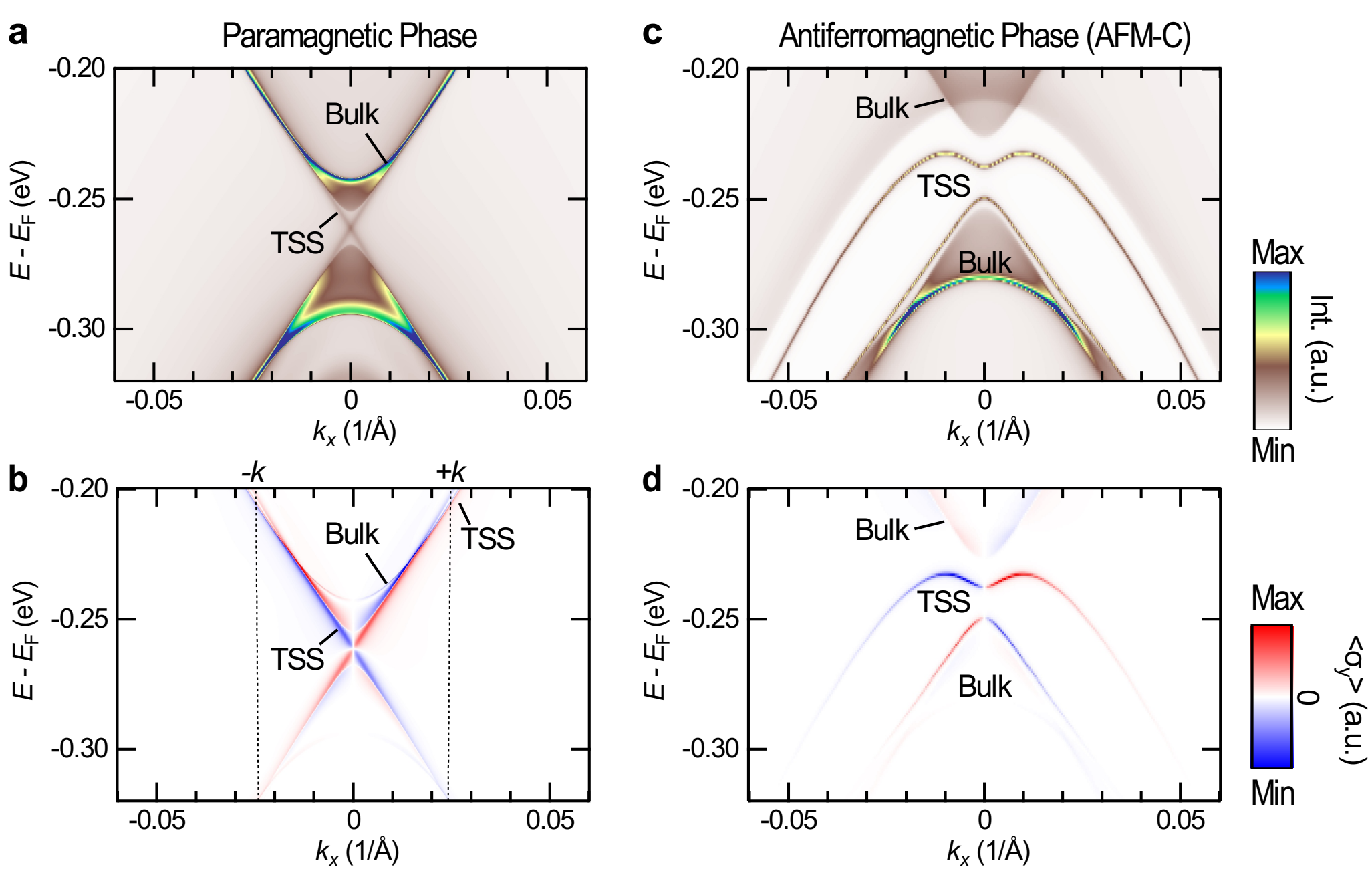

FIG. S10. **Spin-resolved DFT calculations of the electronic structure in the paramagnetic and antiferromagnetic phases. a**, Spin-integrated spectral weight map for the paramagnetic (PM) phase. The topological surface states (TSS) and bulk bands overlap in energy-momentum space. **b**, Spin-resolved spectral weight map for the paramagnetic phase. The color scale represents the expectation value for the y component of spin. The TSS is clearly distinguishable by its spin polarization, which has the opposite sign compared to that of the bulk bands. The dashed lines indicate the momentum cuts corresponding to the spin-resolved ARPES measurements presented in Fig. 3g of the main text. **c,d**, Spin-integrated and spin-resolved spectral weight maps for the antiferromagnetic phase (Domain-C), respectively. The calculations in c and d are the same as those used in Fig. 3i of the main text.

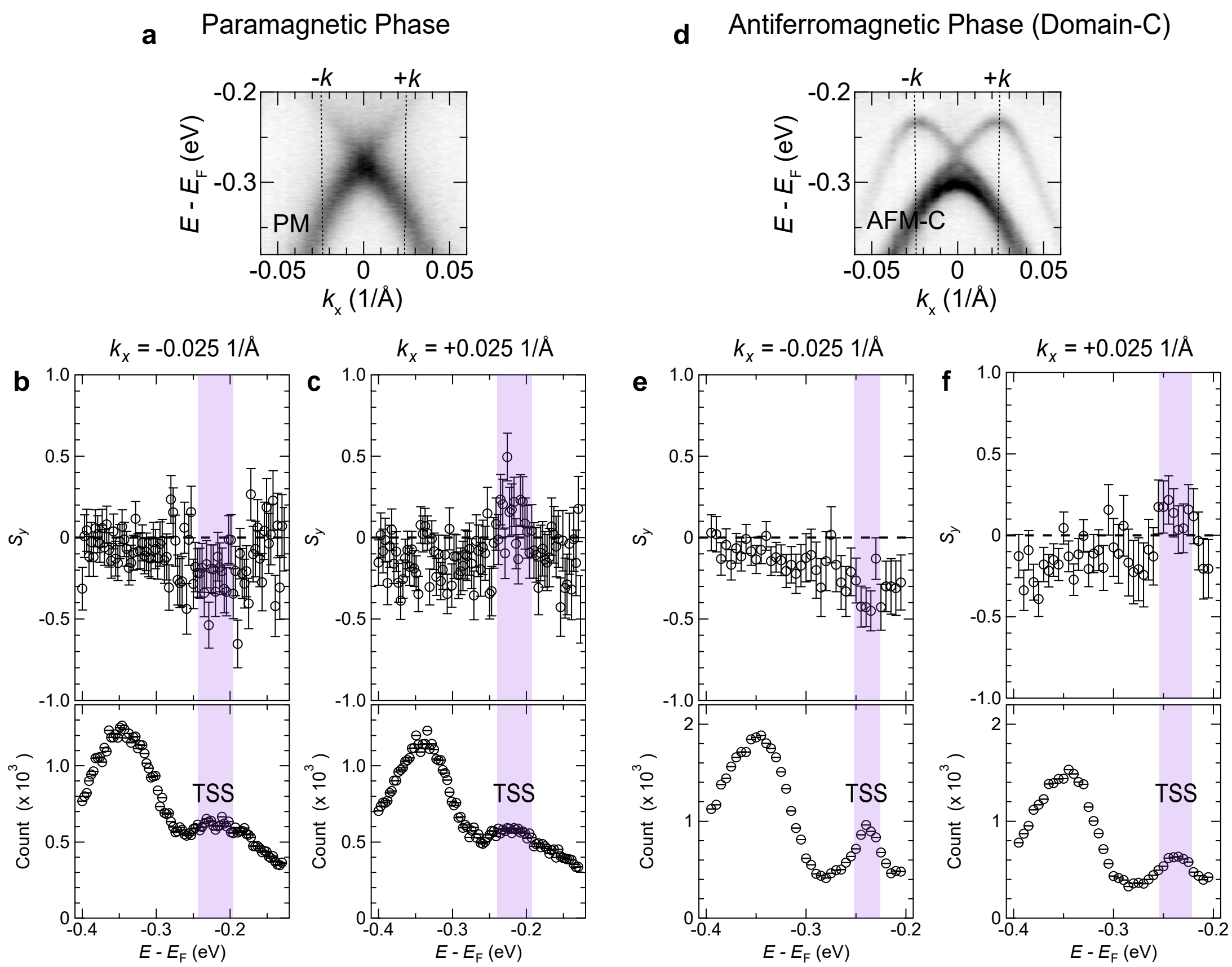

FIG. S11. **Spin polarization analysis of the TSS in the paramagnetic and antiferromagnetic phases.** **a**, ARPES intensity map along the $\bar{\Gamma}$–$\bar{\mathrm{M}}$ direction in the paramagnetic (PM) phase. The dashed vertical lines indicate the momentum positions ($\pm k$) where spin-resolved ARPES measurements were performed. **b,c**, Spin polarization (upper panel) and spin-integrated spectra (bottom panel) at $-k$ and $+k$ for the PM phase, respectively. **d**, ARPES intensity map in the antiferromagnetic phase, Domain-C (AFM-C). **e,f**, Spin polarization (upper panel) and spin-integrated spectra (bottom panel) at $-k$ and $+k$ for the AFM-C phase, respectively. The purple shaded regions highlight the spectral peaks originating from the topological surface states (TSS).

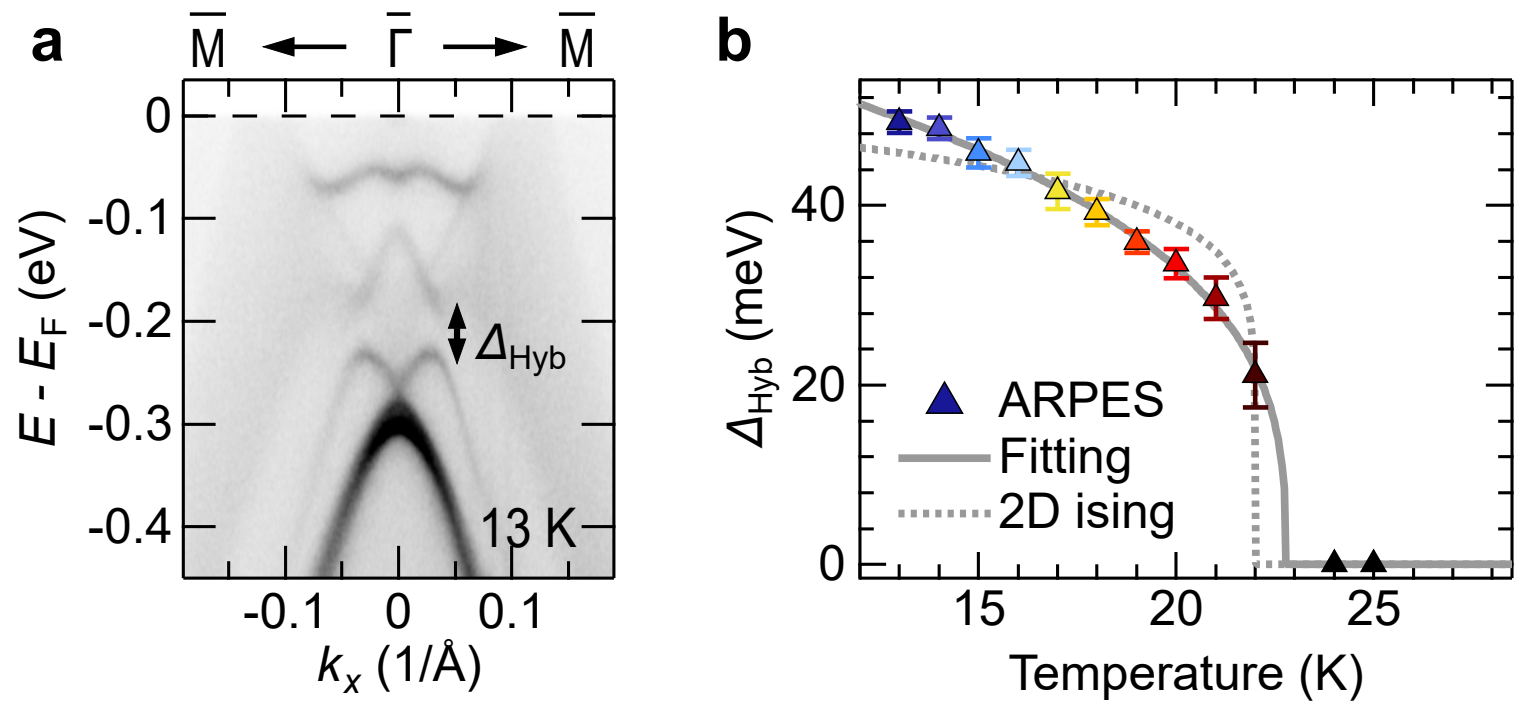

FIG. S12. **Critical scaling of the hybridization gap $\Delta_{\rm Hyb}(T)$.** **a**, ARPES intensity map of AFM-C domain at 13 K. **b**, Temperature dependence of $\Delta_{\rm Hyb}$. The solid gray curve is a power-law fit: $\Delta_{\rm Hyb} \propto (1 - T/T_{\rm N})^{\beta}$ with $\beta = 0.32(6)$ and $T_{\rm N} = 22.8(7)$ K. For comparison, a fit using the 2D Ising exponent ($\beta = 1/8$) is shown as a gray dotted line. The extracted exponent is consistent with the 3D Ising universality class.

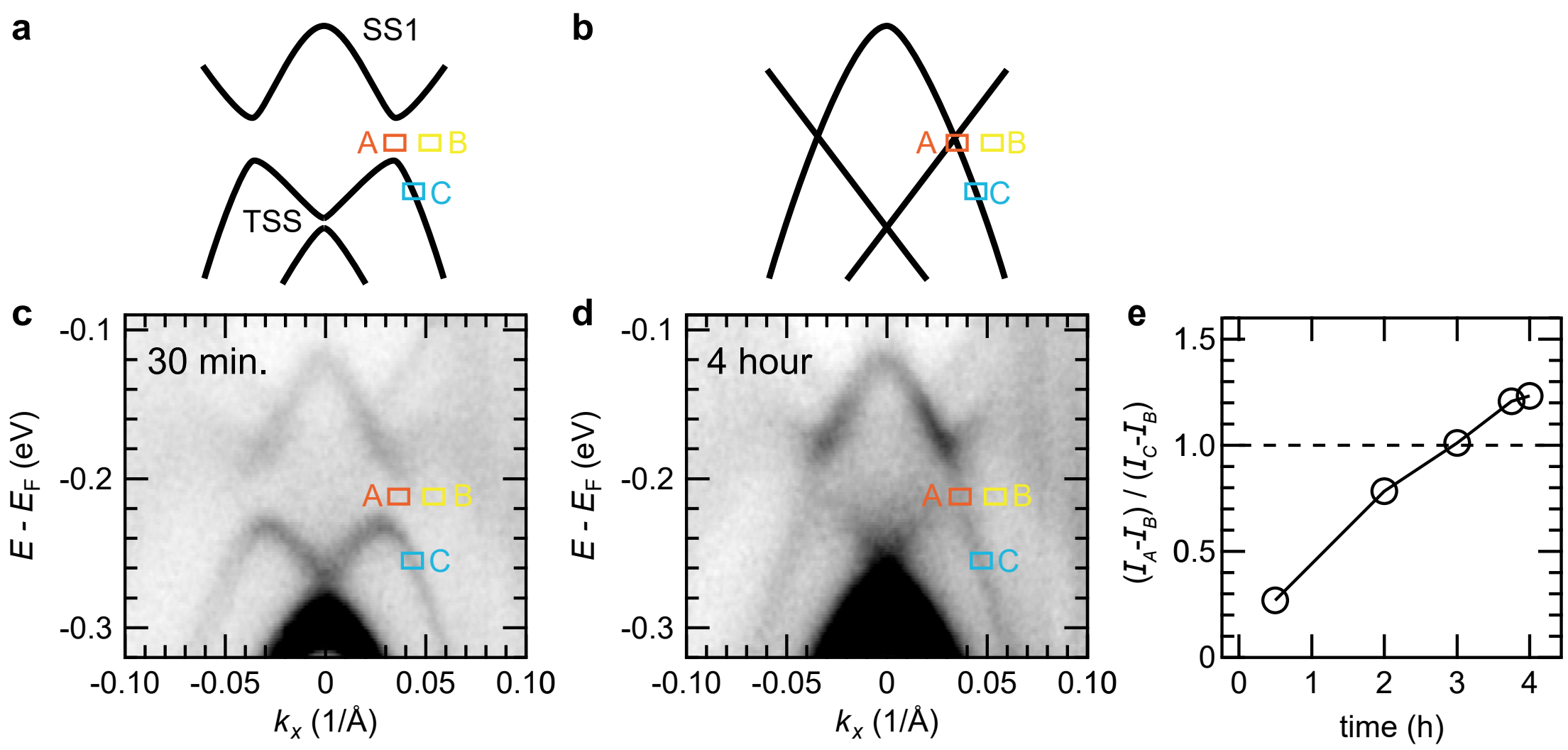

FIG. S13. **Effect of surface contamination on the electronic structure.** **a,b**, Schematics of the surface states for a clean surface (**a**) and a contaminated surface (**b**). On the clean surface, a gap $\Delta_{\rm Hyb}$ is formed between the topological surface state (TSS) and the surface state (SS1), whereas the gap collapses upon surface contamination. **c,d**, ARPES intensity maps measured at 30 minutes (**c**) and 4 hours (**d**) after cleavage. **e**, Time evolution of the photoelectron intensity within the gap. The intensity was calculated from the integrated spectral intensity of the A-C rectangular regions marked in **c** and **d**.

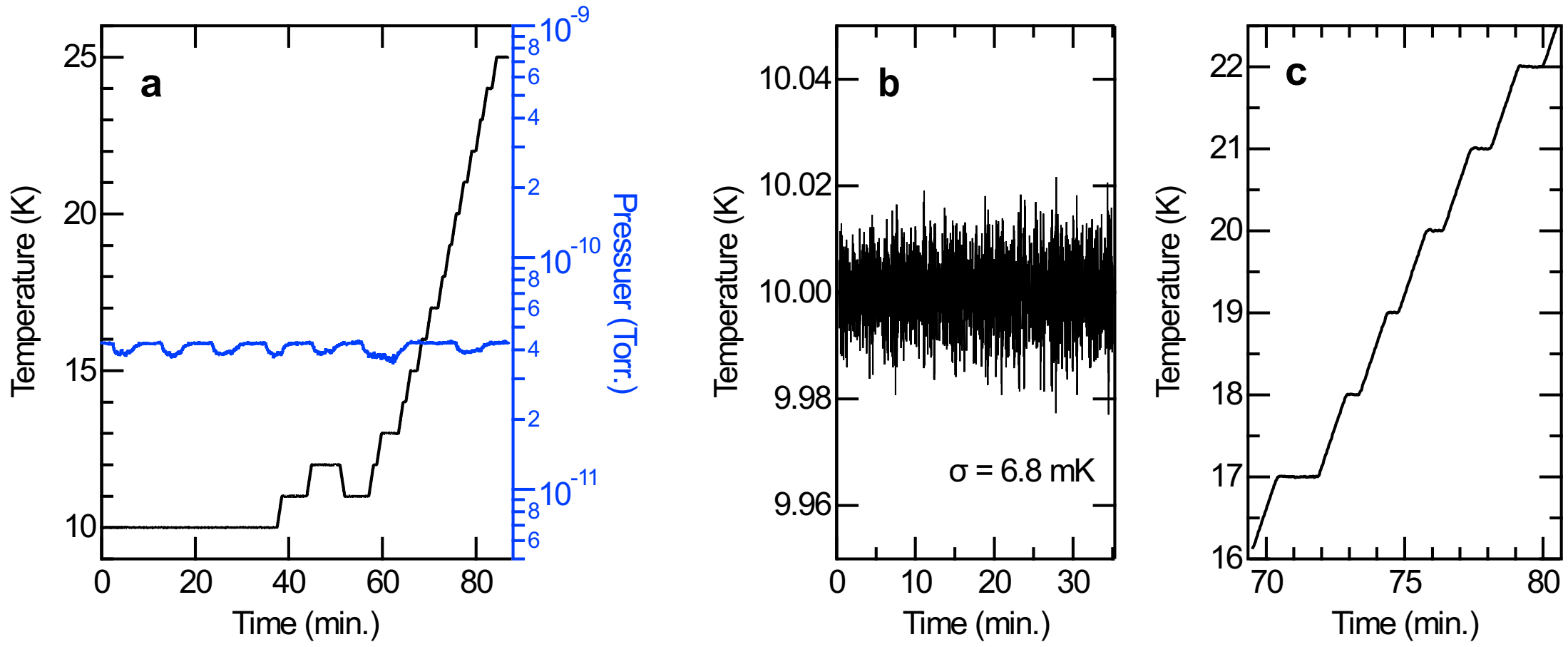

FIG. S14. **Stability of sample temperature and vacuum conditions.** **a**, Time evolution of the sample temperature (left) and vacuum pressure (right) under PID control. **b**, Temperature stability maintained at 10 K. The standard deviation over a 30-minute period is 6.8 mK. **c**, Temperature profile during a controlled ramp at a rate of 1 K/min.
