# Supplementary material for: Direct observation of a spin-polarized surface Dirac gap in the antiferromagnetic topological insulator NdBi
Source: Nat Commun. 2026 Sep 15;17:10841. doi: 10.1038/s41467-026-77571-4 (PMC13672286; doi:10.1038/s41467-026-77571-4)
Supplement: Supplementary file 2 [file 41467_2026_77571_MOESM2_ESM.pdf]

nature portfolio

Peer Review File

Direct observation of a spin-polarized surface Dirac gap in the antiferromagnetic topological insulator NdBi

Corresponding Author: Professor Takeshi Kondo

This file contains all reviewer reports in order by version, followed by all author rebuttals in order by version.

Version 0:

Reviewer comments:

Reviewer #1

(Remarks to the Author)
This manuscript reports observation of a spin-polarized surface Dirac gap in the AFM TI NdBi, using laser and spin-resolved ARPES. Inducing gap in the topological surface state (TSS) in magnetic TI remains an important question in the field. This paper studies the topological insulator NdBi, where the Nd 4f electrons introduce magnetism and strong electron correlations. High-resolution laser ARPES measurement, together with DFT calculations, provide evidence for a spin-polarized surface Dirac gap in NdBi. Spin-resolved ARPES measurements were also performed to understand the spin texture. Although I found that the laser ARPES data are of high quality and provide useful insight to understand the topological states in NdBi, I do not think that this paper is suitable for publication in Nature Communications. The following are my main concerns/questions:
1. The current experimental geometry is in fact not the ideal one for studying the gap in the TSS. In the previous study, Ref. [30], the energy-momentum dispersion of the TSS is determined by synchrotron-based ARPES measurements near the off-normal X bar point in the BZ. Under such measurement condition, the TSS and bulk band gaps are well separated (see Fig. 1(h) in Ref. [30]). In the current case, since the authors used laser ARPES, where the momentum range is limited, the identification of the gapped TSS has to rely on the measurement at the Gamma bar point at the zone center. Now due to the large kz broadening in ARPES experiment, bulk bands with all the kz's are projected onto the same in-plane momentum, and they can even overlap with the TSS. This makes it challenging to disentangle the bulk and surface states, as demonstrate in Fig. 2. Also, since ARPES still probes a range of kz (not uniformly covering the whole kz range), this adds further complexity to data interpretation (not considered in Fig. 2).
2. In addition to the possible overlap of bulk and surface bands, the strange photon energy dependence makes it hard to clearly distinguish bulk and surface bands. For example, as the authors mentioned in the manuscript (Page 5, last paragraph): "some of the bulk bands exhibit negligible kz dispersion, and significantly, the 2D-like bulk band displays an energy dispersion resembling a Dirac cone". This means that it is very difficult to experimentally identify bulk and 2D states. Also, the authors mention that the TSS can only be observed near 7 eV and it becomes absent above 7 eV. Such a strong photon energy dependence is very strange for a surface state, which is presumably 2D.
3. The spin-resolved ARPES data do not provide the expected spin splitting. In particular, the spin resolved cut in Fig. 3(g), i.e., along the dashed cuts in Fig. 3(a), is clearly different from the expected position of TSS derived from the bulk band inversion (see Fig. 2(f)). Specifically, as demonstrated in Fig. 2, the bulk gap near Gamma point is only ~16 meV, and the TSS should lie within this energy range. Therefore, the peaks highlighted by arrows in Fig. 3(g) correspond to bulk states, instead of TSS.
4. Based on the points above, it is unclear if the current paper presents a obviously stronger case compared to Ref. [30]. To me, Ref. [30] and the current paper address the same question, and, in Ref. [30], the TSS can be better separated and the measurement geometry is different. So I am really not convinced that the results in Ref. [30] are wrong.
5. The discussion related to Fig. 4(f) is very handwaving and lacks detailed support. How do the authors know experimentally the surface would lose magnetism without any surface degradation? The authors claim that the hybridization between SS1 and TSS is directly related to the surface magnetism. Why is so? The strength of hybridization is directly linked to the wave function overlap and it is unclear to me why it is relate do surface magnetism. Also in Fig. 4(e), the SS1 band clearly becomes diminished with increasing time, and it seems to suggest that sample degradation can be important.

Other minor issues:
1. Are calculations in Fig. 2 done in the PM phase? If so, it should be clearly explained.
2. In Fig. 2, the gapped Dirac-cone like band centered at Gamma bar shows up (and carry out significant weight) in both the

surface and bulk calculations. But this figure does not directly identify bulk and surface states. I would suggest that the authors do so for the sake of clarity.

Reviewer #2

(Remarks to the Author)
The authors present high-resolution laser ARPES and spin-ARPES evidence for a spin-polarized, gapped surface Dirac cone in the antiferromagnetic topological insulator (AFM-TI) NdBi. They identify a Dirac gap of ~15 meV, in close agreement with DFT (~12 meV), and argue that earlier reports of a much larger gap (~125 meV) likely misattributed bulk Dirac-like states to the topological surface state (TSS) due to photon-energy and resolution limitations. Temperature-dependent and time-evolution ("natural contamination") measurements reveal that the Dirac gap is magnetism-induced and depends on surface magnetic order, with a hybridization gap (Δhyb) following a 3D Ising-like order parameter.

2. Strengths
• Sub-3 meV resolution and spin selectivity clearly disentangle TSS from bulk states.
• Domain-resolved ARPES (AFM-A/B/C) properly accounts for magnetic symmetry and zone folding.
• Strong coherence between ARPES, spin-ARPES, DFT(+U), and Wannier-based surface calculations.
• Temperature and time evolution elegantly link surface magnetism to the Dirac gap.
• Credible reconciliation of AFM-TI inconsistencies (notably vs. $MnBi_{2n}Te_{3n+1}$) through photon-energy analysis.

3. Major Comments
• Dirac gap quantification
Provide uncertainties, number of samples/cleaves, repeatability, and fitting details (line shapes, background, confidence intervals). Include error bars for gap values in main figures.
• Spin-ARPES systematics
Report spin polarization vs. k and E with uncertainties; state Sherman function, asymmetry corrections, and resolution limits. A bulk-only reference cut showing negligible polarization would strengthen the argument.
• Domain identification
Add a concise domain-mapping overview (beam scans, symmetry fingerprints, occurrence rates) and, if possible, real-space maps illustrating domain selection.
• Photon-energy dependence
Move key hv-dependence data from the SI to the main text. Show 7/8/10 eV spectra side-by-side to highlight TSS suppression at higher photon energies.
• k_x–k_z dispersion
Demonstrate TSS k_z-independence (via photon-energy scans or limits). Even a compact confirmation would preempt alternative "quasi-2D bulk" interpretations.
• 3D Ising fit and TNT_NTN
Include fit uncertainties and goodness-of-fit metrics; overlay a 2D Ising comparison. Clarify how surface TNT_NTN was determined (it may deviate from bulk). If feasible, supplement with an independent magnetic probe.
• Surface magnetism corroboration
Support the contamination-driven closure of Δhyb and the Dirac gap with a surface-sensitive probe (XMCD, MOKE, SP-STM/STS, or ex-situ Kerr) on similarly prepared surfaces.
• Theory robustness
Discuss sensitivity to U,JU, JU,J, and slab thickness; quantify the TSS decay-length reduction (PM ≈ 17 nm → AFM-C ≈ 2–4 nm) and comment on surface termination effects.
• Claim language
Soften to "decisive" or "unambiguous under spin- and energy-resolved conditions," specifying the measurement parameters (7 eV, ΔE = 2.5 meV, domain control).
• Device relevance
Briefly relate gap and temperature scales to QAHE feasibility and compare to $MnBi_{2n}Te_{3n+1}$. Note prospects for thin films or electrostatic gating to enhance Δ and TNT_NTN.

4. Minor and Clarity Points
• Maintain consistent visual markers for TSS, SS1/SS2, and BVB; add inset momentum maps for orientation.
• Specify EDC fitting models (Voigt/Lorentzian), background type (Shirley/linear), and applied constraints.
• In time-evolution data, show pressure history, elapsed times, and gap vs. time with uncertainties; confirm thermal stability.
• Methods: report cleave temperature, surface termination statistics, beam-spot size vs. domain size, and sample-to-sample reproducibility.
• Deposit raw ARPES/momentum maps and DFT/Wannier inputs for reproducibility.

5. Important Controls
• Capping/dosing tests: Controlled O_2/H_2O exposure to quench and recover surface magnetism after mild annealing.
• Thickness dependence: Examine flakes or varying cleave thicknesses to assess TSS localization and gap evolution.
• Magnetic-field dependence: Apply modest out-of-plane fields to test Dirac gap alignment sensitivity.

6. Presentation
• Shorten the Abstract to emphasize:

(1) 15 meV spin-polarized surface gap;
(2) agreement with DFT;
(3) disentanglement of bulk/surface via 7 eV laser ARPES (ΔE = 2.5 meV) and spin-ARPES;
(4) linkage between surface magnetism and Dirac gap through temperature and contamination control.

• Standardize notation (TSS, BVB, Δhyb, TNT_NTN) and units (meV, eV, K).

Reviewer #3

(Remarks to the Author)
The manuscript "Direct observation of a spin-polarized surface Dirac gap in the antiferromagnetic topological insulator NdBi" by Yuto Fukushima et al. reports a laser-ARPES study of the antiferromagnetic topological insulator NdBi. The authors identify a Dirac-like surface state with an energy gap of approximately 15 meV, and attribute the gap opening to cooperative exchange interactions involving both bulk and surface magnetism, mediated by coupling between localized and itinerant carriers.
The manuscript is well written, and the figures display high-quality ARPES data with excellent resolution. The topic is timely and relevant to the community working on magnetic topological materials and correlated systems.
However, the novelty and context of this work require clearer articulation. In particular, this study shows significant overlap with the recent paper of A. Honma et al., Nature Communications 14, 7396 (2023), which reports that NdBi hosts two distinct surface electronic states depending on the orientation of the AFM domain, but with a much larger surface Dirac gap (>100 meV) under specific magnetic configurations.
This similarity with the metioend earlier work is not problematic in itself; indeed, complementary experiments using different photon energies that probe different depths and higher energy resolution can be valuable for clarifying the electronic and magnetic landscape. However, for readers to obtain a coherent and complete physical picture, the authors must:
1. Explicitly and more directly discuss the previous Nature Communications work in the Introduction.
2. Clarify how the present data relate to the scenario reported earlier.
3. Provide a more rigorous comparison of the magnitude, origin, and conditions of the Dirac gap.
Revising the manuscript in this direction is essential to deliver to the community a more complete and integrated understanding of the NdBi system and to avoid the impression of parallel or partially redundant conclusions. In doing so, the authors will strengthen the scientific value and impact of the present work.
Finally, I strongly recommend avoiding claims such as "for the first time" or similar priority statements.

Version 1:

Reviewer comments:

Reviewer #1

(Remarks to the Author)
I thank the authors for the efforts to address my concerns and questions. I think that the paper is much improved in terms of significance, clarity and distinctions from the previous papers. The paper should be acceptable for publication in Nature Communications in the current form.

Reviewer #3

(Remarks to the Author)
I am very satisfied with the effort that has been made to address all three referees' comments and suggestions. Most of the work has now been completed, and the revised manuscript, together with the accompanying supplementary information, is very impressive.

As a general comment, I believe that this work is an excellent example of what can be published in Nature Communications, as it allows for a more comprehensive presentation of the results and provides sufficient space to properly discuss and reflect on the novel findings. I would also like to emphasize that the referees have done an excellent job in providing constructive feedback, and that the authors have taken these comments very seriously, which has significantly improved the quality and impact of the manuscript. As a result, the work now offers greater value, particularly by building on previously published studies while still addressing important open questions.

Overall, I believe that this revised version is ready to be shared with the community and to further stimulate discussion on this topic in light of these results.

Open Access This Peer Review File is licensed under a Creative Commons Attribution 4.0 International License, which permits use, sharing, adaptation, distribution and reproduction in any medium or format, as long as you give appropriate credit to the original author(s) and the source, provide a link to the Creative Commons license, and indicate if changes were made.
In cases where reviewers are anonymous, credit should be given to 'Anonymous Referee' and the source.
The images or other third party material in this Peer Review File are included in the article's Creative Commons license, unless indicated otherwise in a credit line to the material. If material is not included in the article's Creative Commons license and your intended use is not permitted by statutory regulation or exceeds the permitted use, you will need to obtain permission directly from the copyright holder.
To view a copy of this license, visit https://creativecommons.org/licenses/by/4.0/

Response to Reviewer #1:

Comment 1.1

This manuscript reports observation of a spin-polarized surface Dirac gap in the AFM TI NdBi, using laser and spin-resolved ARPES. Inducing gap in the topological surface state (TSS) in magnetic TI remains an important question in the field. This paper studies the topological insulator NdBi, where the Nd 4f electrons introduce magnetism and strong electron correlations. High-resolution laser ARPES measurement, together with DFT calculations, provide evidence for a spin-polarized surface Dirac gap in NdBi. Spin-resolved ARPES measurements were also performed to understand the spin texture. Although I found that the laser ARPES data are of high quality and provide useful insight to understand the topological states in NdBi, I do not think that this paper is suitable for publication in Nature Communications.

Answer:

We sincerely thank the reviewer for the careful assessment of our manuscript and for recognizing the high quality of our laser-ARPES data and the insight they provide into the topological states in NdBi. We also appreciate the reviewer's acknowledgement that opening and identifying a gap in the topological surface state of a magnetic topological insulator remains an important question in the field.

While we respect the referee's overall assessment at this stage, we believe that the present work addresses a central and long-standing challenge in antiferromagnetic topological insulators—namely, the unambiguous identification of an intrinsic surface Dirac gap in the presence of overlapping bulk states—and thereby meets the criteria for publication in Nature Communications. In particular, previous ARPES studies have been unable to disentangle intrinsic surface gaps from bulk projections due to k_z broadening and limited experimental selectivity. In contrast, our study combines (i) high-resolution 7 eV laser ARPES with an energy resolution of 2.5 meV, (ii) spin-resolved ARPES, (iii) magnetic-domain-selective measurements, and (iv) surface and bulk spectral calculations, to achieve a clear separation of the intrinsic topological surface state from bulk-derived Dirac-like features in NdBi.

We further demonstrate that the observed gap is intrinsic to the topological surface state, rather than an apparent feature arising from bulk-band projections or matrix-element effects. This conclusion is consistently supported by its spin polarization, its quantitative agreement with the calculated intrinsic surface-state gap, and its systematic evolution with temperature and surface aging. In this sense, the present work does not simply add another observation of gapped bands in NdBi; rather, it provides a rigorous and unambiguous identification of the intrinsic magnetic gap of the topological surface state under experimentally controlled conditions designed to disentangle it from nearby bulk contributions.

In the following responses, we address each of the reviewer's concerns in detail and provide additional calculations and analyses that directly address these points and further reinforce the robustness of our conclusions.

Comment 1.2

The following are my main concerns/questions:

1. The current experimental geometry is in fact not the ideal one for studying the gap in the TSS. In the previous study, Ref. [30], the energy-momentum dispersion of the TSS is determined by synchrotron-based ARPES measurements near the off-normal X bar point in the BZ. Under such measurement condition, the TSS and bulk band gaps are well separated (see Fig. 1(h) in Ref. [30]). In the current case, since the authors used laser ARPES, where the momentum range is limited, the identification of the gapped TSS has to rely on the measurement at the Gamma bar point at the zone center. Now due to the large kz broadening in ARPES experiment, bulk bands with all the kz's are projected onto the same in-plane momentum, and they can even overlap with the TSS. This makes it challenging to disentangle the bulk and surface states, as demonstrate in Fig. 2. Also, since ARPES still probes a range of kz (not uniformly covering the whole kz range), this adds further complexity to data interpretation (not considered in Fig. 2).

Answer:

We thank the reviewer for this important comment. We agree that measurements near the X-bar point benefit from a clearer separation between surface and bulk bands. Before discussing this momentum region in detail, we first note that such measurements probe states that do not directly reflect the underlying nontrivial Z_2 topology.

In NdBi, single Dirac cone is located at the Γ-bar point, where it represents the odd-number of topological surface state (TSS) required by the nontrivial Z_2 invariant. While TSSs are also observed at the M-bar point, these arise from the projection of multiple bulk band inversions and give rise to an even number of Dirac cones. Such states appeared at the M-bar point may not be topologically protected therefore Dirac cones at the M-bar is not suitable to investigate the interaction between magnetism and the nontrivial Z_2 topology. This possibility is also mentioned in Ref [30] (Page 3, last paragraph).

We agree that resolving the Dirac point at the Γ-bar point is experimentally challenging due to k_z broadening, which leads to overlap with projected bulk states. Nevertheless, direct observation of a Dirac gap for the topological Dirac cone at the Γ-bar point remains indispensable for identifying the antiferromagnetic topological phase in this system.

Because of this intrinsic difficulty, the identification cannot rely solely on energy-momentum intensity maps and requires going beyond conventional ARPES approaches. This difficulty arises because bulk-derived Dirac-like bands and k_z broadening fundamentally obscure the surface contribution in conventional ARPES measurements.

In this work, we combine high-resolution 7 eV laser ARPES with spin-resolved measurements. Spin-resolved ARPES is essential to distinguish the spin-polarized topological surface state from bulk-derived bands. This allows a direct identification of the TSS. Importantly, the gap opening at the Dirac point of the spin-polarized surface band is in quantitative agreement with our DFT surface calculations, further supporting the intrinsic origin of the observed Dirac gap. Taken together, the consistency between spin-resolved ARPES and surface-state calculations provides a compelling and self-consistent identification of the topological Dirac gap.

Still, we agree with the reviewer that measurements at the M-bar point, where surface and bulk states are more clearly separated, can be experimentally advantageous for evaluating the impact of magnetism on the opening of a Dirac gap in NdBi associated with time-reversal symmetry breaking.

Although the 7 eV laser ARPES employed in this work does not access this momentum region due to its limited photon energy, our DFT calculations offer a consistent explanation for the previously reported large gap in Ref. [30], attributing it to bulk-band features rather than to the intrinsic topological surface state. This discrepancy can be understood based on two key points.

1) While Ref. [30] attributed sharp spectral features primarily to surface states, our combined bulk and surface calculations demonstrate that sharp dispersive features can arise from both surface and projected bulk bands.

2) In the present work, we experimentally distinguish surface and bulk contributions using spin-resolved laser ARPES, which enables a more reliable identification of the topological surface state compared to approaches based solely on spectral intensity.

We further note that Ref. [30] reported a similarly large gap at the Dirac point of the TSS for Domain-A (corresponding to Domain B in Ref. [30]), where the Dirac point is expected to remain gapless due to symmetry. This apparent discrepancy can be naturally understood within our interpretation, in which the observed large gap originates from bulk-band features rather than from the intrinsic surface state. We emphasize again that sharp spectral features do not necessarily indicate a surface origin. As demonstrated by our bulk calculations, projected bulk bands can also exhibit sharp dispersions, which can lead to an overestimation of the surface-state gap if not properly distinguished.

The above-mentioned mechanism is summarized in Fig. R1. We present DFT calculations around the M-bar point above and below T_N (top and bottom panels, respectively). The reliability of these calculations is supported by their excellent agreement with our ARPES results around the Γ-bar point. We analyze the electronic structures for Domain-C and Domain-A separately and discuss the origin of the discrepancy with Ref. [30]. In particular, by comparing the temperature evolution of bulk and surface band calculations in each domain, we provide a consistent explanation for the previously reported large gap.

Domain C:

Figures R1a1 and R1a2 show the spectral intensity maps of bulk and surface calculations in the paramagnetic phase above T_N, while the corresponding results in the antiferromagnetic phase below T_N are shown in Fig. R1(c1,c2). Upon entering the AF phase, the bulk gap becomes significantly enlarged.

In contrast, the intrinsic gap at the Dirac point of the TSS at the M-bar point remains much smaller, about 41 meV (Fig. R1(b5)). Notably, the energy separation between the nearly flat TSS branch and the bulk bands is approximately 160 meV (Fig. R1(c4)), which is comparable to the gap size reported as the TSS gap in Ref. [30].

This comparison indicates that the previously reported large gap can be attributed to the energetic separation between surface and bulk states, rather than to the intrinsic magnetic gap of the TSS. This result highlights the importance of reliably distinguishing surface and bulk contributions, as achieved here using spin-resolved ARPES.

Domain A:
Ref. [30] also reported a similarly large gap at the Dirac point of the TSS for Domain A, where the Dirac point is expected to remain symmetry-protected and gapless. In Ref. [30], the origin of this large gap was not identified.

Within our framework, however, this observation can be consistently explained by bulk-band contributions rather than by an intrinsic gap opening of the TSS Dirac point. Supporting DFT calculations are presented in Fig. R1(b).

We have added these arguments, together with Fig. R1, in the revised version of our manuscript.

The large gap reported in Ref. [30] is likely influenced by the photon energy used in the ARPES measurements (*hv* = 60 eV). In our experiments, we find that the spectral weight of the TSS decreases with increasing photon energy and becomes nearly undetectable above ~10 eV, where bulk-band contributions dominate.

A similar photon-energy dependence has been reported in another antiferromagnetic topological insulator, $MnBi_2Te_4$ (**Chen et al., Phys. Rev. X 9, 041040 (2019); Hao et al., Phys. Rev. X 9, 041038 (2019)**), where higher photon energies predominantly probe bulk states with a large gap in the antiferromagnetic phase, while lower photon energies are required to clearly resolve the surface bands.

To further emphasize this point, and in response to a request from another reviewer, we have moved the photon-energy-dependent ARPES data around the Γ-bar point to the main text (new Fig. 4). These results clearly demonstrate that the surface states are distinctly resolved only at low photon energies around 7 eV.

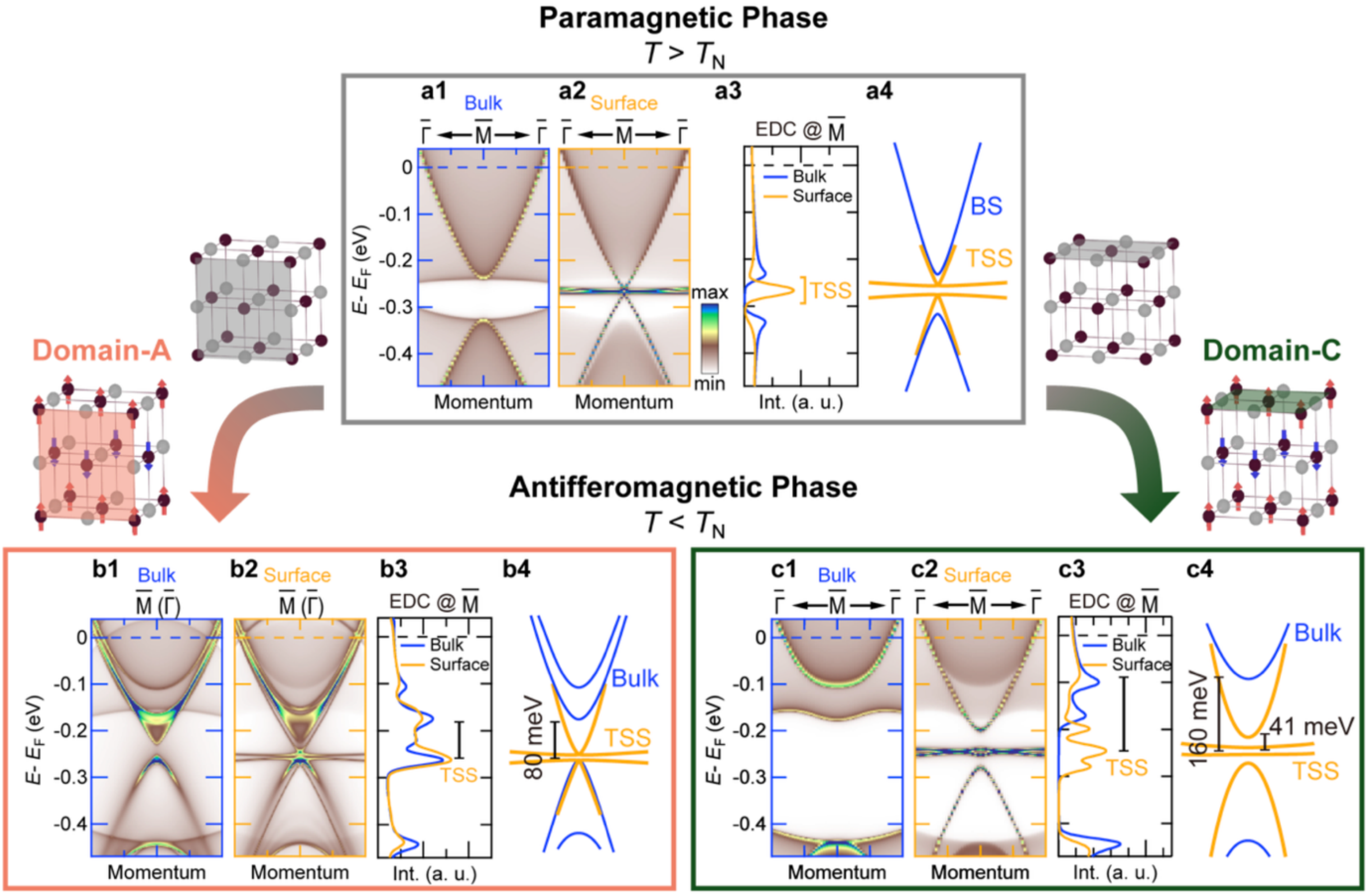

Figure R1: **Calculated electronic structures around the $\bar{M}$ point at (001) surface. a1–a5** Calculations for the paramagnetic phase. **b1–b5** Calculations for the antiferromagnetic phase, Domain-A (AFM-A). **c1–c5** Calculations for the antiferromagnetic phase, Domain-C (AFM-C). **a1, b1, c1** Calculated bulk spectral weights. **a2, b2, c2** Calculated surface spectral weights. **a3, b3, c3** Energy distribution curves (EDCs) at the $\bar{M}$ point extracted from the surface (orange) and bulk (blue) calculations. Energy resolution of 20 meV is applied to get a realistic spectrum. **a4, b4, c4** Schematics of the band structures. Blue lines represent bulk states and orange lines represent topological surface states (TSS). In c4, the large energy separation involving bulk states (~160 meV) and the intrinsic magnetic gap of the TSS (41 meV) are indicated.

Comment 1.3

2. In addition to the possible overlap of bulk and surface bands, the strange photon energy dependence makes it hard to clearly distinguish bulk and surface bands. For example, as the authors mentioned in the manuscript (Page 5, last paragraph): "some of the bulk bands exhibit negligible kz dispersion, and significantly, the 2D-like bulk band displays an energy dispersion resembling a Dirac cone". This means that it is very difficult to experimentally identify bulk and 2D states. Also, the authors mention that the TSS can only be observed near 7 eV and it becomes absent above 7 eV. Such a strong photon energy dependence is very strange for a surface state, which is presumably 2D.

Answer:

We thank the reviewer for the insightful comments regarding (1) the difficulty in distinguishing bulk and surface bands and (2) the unusual photon-energy dependence of the topological surface state (TSS). We address these points separately below.

(1) Distinguishing bulk states from the 2D surface state:

We agree that band dispersion alone (i.e., energy-momentum intensity maps) is insufficient to distinguish TSS from quasi-2D bulk states, as they can exhibit similar Dirac-like dispersions. In this work, we employ spin-resolved ARPES as a decisive probe. Our measurements clearly reveal spin-momentum locking, a hallmark of a TSS that is absent in spin-degenerate bulk bands, thereby enabling an unambiguous distinction between surface and bulk contributions.

Furthermore, our surface calculations quantitatively reproduce the observed band dispersion, providing additional and independent support for the surface origin of the TSS.

(2) Photon energy dependence of the TSS:

We acknowledge the reviewer's concern that the strong photon-energy dependence appears counterintuitive for a two-dimensional surface state. However, closely analogous behavior has been reported in $MnBi_2Te_4$, which is well established as an antiferromagnetic topological insulator [**Chen, Y. J. et al., Phys. Rev. X 9, 041040 (2019); Hao, Y.-J. et al., Phys. Rev. X 9, 041038 (2019)**]. In these studies, the TSS is clearly resolved at low photon energies but becomes strongly suppressed at higher photon energies (e.g., above ~20 eV), where bulk spectral weight dominates.

This behavior suggests that the apparent disappearance of the TSS at higher photon energies originates from matrix-element effects and the enhanced contribution of bulk states, rather than from the absence of the surface state itself. Our observations are therefore consistent with this established behavior and might indicate that such photon-energy dependence is not anomalous but intrinsic to photoemission from antiferromagnetic topological insulators.

In the revised manuscript, we have further emphasized the similarity between NdBi and $MnBi_2Te_4$—two of the few established candidates for antiferromagnetic topological insulators—and have moved the photon-energy-dependent ARPES data to the main text.

Comment 1.4

3. The spin-resolved ARPES data do not provide the expected spin splitting. In particular, the spin resolved cut in Fig. 3(g), i.e., along the dashed cuts in Fig. 3(a), is clearly different from the expected position of TSS derived from the bulk band inversion (see Fig. 2(f)). Specifically, as demonstrated in Fig. 2, the bulk gap near Gamma point is only ~16 meV, and the TSS should lie within this energy range. Therefore, the peaks highlighted by arrows in Fig. 3(g) correspond to bulk states, instead of TSS.

Answer:

We agree with the reviewer that the TSS and bulk bands are energetically close in the paramagnetic (PM) phase, making their distinction nontrivial. To address this issue and unambiguously identify the origin of the observed signals, we performed additional spin-resolved DFT calculations for the PM phase (Fig. R2).

Figure R2a shows the surface spectral function (spin-integrated) for the PM phase. We also calculated the corresponding bulk bands in the main manuscript (Fig. 2e), allowing us to distinguish between surface and bulk contributions in the spectral function, as indicated in Fig. R2a.

Consistent with the reviewer's comment, Figure R2a shows that the TSS and bulk bands overlap in energy-momentum space, making them indistinguishable in spin-integrated spectra. This ambiguity, however, is resolved by considering spin polarization. Figure R2b presents the corresponding spin-resolved spectral function. The TSS appears at the edge of the bulk continuum and exhibits a clear spin polarization. Although the bulk states exhibit weak spin polarization due to surface-induced inversion symmetry breaking, their spin polarization is significantly weaker and, importantly, of opposite sign to that of the TSS.

We further performed spin-resolved calculations for the antiferromagnetic (AFM) phase (Figs. R2c and R2d). The results show that the sign of the spin polarization of the TSS remains unchanged across the AFM transition. In the AFM phase, the TSS is well separated from the bulk bands and can be clearly identified (see Figs. 3g and 3h in the main manuscript), providing a reliable benchmark for interpreting the experimental data. We find that the same spin polarization is observed in the PM phase, indicating that the feature assigned as the TSS in the PM phase originates from the same surface state. If the peaks were instead derived from bulk states, their spin polarization would exhibit the opposite sign according to our calculations; however, this is not observed. This directly rules out a bulk origin of the observed features.

The consistency of the spin polarization between the PM and AFM phases provides strong evidence that our 7 eV laser ARPES selectively probes the TSS even in the PM phase. In our measurements, the bulk contribution is strongly suppressed, likely reflecting a combination of intrinsically weaker bulk spectral weight and photoemission matrix element effects, which enhance the relative visibility of the surface state.

We have added these arguments and Fig.R2 in the revised version of our manuscript.

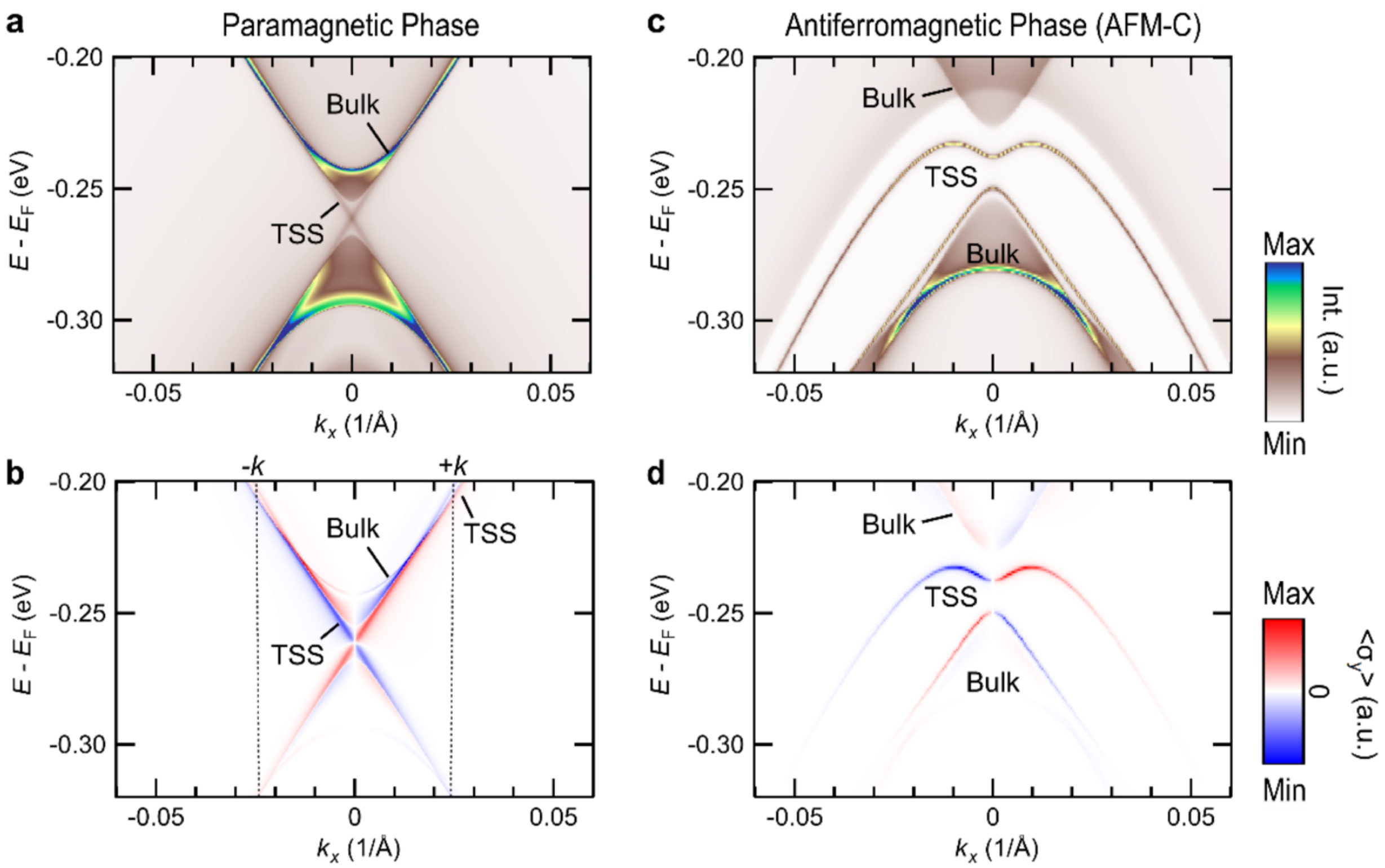

Figure R2: **Spin-resolved DFT calculations of the electronic structure in the paramagnetic and antiferromagnetic phases. a** Spin-integrated spectral weight map for the paramagnetic (PM) phase. The topological surface states (TSS) and bulk bands overlap in energy-momentum space. **b** Spin-resolved spectral weight map for the paramagnetic phase. The color scale represents the expectation value for the y component of spin. The TSS is clearly distinguishable by its spin polarization, which has the opposite sign compared to the spin polarization of the bulk bands. The dashed line indicates the momentum cut corresponding to the spin-resolved ARPES measurement presented in Figure 3g in the main manuscript. **c, d** Spin-integrated and spin-resolved spectral weight maps for the antiferromagnetic phase (Domain-C), respectively. Note that the data presented in c and d correspond to the calculations used for Figure 3i in the main manuscript.

Comment 1.5

4. Based on the points above, it is unclear if the current paper presents a obviously stronger case compared to Ref. [30]. To me, Ref. [30] and the current paper address the same question, and, in Ref. [30], the TSS can be better separated and the measurement geometry is different. So I am really not convinced that the results in Ref. [30] are wrong.

Answer:

We thank the reviewer for this important comment and for the comparison with Ref. [30]. We would like to clarify that our study provides a more direct and robust identification of the topological surface state (TSS). Below, we outline the key differences between Ref. [30] and the present work, which highlight the advances of our study.

(1) In Ref. [30], the identification of the TSS is primarily based on the dispersion of sharp spectral features. However, our combined bulk and surface band calculations show that such sharp features can arise from both surface and bulk states, indicating that dispersion alone is insufficient to unambiguously identify the TSS.

(2) In contrast, our study experimentally distinguishes surface and bulk states using spin-resolved laser ARPES. This approach enables a direct separation of the two contributions, whereas Ref. [30] does not explicitly distinguish between surface and bulk origins of the observed bands.

Finally, we would like to clarify a key point that may lead to different interpretations of the topological electronic structure in this system. Based on the magnetic symmetry of NdBi, the Dirac point of the TSS in antiferromagnetic phase, Domain-A (corresponding to Domain B in Ref. [30]) is symmetry-protected and therefore expected to remain gapless. However, Ref. [30] reports a sizable gap (~80 meV) even in this symmetry-protected domain, for which a clear physical explanation is not provided. This apparent discrepancy suggests that the observed gapped feature may not originate from the TSS itself. Instead, it can be naturally understood as arising from modifications of the bulk bands induced by the antiferromagnetic transition.

To clarify this point, we present new DFT calculations for the antiferromagnetic phase, Domain-A, in Fig. R3, which corresponds to the left half of Fig. R1. The calculations show that the TSS remains fully gapless in this domain (Figs. R3a3 and R3b3). In contrast, the bulk states undergo band folding in the antiferromagnetic phase, which significantly modifies their spectral weight (Figs. R3a2 and R3b2). As a result, these bulk band modifications produce an apparent gap-like feature of ~80 meV in the spectral function (Figs. R3a4 and R3b4).

Therefore, the large gaps at $\overline{\Gamma}$ and $\overline{\mathrm{M}}$ reported in Ref. [30] are most consistently understood as originating from bulk states. A key limitation of Ref. [30] is that it does not explicitly distinguish between bulk and surface contributions, which can lead to ambiguity in the interpretation of the data. In contrast, our study clearly separates the TSS from bulk contributions using high-resolution 7 eV laser ARPES, spin-resolved measurements, and DFT calculations, revealing the intrinsic and much smaller (~12 meV) gap of the TSS in the antiferromagnetic phase.

We believe that our results provide important insights into the topological electronic structure of antiferromagnets and establish a more reliable framework for distinguishing surface and bulk states in such systems, representing a significant advance of broad interest to the community.

We have added these arguments in the revised version of our manuscript.

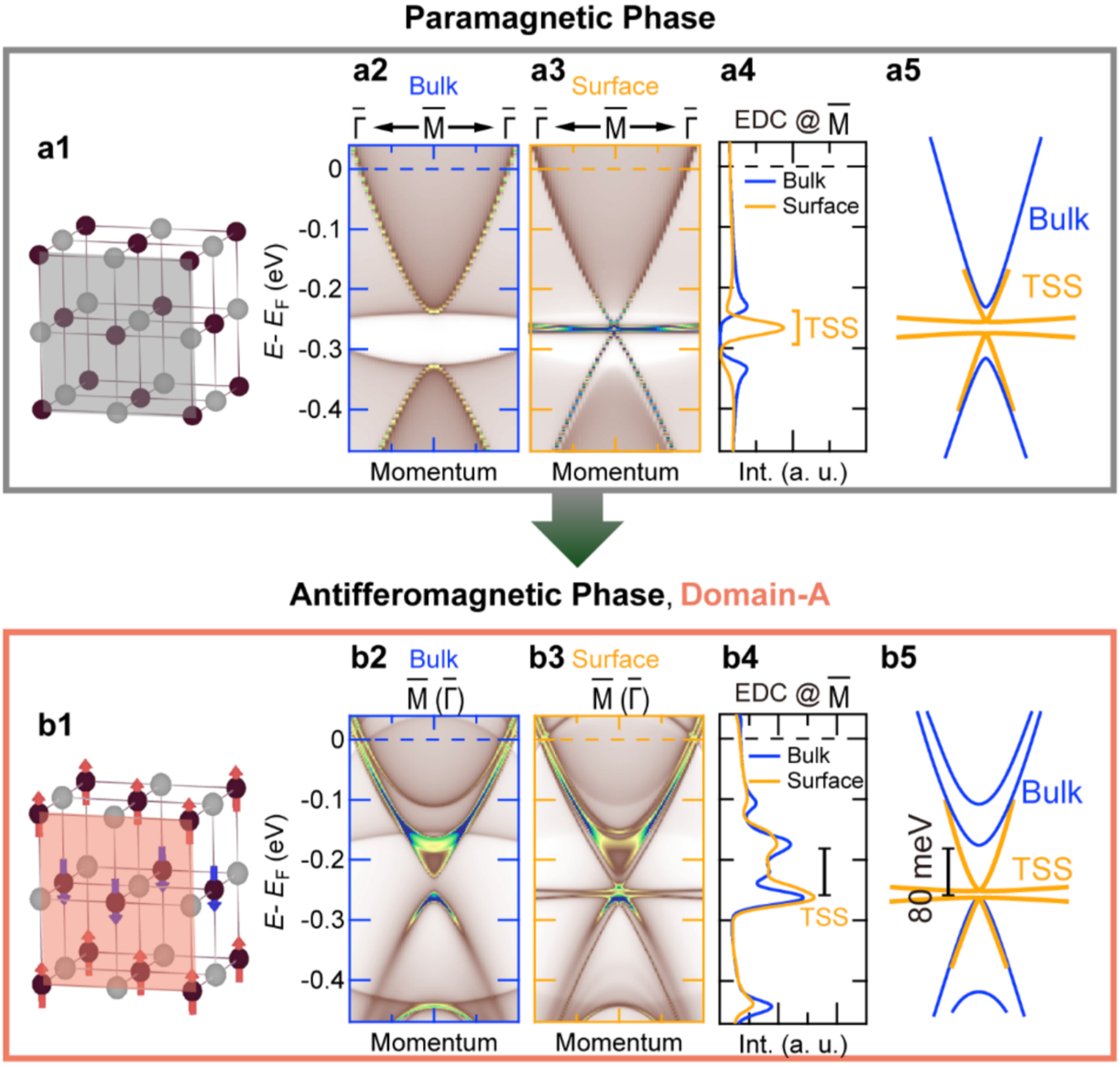

Figure R3 **Calculated electronic structures around the $\bar{M}$ point at (100) surface. a1–a5** Calculations for the paramagnetic phase. **b1–b5** Calculations for the antiferromagnetic phase (Domain-A).

Comment 1.6

5. The discussion related to Fig. 4(f) is very handwaving and lacks detailed support. How do the authors know experimentally the surface would lose magnetism without any surface degradation?

Answer:

We thank the reviewer for this important comment. Our interpretation is that the surface magnetic order is significantly suppressed by surface contamination, as indicated by the disappearance of the hybridization gap associated with magnetic order (see also the response to Comment 1.7).

At the same time, the spectral sharpness remains well preserved within the time scale of Fig. 5f, indicating that the surface quality is maintained and that the observed changes cannot be attributed to macroscopic surface degradation.

This contrast suggests that the surface magnetic order is more fragile than the surface electronic structure and can be suppressed even when the band dispersion remains well defined.

We have added these arguments in the revised version of our manuscript.

Comment 1.7

The authors claim that the hybridization between SS1 and TSS is directly related to the surface magnetism. Why is so? The strength of hybridization is directly linked to the wave function overlap and it is unclear to me why it is relate do surface magnetism.

Answer:

We note that band overlap alone does not necessarily lead to a hybridization gap. This is directly supported by our data near the magnetic transition temperature T_N, where the magnetic order vanishes, as shown in Fig. R4. The TSS and hole bands clearly cross without opening a discernible hybridization gap. This indicates that the effective coupling between these bands is negligible in the absence of magnetic order.

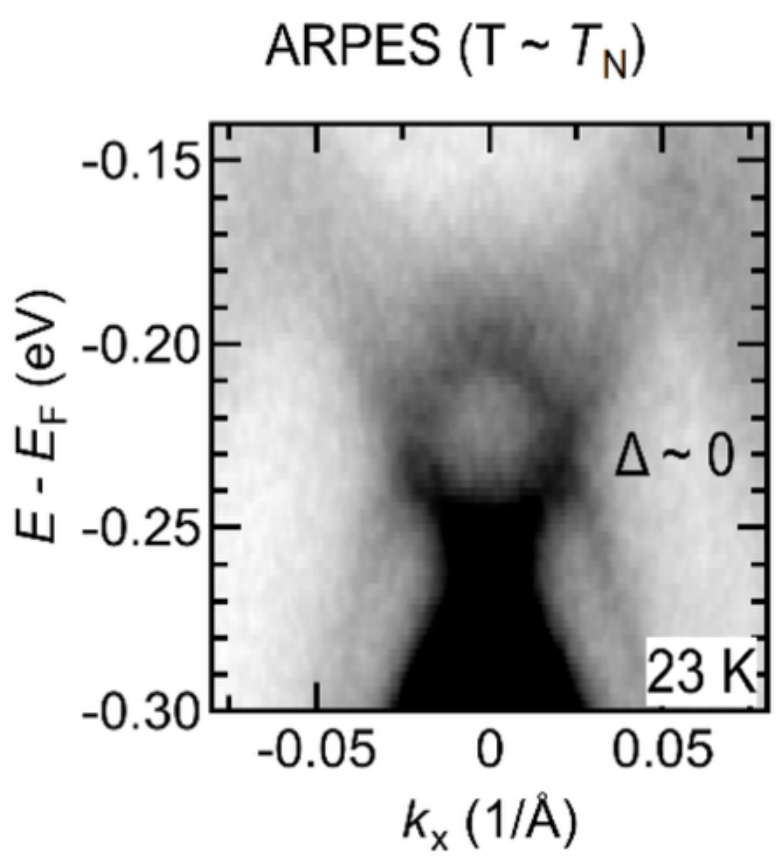

Figure R4: ARPES image observed at 23 K.

Notably, the exchange interaction, which gives rise to magnetic order, can introduce an off-diagonal matrix element that mixes the two bands, opening a hybridization gap at the band crossing. Our temperature-dependent measurements demonstrate that the hybridization gap opens only when magnetic order develops.

To establish a direct link between the hybridization gap and the magnitude of the magnetic order, we additionally performed neutron scattering measurements in response to the reviewer. In particular, we measured the temperature dependence of the integrated intensity of the 110 magnetic Bragg reflection I_{110}, whose square root $\sqrt{I_{110}}$ is proportional to the magnetic moment M. As demonstrated in Fig. R5b, we find an excellent agreement between the temperature evolutions of $\sqrt{I_{110}}$ and the experimentally observed hybridization gap. This provides compelling evidence that the gap opening originates from band hybridization induced by the exchange interaction associated with the magnetic order.

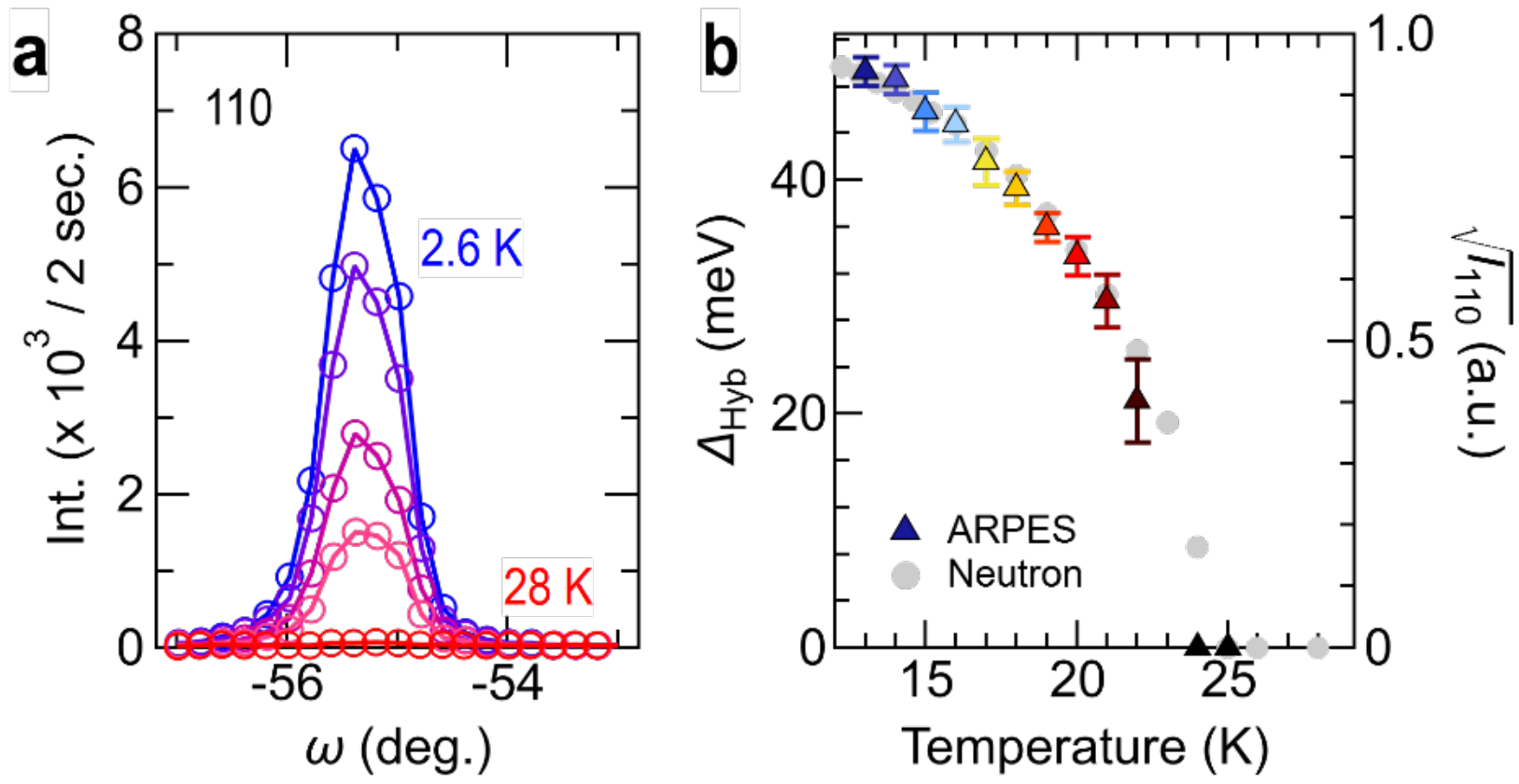

Figure R5: **Temperature dependence of the magnetic order parameter and the hybridization gap. a** Temperature dependence of the 110 magnetic reflection measured by neutron scattering. The horizontal axis ω corresponds to the sample rotation angle. **b** Comparison of the temperature dependence of the gap observed by ARPES (Δ_{Hyb}, left) and the square root of the integrated intensity of the 110 magnetic Bragg reflection ($\sqrt{I_{110}}$, right).

Based on these considerations, the disappearance of the gap observed in the surface contamination experiment (Fig. 5) can be attributed to the suppression of surface magnetic order. While some degree of surface degradation is inevitable, it must remain subtle on the timescale of our measurements, as evidenced by the persistence of sharp spectral features associated with the surface states, thereby ruling out trivial surface degradation as the origin of the gap collapse. Instead, our results most strongly suggest that the collapse of the hybridization gap arises from the disordering of the surface magnetic moments.

We have added these arguments in the revised version of our manuscript.

Comment 1.8

Also in Fig. 4(e), the SS1 band clearly becomes diminished with increasing time, and it seems to suggest that sample degradation can be important.

Answer:

In ARPES measurements, surface degradation is typically manifested as a broadening of the spectral peaks. To quantitatively assess this effect, we plot the energy distribution curves (EDCs) at the zone center for each panel in Fig. 5e. Our data clearly show that the spectral width remains nearly unchanged over time. This indicates that surface degradation is minimal within the time scale of the measurement. These results further indicate that the surface magnetic order is more fragile than the surface electronic structure and can be suppressed even when the band dispersion remains well defined.

We have added these arguments in the revised version of our manuscript.

Comment 1.9

Other minor issues:

1. Are calculations in Fig. 2 done in the PM phase? If so, it should be clearly explained.

Answer:

We thank the reviewer for this helpful comment. The calculations in Fig. 2 were performed in the paramagnetic (PM) phase. We agree that this was not sufficiently clear in the original manuscript, and we have now added a note in the revised version to clarify this point.

Comment 1.10

2. In Fig. 2, the gapped Dirac-cone like band centered at Gamma bar shows up (and carry out significant weight) in both the surface and bulk calculations. But this figure does not directly identify bulk and surface states. I would suggest that the authors do so for the sake of clarity.

Answer:

We thank the reviewer for this helpful suggestion. In the revised manuscript, we have explicitly identified the bulk and surface states, following the reviewer's suggestion. In particular, we clearly distinguish their contributions in the spectral function by providing separate bulk and surface band calculations and indicating them in the figures. We believe that this revision improves the clarity of the presentation.

Response to Reviwer #2:

Comment 2.1

The authors present high-resolution laser ARPES and spin-ARPES evidence for a spin-polarized,
The authors present high-resolution laser ARPES and spin-ARPES evidence for a spin-polarized,
gapped surface Dirac cone in the antiferromagnetic topological insulator (AFM-TI) NdBi. They identify a Dirac gap of ~15 meV, in close agreement with DFT (~12 meV), and argue that earlier reports of a much larger gap (~125 meV) likely misattributed bulk Dirac-like states to the topological surface state (TSS) due to photon-energy and resolution limitations. Temperature-dependent and time-evolution ("natural contamination") measurements reveal that the Dirac gap is magnetism-induced and depends on surface magnetic order, with a hybridization gap (Δhyb) following a 3D Ising-like order parameter.

Answer:

We thank the reviewer for the clear and accurate summary of our work and for highlighting the main findings of this study. Below, we provide a point-by-point reply to the reviewer's remarks and comments and have revised our manuscript accordingly.

Comment 2.2

2. Strengths

• Sub-3 meV resolution and spin selectivity clearly disentangle TSS from bulk states.

• Domain-resolved ARPES (AFM-A/B/C) properly accounts for magnetic symmetry and zone folding.

• Strong coherence between ARPES, spin-ARPES, DFT(+U), and Wannier-based surface calculations.

• Temperature and time evolution elegantly link surface magnetism to the Dirac gap.

• Credible reconciliation of AFM-TI inconsistencies (notably vs. $MnBi_{2n}Te_{3n+1}$) through photon-energy analysis.

Answer:

We thank the reviewer for the positive and insightful assessment, and for clearly highlighting the strengths of our work.

Comment 2.3

3. Major Comments

• Dirac gap quantification

Provide uncertainties, number of samples/cleaves, repeatability, and fitting details (line shapes, background, confidence intervals). Include error bars for gap values in main figures.

Answer:

We appreciate the reviewer's emphasis on the necessity of rigorous gap quantification. In response, we have performed a more detailed fitting analysis of the EDC at the $\bar{\Gamma}$ point of the AFM-C domain (Fig. 3d in the main manuscript) to evaluate the gap size and its associated uncertainty.

As illustrated in Fig. R6, we have fitted the EDC using a combination of three Gaussian functions with a linear background. This analysis estimates the gap size to be 17 ± 2 meV. Based on this result, we update our initial estimate of ~15 meV, which was derived from a simple peak-to-peak separation, to this more quantitatively evaluated value in the revised manuscript.

To assess reproducibility, we repeated the measurements on three independent sample cleaves and consistently obtained similar spectral features and gap magnitudes, which ensure the reliability of our results. The uncertainty was evaluated from the fitting procedure.

We have revised the manuscript accordingly to include these fitting details and have added error bars to the relevant figures.

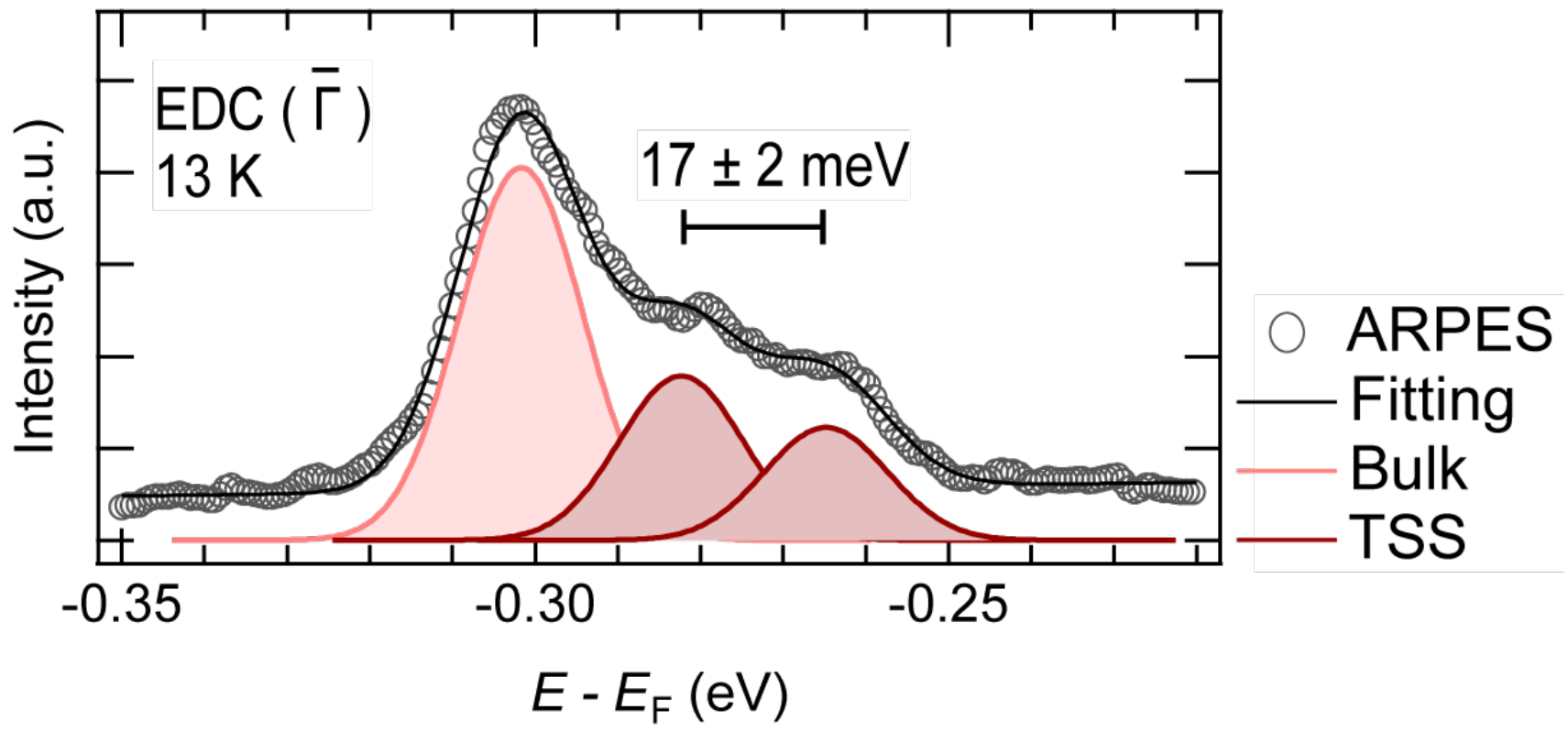

Figure R6 **Quantitative evaluation of the gap size at the $\bar{\Gamma}$ point.** Energy distribution curve (EDC) at the $\bar{\Gamma}$ point measured at 13 K. We fit the EDC with a combination of three Gaussian functions and a linear background. To ensure convergence, the widths of the three peaks were constrained to a single *parameter.*

Comment 2.4

• Spin-ARPES systematics
Report spin polarization vs. k and E with uncertainties; state Sherman function, asymmetry corrections, and resolution limits. A bulk-only reference cut showing negligible polarization would strengthen the argument.

Answer:
We thank the reviewer for the helpful comments regarding the spin-resolved ARPES analysis. We have clarified the experimental details and expanded the corresponding description in the revised manuscript.

Regarding the measurement parameters, we used an effective Sherman function of $S_{eff} = 0.27$. The VLEED-type spin detector operates by periodically switching the target magnetization during data acquisition, which effectively removes instrumental asymmetry. These details have now been included in the Methods section.

A quantitative analysis of the spin polarization as a function of energy at ±*k* is presented in Fig. R7 and has been added to the revised Supplementary Material. The error bars reflect the statistical uncertainty of the spin polarization derived from the counting statistics. The overall energy and momentum resolutions are described in the Methods section.
Regarding the suggestion of a bulk-only reference cut, we note that in the present system, even bulk-derived states can exhibit finite spin polarization due to the local breaking of inversion

symmetry at the surface, as shown in Fig. R3. Therefore, such a reference does not necessarily provide a zero-polarization baseline.

We have added these arguments and Fig. R7 in the revised version of our manuscript.

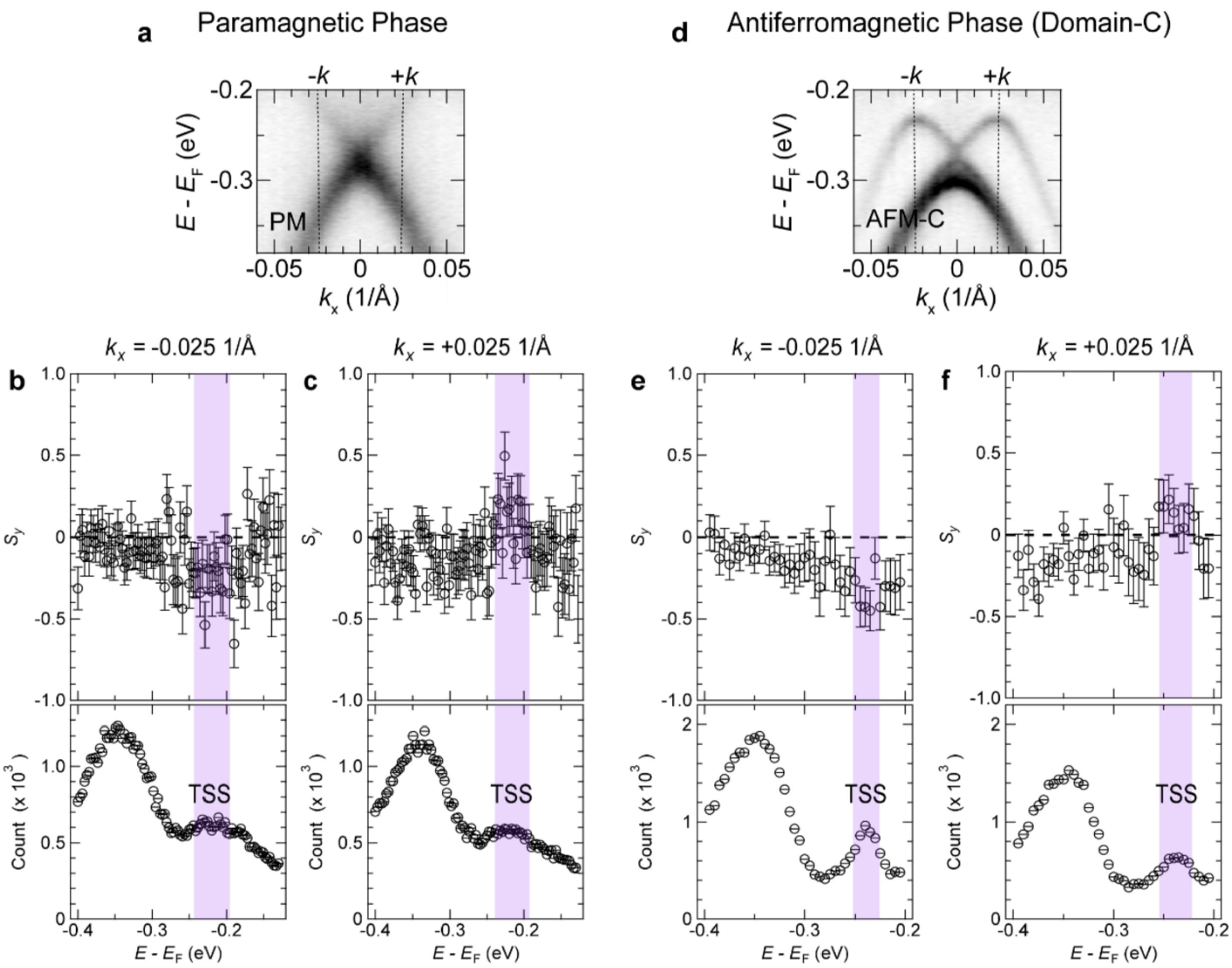

Figure R7 **Spin polarization analysis of the TSS in the paramagnetic and antiferromagnetic phases. a** ARPES intensity map along the $\overline{\Gamma} - \overline{M}$ direction in the paramagnetic (PM) phase. The dashed vertical lines indicate the momentum positions ($\pm k$) where spin-resolved ARPES were performed. **b, c** Spin polarization (upper panel) and spin-integrated spectra (bottom panel) at $+k$ and $-k$ for the PM phase, respectively. **d** ARPES intensity map in the antiferromagnetic phase, Domain-C (AFM-C). **e, f** Spin polarization (upper panel) and spin-integrated spectra (bottom panel) at $+k$ and $-k$ for the AFM-C phase, respectively. The purple shaded regions highlight the peaks originating from the topological surface states (TSS).

Comment 2.5

• Domain identification

Add a concise domain-mapping overview (beam scans, symmetry fingerprints, occurrence rates) and, if possible, real-space maps illustrating domain selection.

Answer:

We thank the reviewer for the helpful suggestion regarding domain identification. While a direct real-space domain map is not available in the present ARPES measurements and precise occurrence ratios have not been determined, we performed spatially resolved ARPES measurements by scanning the beam across the cleaved sample surface, enabling us to probe different regions of the sample and distinguish magnetic domains through their Fermi surface characteristics, as detailed below.

The magnetic domains were identified based on distinct symmetry fingerprints of the Fermi surface: Domains A and B exhibit twofold-symmetric Fermi surfaces, whereas Domain C displays fourfold symmetry. The domain assignment was performed by directly matching the measured Fermi surface patterns to these symmetry characteristics, providing an objective and unambiguous classification of the domains, as illustrated in the updated Fig. S3.

We further evaluated the occurrence of each domain across the scanned regions and found that multiple domains frequently coexist within a spatial scale comparable to the laser spot size, consistent with the spatially varying ARPES signals observed in the measurements. The combination of spatial scanning and symmetry-based classification effectively provides domain-resolved data for reliable magnetic domain identification.

These clarifications have been incorporated into the revised Supplementary Note 3.

Comment 2.6

• Photon-energy dependence
Move key hv-dependence data from the SI to the main text. Show 7/8/10 eV spectra side-by-side to highlight TSS suppression at higher photon energies.

Answer:

Following the reviewer's suggestion, we have moved the photon-energy-dependent data from the Supplementary Information to the main manuscript. In particular, we selected representative data at 8 eV and 10 eV (Fig. R8) for direct side-by-side comparison, which clearly demonstrates that the TSS is strongly suppressed at higher photon energies.

The full photon-energy-dependent dataset is provided in the Supplementary Information. We note, as detailed therein, that the relative ARPES intensity of surface and bulk states varies with photon energy due to photoemission matrix-element effects.

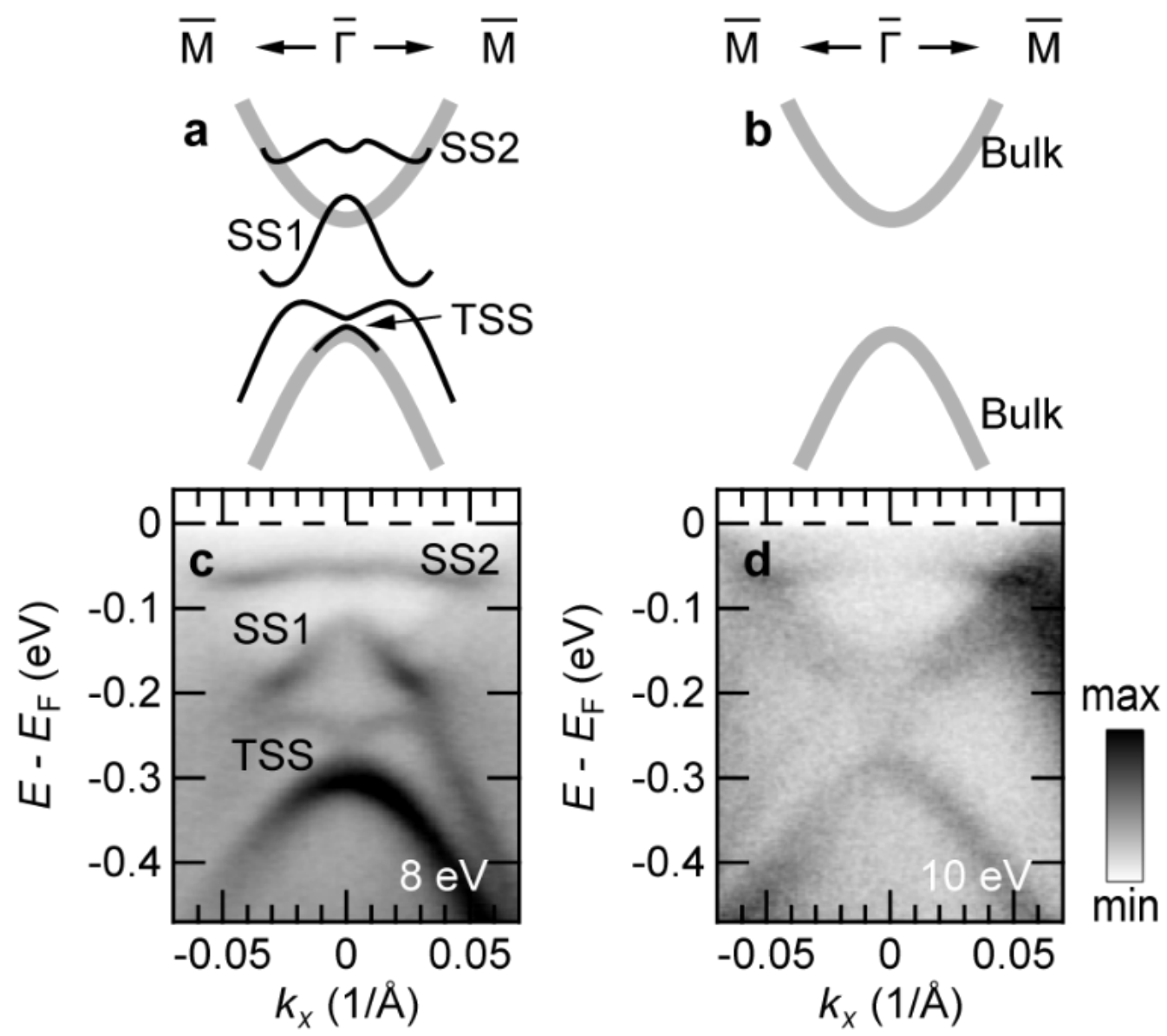

Figure R8 **Photon energy dependence of the electronic structure. a, b** Schematic band dispersions representing both the surface states (TSS, SS1, and SS2) and bulk states (**a**), and only the bulk states (**b**). **c, d** ARPES intensity maps along the $\overline{\Gamma}-\overline{M}$ direction measured with photon energies of 8 eV (**c**) and 10 eV (**d**).

Comment 2.7

• k_x–k_z dispersion
Demonstrate TSS k_z-independence (via photon-energy scans or limits). Even a compact confirmation would preempt alternative "quasi-2D bulk" interpretations.

Answer:

We thank the reviewer for this important comment. We agree that photon-energy-dependent measurements are commonly used to probe k_z dispersion and distinguish surface and bulk states. However, in the present system, a straightforward tracking of the band dispersion as a function of photon energy is challenging for the following reasons.

As shown in Fig. R8, the photoemission matrix element of the surface state varies strongly within a narrow photon-energy range. While the TSS is clearly resolved at $h\nu$ = 8 eV [Fig. R8c], its intensity is significantly suppressed at 10 eV [Fig. R8(d)], where bulk-derived states become dominant. This strong matrix-element effect makes it difficult to precisely track the peak positions of the surface state for a fair comparison at different photon energies.

In addition, we note that the synchrotron data exhibit a slight distortion in the band shape due to a minor imperfection in the analyzer lens setting. While this affects the precise determination of the band dispersion and prevents a fair comparison across different photon energies, it does not

affect the key observation that the surface-state intensity is strongly suppressed at 10 eV due to matrix element effects.

We emphasize that our main conclusions remain robust, as they are supported by spin-resolved ARPES measurements revealing spin-momentum locking, as well as by their reproducibility across different samples and measurement conditions. These observations provide strong evidence for the surface origin of the state, effectively excluding a quasi-2D bulk interpretation.

These points have been clarified in the revised manuscript.

Comment 2.8

• 3D Ising fit and TNT_NTN
Include fit uncertainties and goodness-of-fit metrics; overlay a 2D Ising comparison. Clarify how surface TNT_NTN was determined (it may deviate from bulk). If feasible, supplement with an independent magnetic probe.

Answer:
We appreciate the reviewer's constructive feedback.

For the fitting procedure, the surface T_{N} was treated as a free parameter, yielding a value of 22.8(7) K. The slight deviation (~1.2 K) from the bulk T_{N} $(24\ \mathrm{K})$ is likely attributable to minor experimental factors such as a thermal gradient between the sample holder and the sample surface, calibration offset of the thermometer, or potentially a small intrinsic reduction of T_{N} at the surface. In any case, this small difference does not affect our main conclusions.

Most importantly, as suggested by the reviewer, we have supplemented our ARPES results with an independent magnetic probe. We have updated Fig. 5c,d in the main manuscript to show a direct comparison with neutron scattering data; the same figures are shown in the reply Fig.R5. The temperature dependence of the gap Δ_{Hyb} scales remarkably well with the square root of the (110) magnetic reflection intensity ($\sqrt{I_{110}}$), confirming that the gap opening is directly driven by the magnetic order.

To address the reviewer’s comments, we have now included the fit uncertainties and a direct comparison with the 2D Ising model in the Supplementary Material.

Comment 2.9

• Surface magnetism corroboration
Support the contamination-driven closure of Δhyb and the Dirac gap with a surface-sensitive probe (XMCD, MOKE, SP-STM/STS, or ex-situ Kerr) on similarly prepared surfaces.

Answer:

We thank the reviewer for this important suggestion. We agree that surface-sensitive magnetic probes such as XMCD, MOKE, or SP-STM would provide valuable complementary information on the surface magnetic properties.

However, these techniques are momentum-integrated and do not allow direct access to the momentum-resolved electronic structure of the topological surface state (TSS), which is essential for identifying the hybridization gap and Dirac gap in the present study. In this work, ARPES uniquely enables us to directly resolve the TSS and track its evolution in momentum space.

In the revised manuscript, we performed neutron-scattering measurements that directly probe the magnetic order in the bulk and further support our interpretation. As shown in Fig. 5c,d (same as Fig. R5), the evolution of the magnetic order correlates with that of Δ_{Hyb}, providing independent evidence that the hybridization gap is intimately linked to magnetic order. This, together with the observed contamination-driven suppression of Δ_{Hyb} in ARPES, strongly suggests that the gap closure originates from the weakening of surface magnetism. The Dirac gap shows the same behavior, indicating that it also arises from surface magnetism.

While performing such complementary measurements is beyond the scope of the present study, it would be an interesting direction for future work.

Comment 2.10

• Theory robustness
Discuss sensitivity to U,JU, JU,J, and slab thickness; quantify the TSS decay-length reduction (PM ≈ 17 nm → AFM-C ≈ 2–4 nm) and comment on surface termination effects.

Answer:
To test the dependence on the U parameter, we performed an additional calculation using $U = 5.3$ eV in Fig. R9 (We used $U = 6.3$ eV in the main manuscript). The calculated decay lengths of the upper and lower parts of TSS with this parameter are 4~6 nm. This value confirms that the TSS in the AFM phase is more localized to the surface compared to the paramagnetic phase (~17 nm), regardless of the variation in U.

Regarding the surface termination, there is structurally only one surface termination along the (001) cleavage plane due to the NaCl structure. While the magnetic orientation of the topmost layer can be reversed, this simply inverts the sign of the S_z spin texture and does not affect the decay length. Therefore, the surface termination effect is not present in this system.

We have added these arguments and Fig. R9 in the revised version of our Supplementary Note 6.

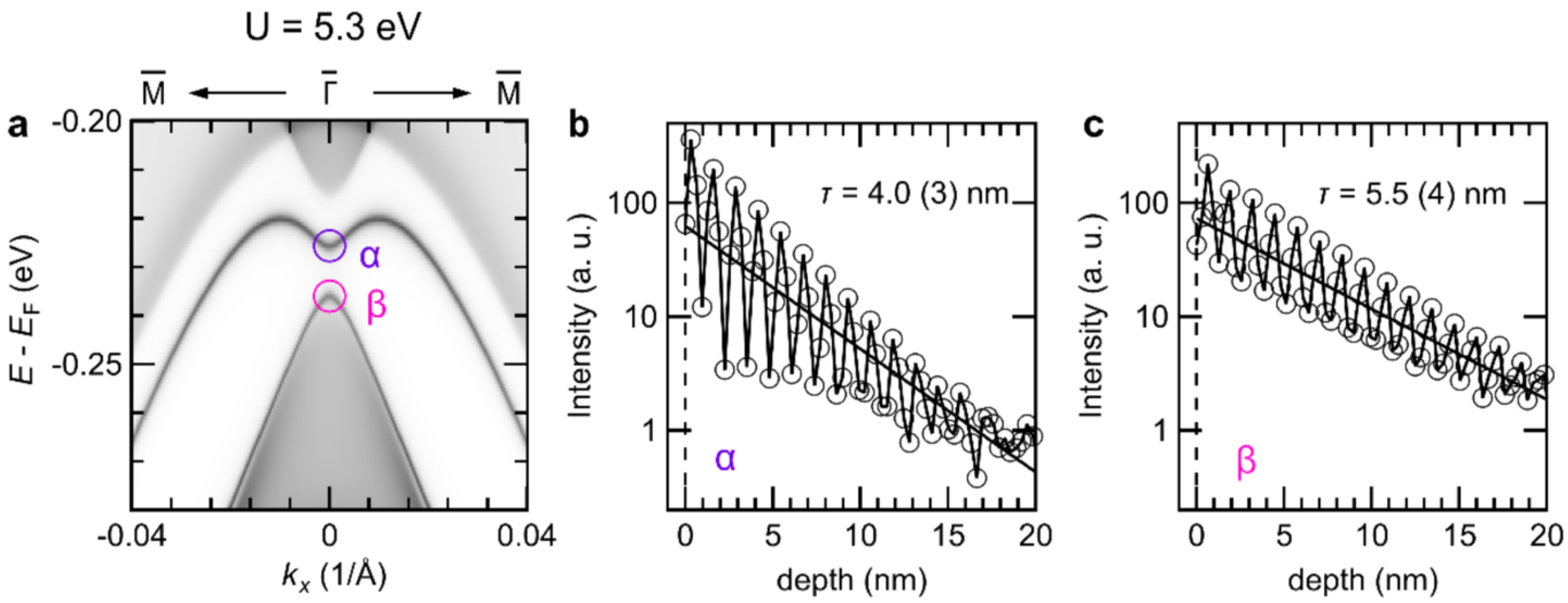

Figure R9 **Surface localization of the TSS calculated with U = 5.3 eV. a** Calculated band dispersion near the Dirac point using U = 5.3 eV. **b, c** Decay length of TSS, calculated in the momentum space labeled as α, β in Fig. R9(a), respectively. The decay length (τ) was determined by fitting the data with an exponential function, yielding τ = 4.0(3) nm for α and τ = 5.5(4) nm for β.

Comment 2.11

• Claim language
Soften to "decisive" or "unambiguous under spin- and energy-resolved conditions," specifying the measurement parameters (7 eV, ΔE = 2.5 meV, domain control).

Answer:
Following the reviewer's suggestion, we have softened the language throughout the manuscript. We have also explicitly specified the measurement conditions.

Comment 2.12

• Device relevance
Briefly relate gap and temperature scales to QAHE feasibility and compare to $MnBi_{2n}Te_{3n+1}$. Note prospects for thin films or electrostatic gating to enhance Δ and TNT_NTN.

Answer:
We appreciate the reviewer's suggestion to contextualize our findings in terms of potential device applications. In the revised manuscript, we have added a discussion comparing NdBi with $MnBi_2Te_4$.

$MnBi_2Te_4$ exhibits a relatively small gap (< 3 meV), which limits the observation of the quantum anomalous Hall effect (QAHE) to ultra-low temperatures (~1 K). In contrast, we observe a significantly larger gap (~17 meV) in NdBi, suggesting the possibility of realizing QAHE at elevated temperatures on the order of 10 K.

We further note that thin-film fabrication is a promising route toward device realization. Quantum confinement in thin films is expected to drive a semimetal-to-semiconductor transition while preserving both magnetism and band topology. In addition, electrostatic gating could provide further control of the chemical potential and enhance the effective gap, offering a viable pathway to optimize QAHE conditions.

These considerations have been discussed in the revised version of our manuscript.

Comment 2.13

4. Minor and Clarity Points

• Maintain consistent visual markers for TSS, SS1/SS2, and BVB; add inset momentum maps for orientation.

Answer:

We have revised the figures to ensure consistent visual labeling for the TSS, SS1/SS2, and bulk bands throughout the manuscript. Due to limited space, we have included momentum direction indicators (e.g., $\overline{\Gamma}$ and $\overline{\mathrm{M}}$), instead of adding inset momentum maps.

Comment 2.14

• Specify EDC fitting models (Voigt/Lorentzian), background type (Shirley/linear), and applied constraints.

Answer:

For the analysis of the TSS gap and the hybridization gap (Δ_{Hyb}), we employed a Gaussian function combined with a linear background to fit the Energy Distribution Curves (EDCs). No constraints were imposed on the fitting parameters.

We have specified the fitting details in the revised manuscript.

Comment 2.15

• In time-evolution data, show pressure history, elapsed times, and gap vs. time with uncertainties; confirm thermal stability.

Answer:

We have addressed the reviewer's request by providing detailed data on environmental stability and the quantitative evolution of the gap.

Although the specific pressure log file for the original measurement is unavailable, we established a new logging system to verify the experimental conditions. Figure R10(a) demonstrates that the base pressure remains highly stable at $< 5 \times 10^{-11}$ Torr over more than an hour at low temperatures, representing the typical pressure history during our measurements.

Regarding thermal stability, the temperature was strictly controlled via PID. As shown in Fig. R10(b), the standard deviation over a 30-minute period was 6.8 mK, confirming negligible thermal drift within the experimental time window. Furthermore, Figure R10(c) confirms that the temperature sweep rate of 1 K/min used in our experiments does not induce any pressure fluctuations.

To quantify the time evolution of the gap, we analyzed the ratio of the spectral intensity within the gap to that of the surface state, as plotted in Fig. R11(e), where the horizontal axis explicitly represents the elapsed time after cleavage. The data show a monotonic increase in the in-gap intensity over time, quantitatively confirming progressive spectral filling that leads to a gap closure after ~4 hours. The uncertainties in the intensity ratio were estimated from the statistical variation of the integrated spectral weight and are included in Fig. R11(e).

In the revised manuscript, we added these details with Fig. R10 and Fig. R11.

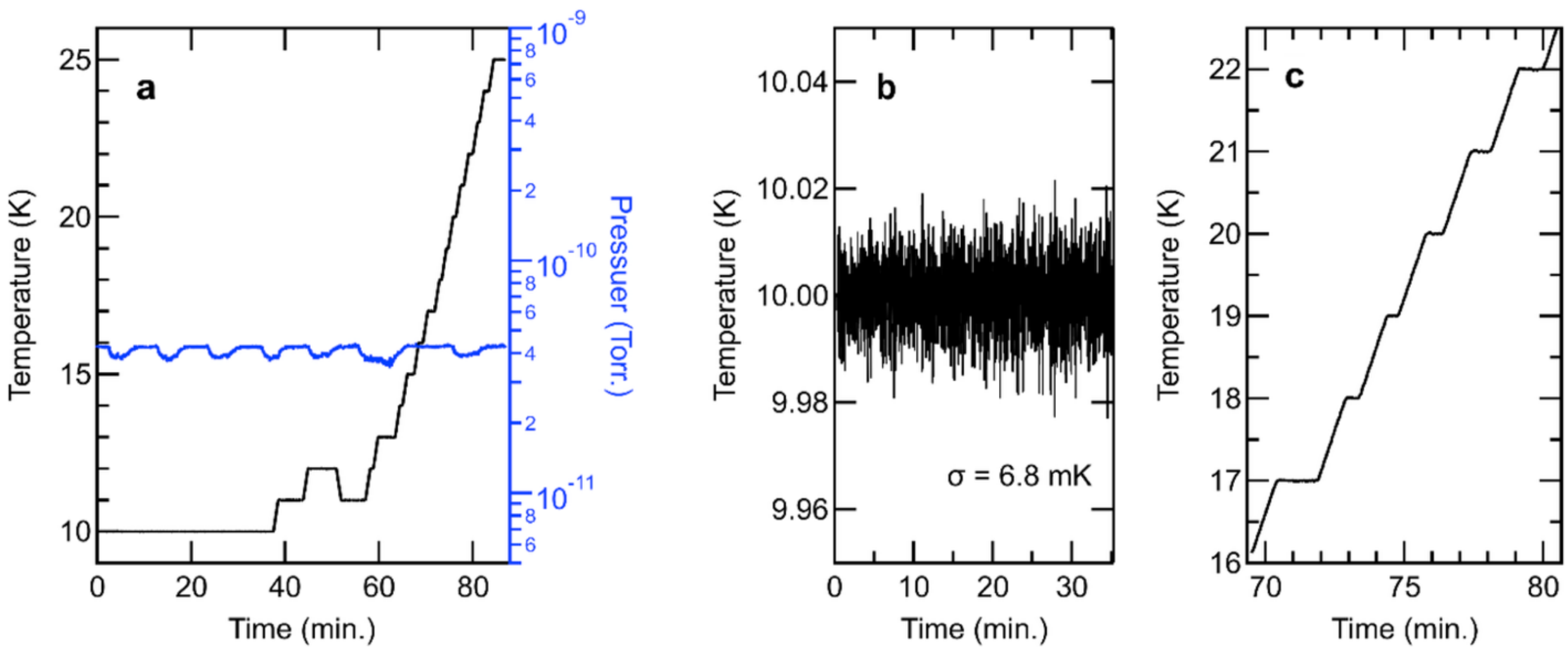

Figure R10 **Stability of sample temperature and vacuum conditions. a** Time evolution of the sample temperature (left) and vacuum pressure (right) under PID control. **b** Temperature stability maintained at 10 K. The standard deviation over a 30 minute is 6.8 mK, **c** Temperature profile during a controlled ramp at a rate of 1 K/min.

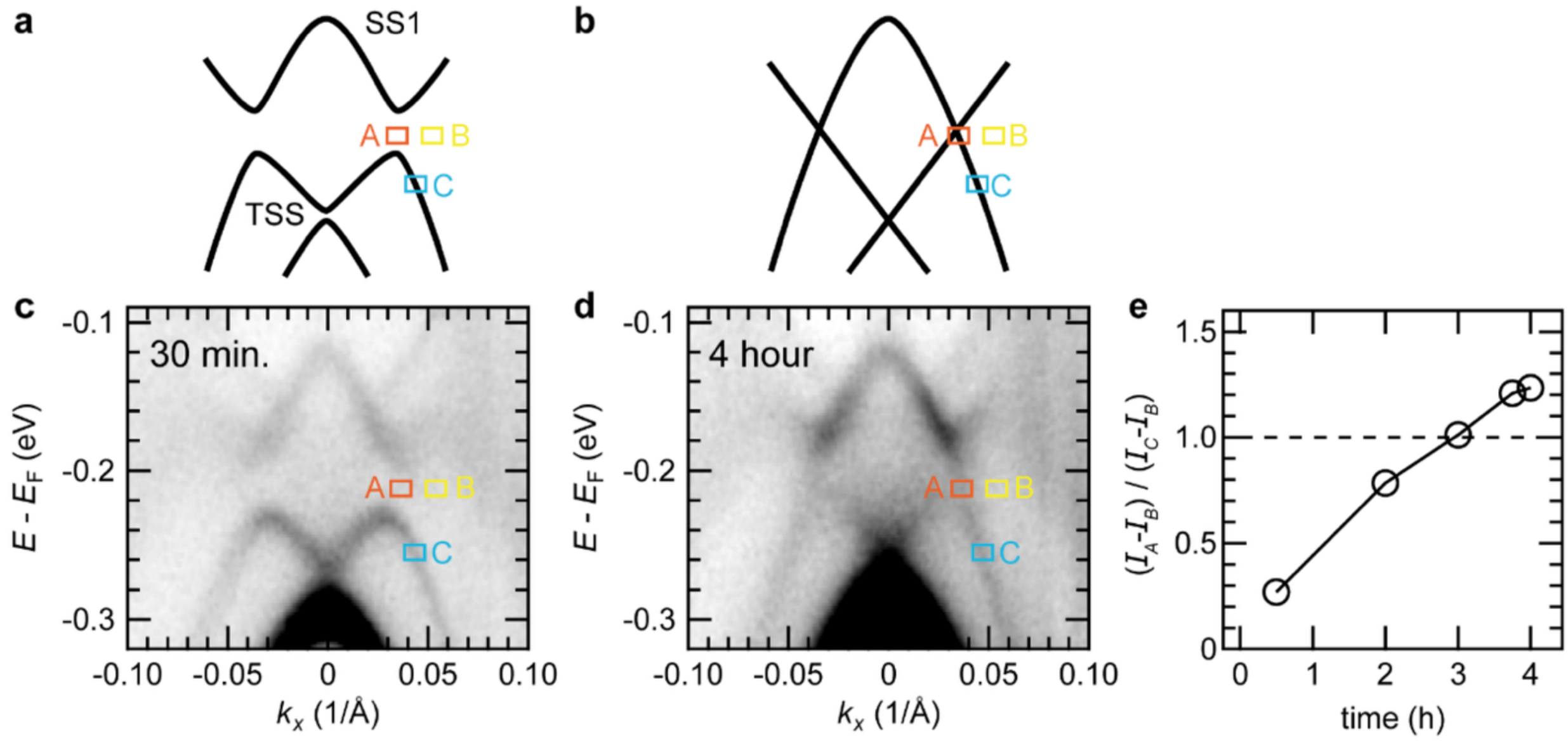

Figure R11 **Surface aging effect on the electronic structure. a, b** Schematics of the surface state for a clean surface(**a**) and a contaminated surface (**b**). On the clean surface, a gap Δ_{Hyb} is formed between the topological surface state (TSS) and the surface state (SS1), whereas the gap collapses upon surface contamination. **c, d** ARPES intensity maps measured at 30 minutes (**c**) and 4 hours (**d**) after cleavage. **e** Time evolution of the photoelectron intensity within the gap. The intensity was calculated from the integrated intensity of A-C shown in **c** and **d**.

Comment 2.16

• Methods: report cleave temperature, surface termination statistics, beam-spot size vs. domain size, and sample-to-sample reproducibility.

Answer:

We have updated the Methods section in the revised manuscript to include all the requested experimental details. Specifically, for temperature-dependent measurements, the samples were cleaved at the lowest temperature (~13 K), whereas for other measurements, cleavage was performed at the measurement temperature. The beam spot size was approximately 50 μm, which is significantly smaller than the typical magnetic domain size (>200 μm, Supplementary Note 2), ensuring that the measurements predominantly probe a single magnetic domain.

In addition, a total of 38 samples were cleaved and measured for ARPES. Although the data quality varies from sample to sample, the main results presented in the manuscript were found to be highly reproducible.

Regarding the request for surface termination statistics, we note that NdBi has a rock-salt crystal structure, for which a single crystallographic termination is expected upon cleavage. Consistently, we did not observe any evidence of multiple surface terminations in our measurements, and therefore, surface termination statistics are not applicable in this case.

Comment 2.17

• Deposit raw ARPES/momentum maps and DFT/Wannier inputs for reproducibility.

Answer:
We agree with the reviewer's suggestion regarding data reproducibility. We will deposit the raw ARPES data and the DFT/Wannier inputs in a public repository upon publication of the manuscript

Comment 2.18

5. Important Controls

• Capping/dosing tests: Controlled O_2/H_2O exposure to quench and recover surface magnetism after mild annealing.

Answer:
We thank the reviewer for this interesting suggestion. While controlled O_2/H_2O dosing experiments could provide additional insights into surface effects, we believe that such experiments are beyond the scope of the present study, which focuses on the intrinsic electronic and magnetic properties of NdBi. Importantly, our conclusions are based on systematic and reproducible measurements under well-controlled vacuum conditions.

Comment 2.19

• Thickness dependence: Examine flakes or varying cleave thicknesses to assess TSS localization and gap evolution.

Answer:
We thank the reviewer for this important suggestion. While thickness-dependent measurements could in principle provide additional insight into the spatial localization of the TSS, we note that our conclusions are based on surface-sensitive ARPES measurements combined with spin-resolved data, which already provide a direct and unambiguous identification of the surface state.

Moreover, the observed gap and its characteristics are consistent across multiple samples and cleaves, indicating that the results are robust and do not show any systematic dependence on sample thickness within our experimental conditions. Therefore, we believe that such measurements, while interesting, are beyond the scope of the present study.

Comment 2.20

• Magnetic-field dependence: Apply modest out-of-plane fields to test Dirac gap alignment sensitivity.

Answer:

We thank the reviewer for this valuable suggestion. While magnetic-field-dependent measurements could provide additional insight into the Dirac gap behavior, we note that our conclusions are based on spin-resolved ARPES measurements and domain-dependent analyses, which already establish the magnetic origin of the gap.

Moreover, the observed gap is robust and reproducible across different samples and measurement conditions, indicating that the key features are intrinsic. Therefore, while such experiments are interesting, we believe that they are beyond the scope of the present study.

Comment 2.21

6. Presentation

• Shorten the Abstract to emphasize:
(1) 15 meV spin-polarized surface gap;
(2) agreement with DFT;
(3) disentanglement of bulk/surface via 7 eV laser ARPES (ΔE = 2.5 meV) and spin-ARPES;
(4) linkage between surface magnetism and Dirac gap through temperature and contamination control.

Answer:
We have revised the Abstract in accordance with the reviewer's suggestion

Comment 2.22

• Standardize notation (TSS, BVB, Δhyb, TNT_NTN) and units (meV, eV, K).

Answer:
We followed the reviewer's suggestion in the revised version of our manuscript.

Response to Reviwer #3:

Comment 3.1

The manuscript "Direct observation of a spin-polarized surface Dirac gap in the antiferromagnetic topological insulator NdBi" by Yuto Fukushima et al. reports a laser-ARPES study of the antiferromagnetic topological insulator NdBi. The authors identify a Dirac-like surface state with an energy gap of approximately 15 meV, and attribute the gap opening to cooperative exchange interactions involving both bulk and surface magnetism, mediated by coupling between localized and itinerant carriers.

The manuscript is well written, and the figures display high-quality ARPES data with excellent resolution. The topic is timely and relevant to the community working on magnetic topological materials and correlated systems.

However, the novelty and context of this work require clearer articulation. In particular, this study shows significant overlap with the recent paper of A. Honma et al., Nature Communications 14, 7396 (2023), which reports that NdBi hosts two distinct surface electronic states depending on the orientation of the AFM domain, but with a much larger surface Dirac gap (>100 meV) under specific magnetic configurations.

This similarity with the metioend earlier work is not problematic in itself; indeed, complementary experiments using different photon energies that probe different depths and higher energy resolution can be valuable for clarifying the electronic and magnetic landscape. However, for readers to obtain a coherent and complete physical picture, the authors must:

Answer:
We thank the reviewer for the positive and insightful comments on our manuscript. We are particularly pleased that the reviewer recognizes the value of our high-resolution, depth-sensitive measurements in clarifying the electronic and magnetic landscape of NdBi.

We also appreciate the reviewer's important point regarding the relationship between our results and the recent work by Honma et al. We have carefully addressed these issues following the reviewer's suggestions, as detailed below, and revised our manuscript accordingly.

Comment 3.2

1. Explicitly and more directly discuss the previous Nature Communications work in the Introduction.

Answer:
We have revised the Introduction to explicitly and more directly discuss the previous work by Honma et al. [**Nature Communications 14, 7396 (2023)**], including the key open issues that motivated the present study. In particular, we now clearly position our results in relation to their findings, highlighting the key differences.

Comment 3.3

2. Clarify how the present data relate to the scenario reported earlier.

Answer:
We thank the reviewer for this important point. The relationship between our results and the previous study can be clarified as follows.

Ref. [30] reports a Dirac gap of approximately 125 meV for the topological surface state, which is an order of magnitude larger than the value predicted by our DFT calculations. In that work, however, the separation between surface and bulk states remains ambiguous, which makes it difficult to isolate the intrinsic properties of the topological surface state.

In contrast, our study combines high-resolution laser ARPES with spin-resolved measurements, allowing us to unambiguously distinguish surface and bulk contributions. This enables a direct determination of the intrinsic surface Dirac gap, which we find to be consistent with theoretical expectations.

Furthermore, we note that the previous study reports a large Dirac gap even for surface orientations where a gap is not expected from symmetry considerations. In our DFT calculations, this issue is resolved, and the observed behavior can be consistently explained within a framework that takes into account both surface and bulk band.

In addition, by employing controlled surface contamination, we demonstrate that the Dirac gap is strongly sensitive to surface magnetism. In particular, we observe that suppressing surface magnetism leads to a closing of the Dirac gap, providing direct evidence for its magnetic origin and capturing an essential aspect of the antiferromagnetic topological insulator.

We have revised the manuscript to clarify this relationship and to more clearly highlight the advancement over the previous study.

Comment 3.4

3. Provide a more rigorous comparison of the magnitude, origin, and conditions of the Dirac gap.

Answer:

We thank the reviewer for this important request. We have revised the manuscript to provide a more rigorous comparison of the magnitude, origin, and conditions of the Dirac gap.

Regarding the magnitude, Ref. [30] reports a Dirac gap of approximately 125 meV, whereas our measurements yield a significantly smaller value that is consistent with DFT calculations, indicating that we capture the intrinsic Dirac gap of an antiferromagnetic topological surface state.

Concerning the origin, our spin-resolved ARPES measurements, combined with controlled surface contamination, demonstrate that the Dirac gap is directly linked to surface magnetism. In particular, we observe that suppressing surface magnetism closes the Dirac gap, providing direct evidence of its magnetic origin.

As for the conditions under which the gap appears, we show that the Dirac gap depends sensitively on both surface magnetic order and the experimental probing conditions, including photon energy and surface sensitivity. This explains the apparent discrepancy with previous

studies, in which bulk contributions may have influenced the interpretation of the data on the Dirac gap size.

These points are now clearly described in the revised manuscript.

Comment 3.5

Revising the manuscript in this direction is essential to deliver to the community a more complete and integrated understanding of the NdBi system and to avoid the impression of parallel or partially redundant conclusions. In doing so, the authors will strengthen the scientific value and impact of the present work.

Answer:

We thank the reviewer for this important suggestion. The key advances of the present study are as follows. First, by combining high-resolution laser ARPES with spin-resolved measurements, we unambiguously distinguish surface and bulk states, enabling a direct determination of the intrinsic surface Dirac gap. Second, through controlled surface contamination, we demonstrate that the Dirac gap is governed by surface magnetism and directly show that suppressing surface magnetism leads to a closing of the Dirac gap. Third, we resolve the previously reported inconsistencies in the magnitude and symmetry dependence of the Dirac gap, providing a unified interpretation based on the interplay between surface and bulk magnetism. We believe that these results provide a more complete and integrated understanding of the NdBi system and significantly strengthen the scientific value and impact of the present work.

In light of the above arguments, we have revised the manuscript to more clearly articulate what is new and significant in our work and to situate it within the broader context of previous studies.

Comment 3.6

Finally, I strongly recommend avoiding claims such as "for the first time" or similar priority statements.

Answer:

We thank the reviewer for this helpful suggestion. We have removed such priority statements throughout the manuscript, following the reviewer's recommendation.

To clarify the key novelty of our work, we note here that our study provides an unambiguous experimental demonstration that the surface Dirac gap in NdBi is intrinsically governed by surface magnetism, enabled by the combined use of spin-resolved and high-resolution laser ARPES, together with controlled surface contamination experiments that directly reveal the magnetic origin of the gap. This represents a key advance beyond the previous study by Honma et al. [Ref. 30].
