# Supplementary material for: Direct observation of a spin-polarized surface Dirac gap in the antiferromagnetic topological insulator NdBi
Source: Nat Commun. 2026 Sep 15;17:10841. doi: 10.1038/s41467-026-77571-4 (PMC13672286; doi:10.1038/s41467-026-77571-4)
Supplement: Supplementary file 3 [file 41467_2026_77571_MOESM3_ESM.pdf]

Description of Additional Supplementary Files

Supplementary Movie 1: Temperature evolution of ARPES images in the AFM-C domain from 25 K (paramagnetic phase) to 13 K (AF phase), showing the emergence of surface states and the opening of the hybridization gap.
